# Supplementary material for: Associations between socioeconomic position and changes in children’s screen-viewing between ages 6 and 9: a longitudinal study
Source: BMJ Open. 2019 Dec 10;9(12):e027481. doi: 10.1136/bmjopen-2018-027481 (PMC6924727; doi:10.1136/bmjopen-2018-027481)
Supplement: Supplementary data [file bmjopen-2018-027481supp001.pdf]

SUPPLEMENTARY MATERIAL

ASSOCIATIONS BETWEEN SOCIO-ECONOMIC POSITION AND CHANGES IN CHILDREN'S SCREEN-VIEWING BETWEEN AGES 6 AND 9: A LONGITUDINAL STUDY

Ruth Salway, Lydia Emm-Collison, Simon J. Sebire, Janice L. Thompson and Russell Jago

List of Supplementary Tables

Table S1 – Characteristics at age 6

Table S2 - Characteristics at age 9

Table S3 – Missing values

Table S4 – full details of adjusted model for screen-viewing at age 6

Table S5 – full details of adjusted model for screen-viewing at age 9

Table S6– Associations between socioeconomic position and multi-screen-viewing at age 9

List of Supplementary Figures

Figure S1: Number of minutes spent multi-screen viewing by household education at age 9, for weekdays (left) and weekends (right).

Table S1 – Characteristics at age 6

	Up to GCSE or equiv	A Levels or equiv	Degree or equiv	Higher Degree or equiv	All
	Mean (sd) or %	Mean (sd) or %	Mean (sd) or %	Mean (sd) or %	Mean (sd) or %
WEEKDAY					
Total SV (mins)	120 (74)	111 (61)	93 (60)	85 (70)	102 (66)
TV (mins)	73 (53)	69 (45)	60 (41)	53 (44)	64 (46)
Computer (mins)	25 (28)	23 (27)	20 (22)	23 (33)	22 (26)
Games consoles (mins)	23 (30)	18 (20)	14 (25)	10 (20)	16 (24)
WEEKEND					
Total SV (mins)	196 (118)	193 (107)	169 (94)	150 (102)	178 (105)
TV (mins)	117 (78)	120 (72)	110 (69)	89 (68)	111 (72)
Computer (mins)	37 (44)	34 (38)	32 (33)	37 (39)	34 (37)
Games consoles (mins)	43 (55)	40 (45)	28 (39)	24 (39)	33 (45)
IMD	19.0 (15.5)	16.6 (13.7)	12.2 (10.4)	10.4 (7.4)	14.6 (12.5)
Female	45%	47%	50%	47%	48%
BMI z-score	0.49 (1.04)	0.18 (0.88)	0.16 (0.88)	0.24 (0.94)	0.23 (0.93)
No. devices in home:					
TVs	2.7 (1.3)	2.6 (1.3)	2.2 (1.1)	1.7 (1.1)	2.3 (1.2)
Computers	1.6 (1.1)	1.6 (0.9)	1.9 (1.0)	2.0 (1.1)	1.8 (1.0)
Tablets	0.4 (0.7)	0.5 (0.8)	0.5 (0.7)	0.5 (0.7)	0.5 (0.7)
Games consoles	2.8 (1.8)	2.8 (1.7)	2.0 (1.6)	1.3 (1.3)	2.3 (1.7)
Parent SV (mins)					
Weekday	231 (141)	230 (134)	220 (112)	202 (115)	222 (125)
Weekend	253 (146)	258 (124)	228 (103)	209 (113)	235 (120)
Parental limits on SV[1]	3.3 (0.6)	3.3 (0.6)	3.4 (0.6)	3.5 (0.6)	3.3 (0.6)

[1] higher values indicate more limits on screen viewing (between 1 and 4)

Table S2 - Characteristics at age 9

	Up to GCSE or equiv	A Levels or equiv	Degree or equiv	Higher Degree or equiv	All
	Mean (sd) or %	Mean (sd) or %	Mean (sd) or %	Mean (sd) or %	Mean (sd) or %
WEEKDAY					
Total SV (mins)	139 (94)	133 (78)	113 (75)	88 (59)	118 (78)
TV (mins)	75 (55)	72 (49)	63 (46)	48 (38)	65 (48)
Computer (mins)	37 (46)	34 (37)	31 (37)	29 (31)	32 (37)
Games consoles (mins)	28 (44)	26 (34)	19 (30)	12 (24)	21 (33)
Multi-screen viewing (mins)	29 (46)	24 (40)	16 (33)	11 (25)	19 (37)
WEEKEND					
Total SV (mins)	244 (134)	245 (120)	227 (118)	200 (118)	230 (122)
TV (mins)	127 (72)	126 (66)	125 (63)	104 (62)	122 (65)
Computer (mins)	59 (66)	58 (64)	53 (60)	57 (54)	56 (61)
Games consoles (mins)	58 (68)	62 (63)	49 (62)	39 (54)	52 (62)
Multi-screen viewing (mins)	53 (73)	48 (66)	33 (61)	23 (45)	39 (63)
IMD	20.1 (15.8)	17.6 (15.0)	12.8 (11.9)	12.0 (10.3)	15.1 (13.6)
Female	56%	52%	56%	53%	55%
BMI z-score	0.44 (1.15)	0.28 (1.04)	0.22 (1.06)	0.28 (1.02)	0.31 (1.06)
No. devices in the home					
TVs	2.9 (1.3)	3.1 (1.4)	2.4 (1.3)	1.8 (1.1)	2.6 (1.4)
Computers	1.6 (1.2)	1.7 (1.1)	2.0 (1.2)	2.2 (1.1)	1.9 (1.2)
Tablets	2.1 (1.2)	2.3 (1.3)	2.1 (1.2)	2.0 (1.2)	2.2 (1.3)
Games consoles	2.6 (2.1)	2.8 (1.8)	2.2 (1.6)	1.7 (1.6)	2.3 (1.8)
Parent SV (mins)					
Weekday	248 (134)	245 (128)	248 (134)	253 (144)	248 (134)
Weekend	280 (154)	287 (135)	260 (130)	252 (144)	270 (138)
Parental limits on SV[1]	3.1 (0.7)	3.2 (0.6)	3.4 (0.6)	3.4 (0.5)	3.3 (0.6)

[1] higher values indicate more limits on screen viewing (between 1 and 4)

Table S3 – Missing values

	Age 6		Age 9		Data at both time points: Age 6		Age 9	
	N	% missing	N	% missing	N	% missing	N	% missing
Total	1085		997		509		509	
Household education	1071	1%	992	0.2%	509	0%	509	0%
IMD	1069	1%	983	1%	501	2%	499	2%
**WEEKDAY**								
Total SV (mins)	1069	1%	979	2%	504	1%	502	1%
Multi-SV (mins)			981	2%			503	1%
Parent SV (mins)	1075	1%	981	2%	505	1%	503	1%
Parent multi-SV (mins)			983	1%			503	1%
**WEEKEND**								
Total SV (mins)	1065	2%	978	2%	500	2%	501	2%
Multi-SV (mins)			981	2%			503	1%
Parent SV (mins)	1068	2%	979	2%	505	1%	501	2%
Parent multi-SV (mins)			983	1%			503	1%
Gender	1085	0%	997	0%	509	0%	509	0%
BMI z-score	1072	1%	993	0.4%	502	1%	508	0.2%
No. TVs	1073	1%	982	2%	507	0.4%	503	1%
No. Computers	1058	2%	982	2%	504	1%	503	1%
No. Tablets	1062	2%	981	2%	504	1%	503	1%
No. games consoles	1056	3%	981	2%	500	2%	503	1%
Parental limits on SV	1064	2%	971	3%	495	3%	497	2%

Table S4 – full details of adjusted model for screen-viewing at age 6

	WEEKDAY			WEEKEND		
	Ratio	95% CI	p-value	Ratio	95% CI	p-value
Household education						
Up to GCSE	1	(Reference)		1	(Reference)	
A level	0.98	(0.89, 1.08)		1.03	(0.95, 1.12)	
Degree	0.88	(0.78, 1.00)		1.02	(0.93, 1.13)	
Higher degree	0.88	(0.74, 1.05)	0.054	0.99	(0.87, 1.11)	0.725
Deprivation (IMD)[1]	1.02	(0.98, 1.07)	0.296	1.01	(0.96, 1.06)	0.720
>1 TV	0.99	(0.90, 1.10)	0.924	1.04	(0.95, 1.13)	0.394
Any computers	0.94	(0.74, 1.21)	0.645	0.86	(0.73, 1.02)	0.080
Any tablets	1.02	(0.94, 1.10)	0.652	1.01	(0.94, 1.08)	0.811
Any games consoles	1.15	(1.00, 1.32)	0.043	1.42	(1.26, 1.60)	<0.0005
Child gender	0.87	(0.81, 0.94)	<0.0005	0.82	(0.78, 0.87)	<0.0005
z-BMI score	1.07	(1.02, 1.12)	0.003	1.04	(1.01, 1.07)	0.021
parent SV (mins)[2]	1.04	(1.03, 1.05)	<0.0005	1.05	(1.04, 1.07)	<0.0005
Parental limits on SV	0.77	(0.72, 0.83)	<0.0005	0.85	(0.81, 0.90)	<0.0005

[1] increase per 1 standard deviation (14.0) in IMD: higher values indicate more deprived areas

[2] increase per 30 minutes screen-viewing

Table S5 – full details of adjusted model for screen-viewing at age 9

	WEEKDAY			WEEKEND		
	Ratio	95% CI	p-value	Ratio	95% CI	p-value
Household education						
Up to GCSE	1	(Reference)		1	(Reference)	
A level	1.16	(1.03, 1.32)		1.14	(1.01, 1.28)	
Degree	1.10	(0.96, 1.26)		1.11	(0.99, 1.24)	
Higher degree	0.90	(0.77, 1.05)	0.008	0.98	(0.87, 1.11)	0.006
Deprivation (IMD)[1]	1.05	(1.00, 1.10)	0.038	1.00	(0.95, 1.05)	0.938
Baseline SV [2]	1.08	(1.05, 1.10)	<0.0005	1.05	(1.03, 1.06)	<0.0005
>1 TV	1.16	(1.06, 1.27)	0.001	1.06	(0.96, 1.18)	0.249
Any computers	1.03	(0.86, 1.24)	0.721	1.07	(0.92, 1.26)	0.386
Any tablets	0.75	(0.64, 0.88)	<0.0005	0.80	(0.68, 0.95)	0.009
Any games consoles	1.26	(1.11, 1.44)	<0.0005	1.27	(1.08, 1.49)	0.003
Child gender	0.98	(0.91, 1.06)	0.517	0.89	(0.83, 0.96)	0.002
z-BMI score	1.00	(0.95, 1.05)	0.966	1.00	(0.96, 1.03)	0.904
parent SV (mins)[2]	1.03	(1.02, 1.04)	<0.0005	1.04	(1.03, 1.05)	<0.0005
Parental limits on SV	0.82	(0.76, 0.89)	<0.0005	0.93	(0.86, 1.01)	0.084

[1] increase per 1 standard deviation (14.0) in IMD: higher values indicate more deprived areas

[2] increase per 30 minutes screen-viewing at age 6

[3] increase per 30 minutes screen-viewing

Table S6– Associations between socioeconomic position and multi-screen-viewing at age 9

	WEEKDAY			WEEKEND		
	Ratio	95% CI	p-value	Ratio	95% CI	p-value
**Unadjusted Model**						
Household education						
Up to GCSE	1	(Reference)		1	(Reference)	
A level	0.82	(0.49, 1.37)		0.87	(0.53, 1.42)	
Degree	0.66	(0.38, 1.15)		0.63	(0.37, 1.06)	
Higher degree	0.52	(0.26, 1.05)	0.226	0.55	(0.31, 0.97)	0.083
Deprivation (IMD)[1]	1.42	(1.21, 1.67)	<0.0005	1.33	(1.16, 1.52)	<0.0005
**Adjusted Model[2]**						
Household education						
Up to GCSE	1	(Reference)		1	(Reference)	
A level	0.92	(0.51, 1.65)		0.90	(0.50, 1.59)	
Degree	0.80	(0.45, 1.42)		0.72	(0.38, 1.34)	
Higher degree	0.76	(0.34, 1.71)	0.808	0.64	(0.33, 1.25)	0.432
Deprivation (IMD)[1]	1.26	(1.12, 1.41)	<0.0005	1.27	(1.13, 1.43)	<0.0005
>1 TV	1.76	(1.12, 2.75)	0.014	1.38	(0.88, 2.16)	0.158
Any computers	0.70	(0.41, 1.20)	0.190	0.97	(0.54, 1.75)	0.920
Any tablets	1.90	(1.12, 3.22)	0.017	1.34	(0.46, 3.88)	0.590
Any games consoles	1.02	(0.59, 1.78)	0.943	1.65	(0.95, 2.85)	0.076
Child gender	1.01	(0.73, 1.40)	0.945	1.06	(0.76, 1.49)	0.730
z-BMI score	1.08	(0.92, 1.25)	0.348	0.93	(0.82, 1.05)	0.246
parent multi-SV (mins)[2]	1.26	(1.17, 1.35)	<0.0005	1.21	(1.13, 1.29)	<0.0005
Parental limits on SV	0.62	(0.50, 0.78)	<0.0005	0.72	(0.53, 0.97)	0.033

[1] increase per 1 standard deviation (14.0) in IMD: higher values indicate more deprived areas

[2] Adjusted for child gender, child BMI, presence of TVs, computers, tablets and games consoles in the household, parental screen-viewing and parental limiting of screen-viewing

[3] increase per 30 minutes multi-screen-viewing

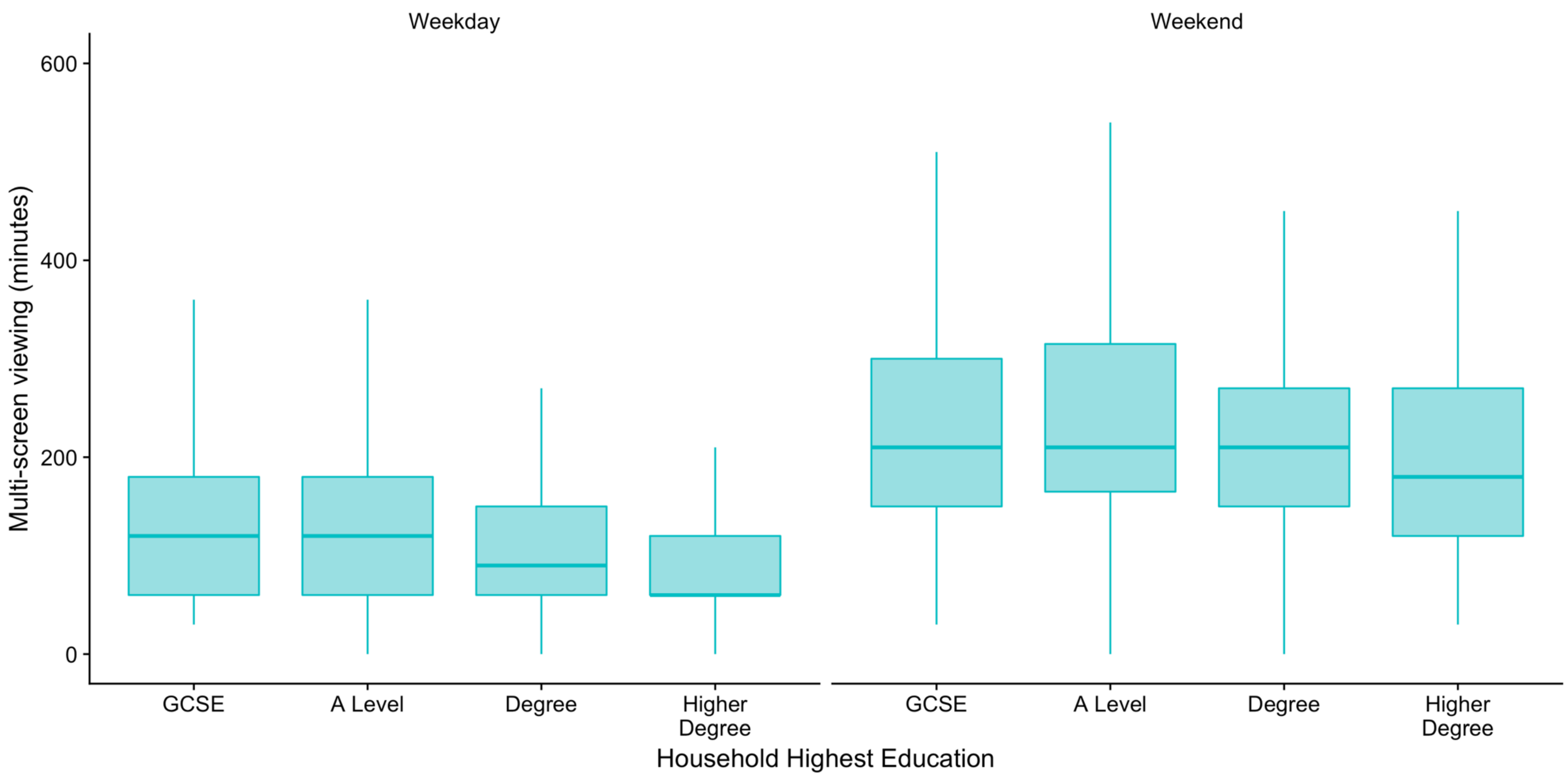

Figure S1: Number of minutes spent multi-screen viewing by household education at age 9, for weekdays (left) and weekends (right).
