# Supplementary material for: Associations between socioeconomic position and changes in children’s screen-viewing between ages 6 and 9: a longitudinal study
Source: BMJ Open. 2019 Dec 10;9(12):e027481. doi: 10.1136/bmjopen-2018-027481 (PMC6924727; doi:10.1136/bmjopen-2018-027481)
Supplement: Author's manuscript [file bmjopen-2018-027481.draft_revisions.pdf]

BMJ Open

BMJ Open is committed to open peer review. As part of this commitment we make the peer review history of every article we publish publicly available.

When an article is published we post the peer reviewers' comments and the authors' responses online. We also post the versions of the paper that were used during peer review. These are the versions that the peer review comments apply to.

The versions of the paper that follow are the versions that were submitted during the peer review process. They are not the versions of record or the final published versions. They should not be cited or distributed as the published version of this manuscript.

BMJ Open is an open access journal and the full, final, typeset and author-corrected version of record of the manuscript is available on our site with no access controls, subscription charges or pay-per-view fees (http://bmjopen.bmj.com).

If you have any questions on BMJ Open's open peer review process please email info.bmjopen@bmj.com

BMJ Open

Socio-economic patterns of changes in children's screen-viewing between ages 6 and 9: a longitudinal study

Journal:	*BMJ Open*
Manuscript ID	bmjopen-2018-027481
Article Type:	Research
Date Submitted by the Author:	24-Oct-2018
Complete List of Authors:	Salway, Ruth; University of Bristol, Centre for Exercise, Nutrition and Health Sciences<br>Emm-Collison, Lydia; University of Bristol, Centre for Exercise, Nutrition and Health Sciences<br>Sebire, Simon; University of Bristol, Centre for Exercise, Nutrition and Health Sciences<br>Thompson, Janice; The University of Birmingham, School of Sport & Exercise Sciences<br>Jago, Russ; University of Bristol, Centre for Exercise, Nutrition and Health Sciences
Keywords:	screen-viewing, PAEDIATRICS, TV, computer games, cohort

SCHOLARONE™
Manuscripts

Socio-economic patterns of changes in children's screen-viewing between ages 6 and 9: a longitudinal study

Ruth Salway[1,*], Lydia Emm-Collison[1], Simon J. Sebire[1], Janice L. Thompson[2] and Russell Jago[1]

[1] Centre for Exercise, Nutrition & Health Sciences, School for Policy Studies, University of Bristol, 8 Priory Road, Bristol, BS8 1TZ, UK.

[2] School of Sport, Exercise and Rehabilitation Sciences, University of Birmingham, Birmingham, B15 2TT, UK

[*] **Corresponding author:** Dr Ruth Salway, Centre for Exercise, Nutrition & Health Sciences, School for Policy Studies, University of Bristol, 8 Priory Road, Bristol, BS8 1TZ, UK.

Email: ruth.salway@bristol.ac.uk; Telephone: +44 (0)117 3310463

Word count: **Manuscript** = 3221 words **Abstract** = 298 words (max 300)

For peer review only - http://bmjopen.bmj.com/site/about/guidelines.xhtml

ABSTRACT

Objectives: To explore socio-economic differences in change in screen-viewing between ages 6 and 9, and how these are related to different media use.

Design: Longitudinal cohort study.

Setting: Children recruited from 57 state-funded primary schools in Southwest England, UK.

Participants: 1299 children at age 5-6, 1223 children at age 8-9, including 685 children at both timepoints.

Outcome measures: Children's total screen-viewing time (parent-reported) and time spent using multiple screen devices simultaneously (multiscreen viewing), for weekdays and weekends.

Methods: Negative binomial regression was used to model associations between total screen-viewing at age 9 and socio-economic variables (highest household education and area deprivation) adjusting for baseline screen-viewing at age 6, child characteristics, parental influences and media devices in the home. Multiscreen viewing was analysed separately.

Results: Weekday screen-viewing time increased by 22% (95% CI: 8% to 39%) for children in households where the highest education was A levels (qualifications at age 18), and by 16% (95% CI: 2% to 32%) where the highest education was a university degree, compared to children in households where the highest education was GCSEs (qualifications at age 16), after adjustment. Increases at weekends were similar. Higher screen-viewing time was associated with the presence of games consoles in the household and more minutes spent multiscreen viewing (especially on weekdays). Lower screen-viewing time was associated with parental limits on screen-viewing in the week, but not at weekends.

For peer review only

Conclusions: Children's screen-viewing patterns differ by parental education with higher levels of viewing among children living in households with lower educational qualifications. These differences are already present at age 6, and continue at age 9, even when accounting for differences in baseline screen-viewing and device ownership. Strategies to manage child sedentary time, and particularly screen-viewing, may need to take account of the socio-economic differences and target strategies to specific groups.

For peer review only

Keywords: screen viewing, TV, computer games, cohort, children

For peer review only - http://bmjopen.bmj.com/site/about/guidelines.xhtml

ARTICLE SUMMARY

Strengths and limitations of this study

Strengths

- Longitudinal study with different types of screen viewing on both weekdays and weekends at two time points.
- Time frame covers an important period of change in child viewing behaviours.
- Data collection period coincided with a period of rapid change in screen-viewing technology which has facilitated the presentation of contemporary data.

Limitations

- We did not explicitly ask parents about tablet use and so have not been able to capture this directly.
- A change in the way education was recorded may result in misclassification in a small number of situations where household composition has changed or where people have obtained additional qualifications.

For peer review only

INTRODUCTION

Sedentary behaviour has been associated with increased risk of obesity and elevated levels of cardio-metabolic risk factors among children and adolescents[1, 2]. There is currently debate as to whether these adverse health effects are independent of physical activity, with some evidence suggesting that any detrimental effects of high levels of sedentary time are attenuated if the individual is very active [3]. However, as large numbers of children do not meet physical activity guidelines[4, 5] there is a need to focus on ways to both increase physical activity and reduce sedentary time, which requires a detailed understanding of both behaviours. While accelerometers can provide accurate measures of sedentary time, they provide no information on the specific behaviour in which children engage and so they provide limited information on which to target behaviour change programmes. Screen-viewing is a very common form of sedentary behaviour among children and young people and higher screen-viewing has been associated with higher body mass index, poorer psychological well-being and poorer academic achievement among children[6-8]. Children with parents who engage in high amounts of screen-viewing are more likely to also engage in high levels of screen-viewing, with different patterns for weekdays and weekend days[9-12]. Screen-viewing has evolved with technology from just TV viewing to include TV viewing, computer games, tablets, mobile phones and multiscreen viewing (in which children use two or more devices at the same time), and become more diverse, for example encompassing on-demand TV and online gaming. Thus, to try and change behaviour there is a need to understand what devices children are using and the types of viewing in which children engage on different days of the week.

Different indicators of socio-economic position, such as education, income and deprivation, measure different, often related aspects, although they are strongly correlated[13, 14], and socio-economic

For peer review only - http://bmjopen.bmj.com/site/about/guidelines.xhtml

position has been associated with several health outcomes among children and young people. For example, in England at the end of primary school (age 11) in 2018 the prevalence of child obesity in the most deprived areas is more than double that of those in the least deprived neighbourhoods (27% vs. 12%)[15]. Recent analysis of data from the International Children's Accelerometer Database has shown that adolescents with university-educated mothers spent 10 more minutes per day being sedentary when compared with adolescents who had mothers who were educated to high school level[16]. Previous studies[17-21] have suggested that there are socio-economic differences in screen-viewing patterns but there is a lack of information on how these differences develop as children age, since most of the evidence is cross-sectional, and a recent review[22] highlighted a need for longitudinal evidence. Thus, there is a need to examine how socio-economic differences in screen-viewing patterns develop during childhood. The aim of this paper was to explore socio-economic differences in the change in screen-viewing between ages 6 and 9, and how these are related to different media use. Developing knowledge in this area will support the creation of targeted behaviour change programmes to reduce screen-viewing.

METHODS

B-PROACT1V is a longitudinal study that aimed to examine the physical activity and sedentary behaviours of primary school children aged 5-11 years, and their parents[4, 23, 24]. The study received ethical approval from the School of Policy Studies Ethics Committee at the University of Bristol, UK, and written parental consent was received for all participants[25]. In Phase 1, all children in Year 1 of primary school (aged 5-6 years) from 57 schools in and around Bristol were invited to participate, with data collected between January 2012 and July 2013. In Phase 2, when the children were in Year 4 (aged

For peer review only - http://bmjopen.bmj.com/site/about/guidelines.xhtml

8-9 years), all schools from Phase 1 were invited to participate, with 47 schools agreeing. All children were eligible regardless of whether they had participated in Phase 1, and data collection took place between March 2015 and July 2016. Data were collected for 1299 children in Year 1 and 1223 children in Year 4, with 685 children included in both phases. Children and at least one parent wore a waist-worn ActiGraph wGT3X-BT accelerometer for five days, including two weekend days. Self-identified 'first' parents completed a questionnaire about personal and family characteristics while 'second' parents completed a shorter questionnaire.

Screen-viewing data

In both years, parents were asked about the number of hours they and their child typically spent engaging in specific screen-viewing behaviours on weekdays and at weekends: TV, computer, games consoles and, in Year 4 only, multiscreen viewing[26]. These were recorded as either 'None' or in hourly categories from '0-1 hours' up to '4 hours or more', and recoded based on midpoints to give the average number of minutes spent in each type of screen-viewing on weekdays and weekends. These were summed to form the average total number of minutes spent on any type of screen-viewing. Where two parents completed questionnaires, parental screen-viewing was taken as the average (approximately 27% of respondents in each year).

Socio-economic data

At age 6, the first parent was asked their highest educational qualification, while at age 9, they were asked the highest education qualification of anyone in the household. We combined these to form the highest household educational qualification recorded at either time with categories 'Up to GCSE/ O level or equivalent' (qualification at age 16), 'A level/NVQ or equivalent' (qualification at age 18), 'Degree/HND or equivalent' and 'Higher Degree (MSc/PhD) or equivalent'. In addition, Index of Multiple Deprivation (IMD) scores, based on the English Indices of Deprivation

For peer review only - http://bmjopen.bmj.com/site/about/guidelines.xhtml

(http://data.gov.uk/dataset/index-of-multiple-deprivation), were assigned to each child based on their reported home postcode, with higher IMD scores indicating a greater level of deprivation. Household education captures individual long-term socio-economic position, such as knowledge, and also provides an indirect indication of income, while IMD captures the socio-economic conditions of the area in which they live.

Other measurements

Child height and weight were measured, and body mass index (BMI) was calculated and converted to an age- and sex-specific standard deviation score based on UK reference curves[27, 28]. Accelerometer data were processed using Kinesoft (v3.3.75; Kinesoft, Saskatchewan, Canada), and were included if participants provided at least three days of valid data (including at least one weekend day), where a valid day was defined as at least 500 minutes of data after excluding intervals of ≥60 minutes of zero counts, allowing up to two minutes of interruptions. Minutes spent in moderate-to-vigorous-intensity physical activity (MVPA) and mean sedentary time per day were derived using population-specific cut points for children[29]. The first parent was asked whether they limited the time their child spent engaging in three different types of screen-viewing (TV, computer and video games) with responses from 1 'Strongly disagree' to 4 'Strongly agree'. The average of these was used to capture parental limits on screen-viewing. We also asked about the number of media devices in the home: TVs, computers (desktop or laptop), tablet computers and games consoles (including handheld consoles).

Statistical Analysis

Descriptive summaries of children's screen-viewing on weekdays and weekends and other participant characteristics were produced for age 6 and age 9. To aid interpretation, the screen-viewing variables are reported in summaries as continuous variables. However, the underlying variables are discrete and so in

For peer review only - http://bmjopen.bmj.com/site/about/guidelines.xhtml

the main analyses negative binomial regression models (similar to Poisson models but suitable for over-dispersed count data) were used to model weekday and weekend screen-viewing separately. We modelled the association between total screen-viewing at age 9 and socio-economic variables (household education and IMD) adjusting for baseline screen-viewing at age 6, child characteristics (sex and BMI z-score), screen-viewing patterns (the percentage of total screen-viewing time spent on non-TV screen-viewing and minutes spent multiscreen viewing), child physical activity (average MVPA and sedentary time), parental influences (parental screen-viewing and limits on screen-viewing score) and presence of media devices in the home (more than one TV and any computers, tablets or games consoles). As it includes more than one form of screen viewing and therefore may duplicate information, we assessed multiscreen viewing as a separate outcome. Robust standard errors were used to account for clustering of children within schools. We explored using a range of different multiple imputation approaches to account for missing data and thus maximise the sample size, but due to the categorical nature of the variables, we encountered problems with convergence. We have therefore not presented any imputed data in the paper.

All model results are presented as screen time ratios (rate ratios), defined as the exponent of the model coefficients. An increase of one unit in a given predictor variable is associated with a multiplicative increase in screen-viewing, holding the other predictor variables in the model constant. Model assumptions were checked via model diagnostics, and all analysis was done in Stata v15.

RESULTS

Missing data ranged between 0-25% (Table S1). Time spent in all types of screen-viewing was higher at weekends than during the week, with time spent watching TV and playing games consoles nearly doubling at weekends (Table 1). Children’s screen-viewing differed between households with differing

For peer review only - http://bmjopen.bmj.com/site/about/guidelines.xhtml

levels of education, with higher screen-viewing in lower qualified households, decreasing as education increased, with 35-45 minutes difference between the highest and lowest household education qualifications (Figure 1). The percentage of sedentary time spent screen-viewing decreased with education.

Except for weekday TV, which remains about the same, time spent in all types of screen-viewing was higher at age 9 than at age 6, with total screen-viewing nearly 20 minutes higher in the week and around 50 minutes higher at weekends (Tables 1 and 2). Again, screen-viewing was higher among

Table 1 – Characteristics at age 6

	Up to GCSE or equiv	A Levels or equiv	Degree or equiv	Higher Degree or equiv	All
	Mean (sd) or %	Mean (sd) or %	Mean (sd) or %	Mean (sd) or %	Mean (sd) or %
WEEKDAY	(n=201)	(n=326)	(n=430)	(n=174)	(n=1131)
Total SV (mins)	120 (74)	111 (61)	93 (60)	85 (70)	101 (66)
TV (mins)	72 (53)	69 (45)	60 (41)	53 (44)	64 (46)
Computer (mins)	25 (28)	23 (27)	20 (22)	23 (33)	22 (26)
Games consoles (mins)	23 (30)	18 (20)	14 (25)	10 (20)	16 (24)
% of sed time spent SV	34%	30%	25%	23%	28%
% of SV on TV	61%	63%	67%	64%	64%
% of SV on computer	21%	21%	21%	27%	22%
% of SV on console	17%	16%	12%	9%	14%
WEEKEND	(n=201)	(n=326)	(n=430)	(n=174)	(n=1131)
Total SV (mins)	196 (118)	193 (107)	169 (94)	150 (102)	178 (105)
TV (mins)	116 (78)	120 (72)	110 (69)	89 (68)	111 (72)
Computer (mins)	38 (44)	34 (38)	32 (33)	37 (39)	34 (37)
Games consoles (mins)	43 (55)	40 (45)	28 (39)	24 (39)	33 (45)
% of sed time spent SV	58%	59|%	51%	45%	53%
% of SV on TV	56%	58%	60%	53%	58%
% of SV on computer	22%	20%	25%	34%	24%
% of SV on console	22%	21%	15%	14%	18%
IMD	19.9 (15.5)	16.6 (13.8)	12.3 (10.5)	10.3 (7.4)	14.6 (12.6)
Sex	46%	47%	51%	48%	48%

For peer review only - http://bmjopen.bmj.com/site/about/guidelines.xhtml

BMI z-score	0.47 (1.03)	0.18 (0.88)	0.16 (0.88)	0.24 (0.94)	0.23 (0.92)
MVPA: weekday	69.6 (21.4)	68.3 (19.9)	67.3 (21.7)	66.4 (18.7)	67.8 (20.7)
Sed: weekday	358.0 (56.1)	366.6 (56.7)	377.6 (64.8)	382.1 (52.0)	371.9 (59.7)
MVPA: weekend	66.7 (29.5)	66.2 (29.1)	65.1 (26.0)	67.9 (26.7)	66.1 (27.5)
Sed: weekend	336.0 (94.3)	337.9 (74.3)	342.5 (77.3)	341.4 (59.9)	340.0 (76.8)
Parent SV: weekday (mins)	231 (142)	231 (134)	223 (115)	202 (118)	223 (126)
Parent SV: weekend (mins)	254 (147)	249 (125)	233 (106)	209 (118)	237 (122)
Parental limits on SV	3.2 (0.6)	3.3 (0.6)	3.4 (0.6)	3.5 (0.6)	3.3 (0.6)
No. TVs	2.7 (1.3)	2.6 (1.3)	2.2 (1.1)	1.7 (1.1)	2.3 (1.2)
No. Computers	1.9 (1.4)	2.1 (1.3)	2.4 (1.4)	2.5 (1.5)	2.3 (1.4)
No. Tablets	0.4 (0.7)	0.5 (0.7)	0.5 (0.7)	0.5 (0.7)	0.5 (0.7)
No. games consoles	2.8 (1.7)	2.8 (1.7)	2.0 (1.5)	1.3 (1.3)	2.3 (1.7)

For peer review only

For peer review only - http://bmjopen.bmj.com/site/about/guidelines.xhtml

Table 2 - Characteristics at age 9

	Up to GCSE 0r equiv	A Levels or equiv	Degree or equiv	Higher Degree or equiv	All
	Mean (sd) or %	Mean (sd) or %	Mean (sd) or %	Mean (sd) or %	Mean (sd) or %
WEEKDAY	(n=166)	(n=328)	(n=415)	(n=168)	(n=1077)
Total SV (mins)	139 (94)	133 (78)	113 (75)	88 (59)	118 (78)
TV (mins)	75 (55)	72 (49)	63 (46)	48 (38)	65 (48)
Computer (mins)	37 (46)	34 (37)	31 (37)	29 (31)	32 (37)
Games consoles (mins)	28 (44)	26 (34)	19 (30)	12 (24)	21 (33)
Multi-screen viewing (mins)	29 (46)	24 (40)	16 (33)	11 (25)	19 (37)
% of sed time spent SV	30%	30%	25%	19%	26%
% of SV on TV	59%	56%	58%	55%	57%
% of SV on computer	24%	25%	27%	34%	27%
% of SV on console	17%	19%	15%	11%	16%
WEEKEND	(n=166)	(n=328)	(n=415)	(n=168)	(n=1077)
Total SV (mins)	244 (134)	245 (121)	227 (118)	200 (118)	230 (122)
TV (mins)	127 (72)	126 (66)	124 (63)	104 (62)	122 (65)
Computer (mins)	59 (66)	58 (64)	53 (60)	57 (54)	56 (61)
Games consoles (mins)	58 (68)	62 (63)	49 (61)	39 (54)	52 (62)
Multi-screen viewing (mins)	53 (73)	48 (66)	33 (60)	23 (45)	39 (63)
% of sed time spent SV	59%	60%	55%	46%	55%
% of SV on TV	56%	55%	58%	55%	56%
% of SV on computer	23%	21%	23%	29%	23%
% of SV on console	21%	24%	19%	16%	20%
IMD	20.5 (15.9)	17.6 (14.9)	12.9 (11.7)	12.1 (10.3)	15.4 (13.6)
Sex	56%	51%	57%	52%	54%
BMI z-score	0.44 (1.15)	0.38 (1.04)	0.33 (1.06)	0.28 (1.02)	0.31 (1.06)
MVPA: weekday	60.8 (23.2)	63.2 (24.4)	62.1 (21.7)	63.0 (19.8)	62.4 (22.5)
Sed: weekday	441.1 (69.2)	444.3 (64.4)	449.8 (62.2)	458.4 (62.3)	448.3 (64.0)
MVPA: weekend	61.1 (33.5)	62.6 (33.2)	61.2 (31.9)	60.3 (27.5)	61.5 (31.8)
Sed: weekend	387.5 (92.5)	396.1 (84.2)	410.8 (82.8)	413.0 (80.8)	403.7 (84.6)
Parent SV: weekday (mins)	247 (134)	245 (128)	247 (134)	255 (145)	248 (134)
Parent SV: weekend (mins)	279 (156)	287 (135)	261 (129)	252 (143)	269 (138)
Parental limits on SV	3.1 (0.7)	3.2 (0.6)	3.4 (0.6)	3.4 (0.5)	3.3 (0.6)
No. TVs	2.9 (1.3)	3.1 (1.4)	2.4 (1.3)	1.8 (1.1)	2.6 (1.4)
No. Computers	1.6 (1.2)	1.7 (1.1)	2.0 (1.2)	2.2 (1.1)	1.9 (1.2)

For peer review only

No. Tablets	2.1 (1.2)	2.3 (1.3)	2.1 (1.2)	2.0 (1.2)	2.2 (1.3)
No. games consoles	2.6 (2.1)	2.8 (1.8)	2.2 (1.6)	1.7 (1.6)	2.3 (1.8)

For peer review only

For peer review only - http://bmjopen.bmj.com/site/about/guidelines.xhtml

households with lower qualifications, with a 45-50 minute difference between the highest and lowest (Figure 1). Time spent engaged in multiscreen viewing was also higher for households with lower levels of qualification, with children in households where GCSEs were the highest qualification spending over twice as much time in multiscreen viewing as those where the highest qualification was a postgraduate degree, on both weekdays and weekends.

There were differences in the number and types of media devices in the home by household education. Households where GCSE and A Levels were the highest qualification had more TVs and games consoles in the house, while Higher Degree households had more computers. There were small increases in most devices between age 6 and age 9. The average number of tablet devices was the same across all levels of household education, but increased fourfold to an average of 2.2 per household between the two assessment periods. Parental screen-viewing differed with household education by similar amounts to their children, again with higher screen-viewing among those with lower level qualifications. Parental screen-viewing was also higher at the second time point, especially among the Higher Degree educated households, and differences with education were smaller.

Table 3 reports the negative binomial model for total screen-viewing, in terms of ratios for screen time, which should be interpreted as multiplicative factors for a one-unit increase. For example, a factor of 1.22 for A Level household education (Table 3, weekday) means that screen-viewing time increased by 22% for children in households where A Levels were the highest qualification, compared to children in households where the highest qualifications were GCSEs. Differences in screen-viewing by household education were present when adjusting for baseline screen-viewing, child characteristics, parental influences and media devices in the home. The change in screen-viewing between ages 6 and 9 was

For peer review only - http://bmjopen.bmj.com/site/about/guidelines.xhtml

highest in households with A Level and university degree qualifications (increasing by 21-22% and 16-18% respectively), compared to households with GCSE qualifications (Figure 2, left). There was no association with IMD once household education was taken into account. The presence of games consoles and higher multiscreen viewing were both associated with an increase in screen-viewing of 25-27%. There were weaker associations between screen-viewing and baseline screen-viewing at age 6, parental screen-viewing and proportion of non-TV screen-viewing. Lower screen-viewing was associated with parental limits on screen-viewing in the week, but not at weekends, and with the presence of tablet devices in the home. There was no association between children's screen-viewing and physical activity.

Multiscreen viewing was associated with different factors to overall screen-viewing (Table S2). There was no association between multiscreen viewing and households with different education levels. However, there was a strong association with IMD, especially on weekdays, with children's multiscreen viewing higher in families living in more deprived areas (Figure 2, right). Higher amounts of multiscreen viewing were associated with higher levels of TV and computer use and the presence of tablet devices, and lower levels of multiscreen viewing were associated with increased parental limits on screen-viewing. We also saw an association between lower weekend multiscreen viewing and both higher MVPA and lower sedentary time.

For peer review only - http://bmjopen.bmj.com/site/about/guidelines.xhtml

Table 3 – Negative binomial model for total screen-viewing (SV) (mins)

	WEEKDAY N=431			WEEKEND N=396		
	Ratio[1]	95% CI	p	Ratio[1]	95% CI	p
Up to GCSE	1.00		0.001	1.00		0.001
A level	1.22	(1.08, 1.39)		1.21	(1.07, 1.36)	
Degree	1.16	(1.02, 1.32)		1.18	(1.03, 1.34)	
Higher degree	0.93	(0.79, 1.09)		1.01	(0.88, 1.16)	
IMD [2]	1.00	(0.95, 1.06)	0.868	0.98	(0.94, 1.03)	0.466
Baseline Y1 SV [3]	1.06	(1.04, 1.09)	<0.0005	1.04	(1.03, 1.05)	<0.0005
Sex	1.04	(0.97, 1.13)	0.275	0.95	(0.87, 1.03)	0.177
z-BMI score	1.01	(0.97, 1.06)	0.668	1.02	(0.98, 1.06)	0.340
% of SV on non-TV[4]	1.02	(1.01, 1.03)	<0.0005	1.02	(1.02, 1.03)	<0.0005
Multi-SV (mins) [3]	1.12	(1.07, 1.17)	<0.0005	1.04	(1.02, 1.06)	<0.0005
Average MVPA (mins) [3]	0.98	(0.93, 1.04)	0.576	0.99	(0.96, 1.02)	0.583
Average sed time (mins) [3]	0.99	(0.97, 1.01)	0.468	1.00	(0.98, 1.01)	0.731
Parent SV (mins)[3]	1.03	(1.02, 1.04)	<0.0005	1.04	(1.03, 1.05)	<0.0005
Parental limits on SV	0.85	(0.79, 0.93)	<0.0005	0.97	(0.90, 1.04)	0.389
>1 TV	1.07	(0.97, 1.17)	0.174	1.05	(0.95, 1.17)	0.338
Any computers	1.23	(0.95, 1.59)	0.121	1.03	(0.91, 1.17)	0.638
Any tablets	0.74	(0.63, 0.88)	0.001	0.78	(0.64, 0.96)	0.022
Any games consoles	1.27	(1.11, 1.44)	<0.0005	1.25	(1.07, 1.47)	0.006

[1] An increase of one unit in a given predictor variable is associated with a multiplicative increase in screen-viewing: e.g., every 30 minutes spent in screen-viewing at age 6 was associated with an increase in age 9 screen-viewing of 6% on weekdays and 4% on weekends

[2] increase per 1 standard deviation (14.0) in IMD

[3] increase per 30 minutes screen-viewing

[4] increase for 10 percentage point change in screen-viewing from TV to non-TV screen-viewing

For peer review only - http://bmjopen.bmj.com/site/about/guidelines.xhtml

DISCUSSION

The data presented in this paper have shown an association between children's screen-viewing and highest household education level, with higher screen-viewing in lower socio-economic groups. These differences were already present at age 6 and continued to be evident at age 9. Except for TV viewing, the time children spent engaged in screen-viewing increased in all households, regardless of education, between ages 6 and 9 on both weekdays and weekends. Adjusting for baseline screen-viewing at age 6, screen-viewing increased most over the time period for children in households where the highest qualifications were A Levels or a university degree. In A-level educated households, this reflected a "catching-up" with the level of screen-viewing seen in GCSE-educated households. These findings imply socio-economic differences in the type and amount of sedentary time and screen-viewing, and the way in which screen-viewing patterns develop. These differences were related to household education rather than area-level deprivation, suggesting that it is long-term socio-economic aspects such as parental knowledge, ability to engage and communicate with services, and possibly income that are important, rather than the socio-economic conditions of the area, such as availability of local resources. In contrast, multiscreen viewing is associated with area deprivation rather than education and so could represent a proxy for short-term current income or possibly a neighbourhood effect of what is considered to be 'typical' screen-viewing behaviour. As such, strategies to manage child sedentary time, and particularly different types of screen-viewing, may need to take account of the socio-economic differences and target strategies to specific groups.

Participants in households with more devices engaged in more screen-viewing. For example, a child with one or more games consoles in the home engaged in 20% more screen-viewing on weekdays, the equivalent of 144 minutes per day compared to 120 minutes per day for a child with no games consoles.

For peer review only - http://bmjopen.bmj.com/site/about/guidelines.xhtml

The number of media devices in the home differed by household education, with more TVs and games consoles in households with GCSE and A level qualifications, and more computers in households with a university or higher degree. While the number of devices changed between the two assessment points, socio-economic differences were small and did not account for the observed differences in total screen-viewing between household education. Findings are therefore consistent with previous studies that have a shown a link between the number of media devices and screen-viewing behaviours[12, 24]. However, we extend those findings to show that while access to devices may contribute to baseline screen-viewing, it does not entirely explain socio-economic differences in the increase in screen-viewing between ages 6 and 9.

There were weak associations between parent and child screen-viewing, with every 30 minutes of parental screen-viewing associated with a 2% increase in child screen-viewing. This association was stronger (~6%) for TV viewing which, consistent with previous studies[12], may reflect parents and children watching TV together. It is important to note that parental limits on screen-time appeared to only be associated with weekday TV and multiscreen viewing. Interestingly, analysis of interviews conducted with a sub-sample of the parents in the study showed that many parents are uneasy about managing non-TV screen-time and feel that they struggle to keep up with rapid technological change[30]. Collectively, these findings may suggest that parental influence on screen-viewing that is not TV viewing is likely to be limited and that there is a need to help parents to identify effective ways to manage constantly adapting forms of viewing.

Tablet ownership did not vary with household education, but increased greatly overall between ages 6 and 9. A limitation of this project is that we did not assess tablet use as a specific behaviour, although we found tablet ownership strongly associated with increased multiscreen viewing. The widespread ownership of tablets is particularly important for understanding the complexity of screen-viewing, as

For peer review only - http://bmjopen.bmj.com/site/about/guidelines.xhtml

watching TV, playing games and using the internet can all be done via a tablet and, as such, identifying the behaviour engaged in while using a tablet is challenging. Thus, it may be that in the future there is a specific need to assess tablet time. A challenge, however, would be how to use tablet data to help parents work with their children to manage overall screen-time, as parents have different views about the benefits and challenges of different forms of screen-viewing. For example, TV viewing is often considered to be family time, while tablet and game console use can be solitary and isolate child from the family [31]. Thus, monitoring tablet use which captures multiple different activities may not be that helpful on its own and it would be more useful to differentiate the various ways in which tablets are used. One way that this could be achieved is via applications that monitor and limit the time spent on games and other non-educational activities. Parents and children could then work together to agree targets for overall and game time on tablets, and the information acquired through the application could be used as a feedback and monitoring device.

The major strengths of this study are the information on a variety of different types of screen-viewing at two time points which span an important period of change in child viewing behaviours. The data collection period (2012-16) coincided with a period of rapid change in screen-viewing technology which has facilitated the presentation of contemporary data. There are, however, several limitations that need to be considered. Firstly, we did not explicitly ask parents about tablet use. As data collection covers a time when screen use is changing rapidly, (for example, global tablet sales increased from 116 million in 2012[32] to 207 million in 2015[33]) this is an important aspect we have not been able to capture directly. Secondly, the differences in the education variable between Year 1 (first parent's education) and Year 4 (household education) may result in inaccuracies in situations where household composition has changed or where people have obtained additional qualifications between the two time points, although we believe that the number of these cases is likely to be small. Finally, as parental screen-

For peer review only - http://bmjopen.bmj.com/site/about/guidelines.xhtml

viewing increased between 2012 and 2015 this may indicate a general change in viewing patterns, and the increases reported between the two age periods could reflect secular changes as opposed to age-related differences. As such, it would be important to identify if comparable patterns are evident in other datasets.

Conclusion

Children's screen-viewing patterns differ by parental education with higher levels of viewing among children living in households with lower levels of education. These differences are already present at age 6, and continue at age 9, even when accounting for differences in baseline screen-viewing and device ownership. Socio-economic differences narrow with age as children in households with higher qualifications gain greater access to screen-viewing devices. Strategies to manage child sedentary time, and particularly screen-viewing, may need to take account of the socio-economic differences and target strategies to specific groups.

ACKNOWLEDGEMENTS

We would like to thank all of the families and schools that have taken part in the B-PROACT1V project. We would also like to thank all current and previous members of the research team who are not authors on this paper.

COMPETING INTERESTS

We have no competing interest to report.

FUNDING

For peer review only - http://bmjopen.bmj.com/site/about/guidelines.xhtml

This work was supported by the British Heart Foundation (ref PG/11/51/28986 and SP 14/4/31123). The funder had no involvement in data analysis, data interpretation or writing of the paper.

AUTHOR CONTRIBUTIONS

RJ, SJS and JLT were involved in the design of this study and in seeking funding for it. The paper was conceived by RJ and RS, and RS performed all analyses. RS and RJ wrote the first draft of the paper and RJ coordinated contributions from other authors. All authors made critical comments on drafts of the paper.

DATA SHARING STATEMENT

The datasets generated during the current study are not publicly available due as the project is ongoing and data are not ready for archiving. We will consider reasonable requests for access to the data once the project is complete in 2019.

REFERENCES

1. Ekelund U, Brage S, Froberg K, Harro M, Anderssen SA, Sardinha LB, et al. TV viewing and physical activity are independently associated with metabolic risk in children: the European Youth Heart Study. PLoS Med. 2006;3:e488.
2. Ekelund U, Luan J, Sherar LB, Esliger DW, Griew P, Cooper A, et al. Moderate to vigorous physical activity and sedentary time and cardiometabolic risk factors in children and adolescents. JAMA. 2012;307:704-12. doi: 10.1001/jama.2012.156.
3. Ekelund U, Steene-Johannessen J, Brown WJ, Fagerland MW, Owen N, Powell KE, et al. Does physical activity attenuate, or even eliminate, the detrimental association of sitting time with mortality? A harmonised meta-analysis of data from more than 1 million men and women. Lancet. 2016;388:1302-10. doi: 10.1016/S0140-6736(16)30370-1.
4. Jago R, Solomon-Moore E, Macdonald-Wallis C, Sebire SJ, Thompson JL, Lawlor DA. Change in children's physical activity and sedentary time between Year 1 and Year 4 of primary school in the B-PROACT1V cohort. Int J Behav Nutr Phys Act. 2017;14:33. doi: 10.1186/s12966-017-0492-0.
5. Cooper AR, Goodman A, Page AS, Sherar LB, Esliger DW, van Sluijs EM, et al. Objectively measured physical activity and sedentary time in youth: the International children's accelerometry database (ICAD). Int J Behav Nutr Phys Act. 2015;12:113. doi: 10.1186/s12966-015-0274-5.
6. Corder K, Atkin AJ, Bamber DJ, Brage S, Dunn VJ, Ekelund U, et al. Revising on the run or studying on the sofa: prospective associations between physical activity, sedentary behaviour, and exam

For peer review only - http://bmjopen.bmj.com/site/about/guidelines.xhtml

results in British adolescents. Int J Behav Nutr Phys Act. 2015;12:106. doi: 10.1186/s12966-015-0269-2.
7. Page AS, Cooper AR, Griew P, Jago R. Children's Screen Viewing is Related to Psychological Difficulties Irrespective of Physical Activity. Pediatrics. 2010;126:e1011-7. http://www.ncbi.nlm.nih.gov/entrez/query.fcgi?cmd=Retrieve&db=PubMed&dopt=Citation&list_uids=20937661
8. Jago R, Baranowski T, Baranowski J, Thompson D, Greaves K. BMI from 3-6 y of age is predicted by TV viewing and physical activity, not diet. Int J Obes Relat Metab Disord. 2005;29:557 - 64.
9. Jago R, Davison K, Thompson JL, Page AS, Brockman R, Fox KR. Parental sedentary restriction, maternal parenting style and TV viewing among 10-11 year olds Pediatrics. 2011;128:e572-e8.
10. Jago R, Page A, Froberg K, Sardinha L, Klasson-Heggebo L, Andersen L. Screen-viewing and the home TV environment: The European Youth Heart Study. Prev Med. 2008;47:525 - 9.
11. Jago R, Sebire SJ, Edwards MJ, Thompson JL. Parental TV viewing, parental self-efficacy, media equipment and TV viewing among preschool children. Eur J Pediatr. 2013;172:1543-5. doi: 10.1007/s00431-013-2077-5.
12. Jago R, Stamatakis E, Gama A, Carvalhal IM, Nogueira H, Rosado V, et al. Parent and child screen-viewing time and home media environment. American journal of preventive medicine. 2012;43:150-8. doi: 10.1016/j.amepre.2012.04.012.
13. Galobardes B, Shaw M, Lawlor DA, Lynch JW, Davey Smith G. Indicators of socioeconomic position (part 1). J Epidemiol Community Health. 2006;60:7-12. doi: 10.1136/jech.2004.023531.
14. Galobardes B, Shaw M, Lawlor DA, Lynch JW, Davey Smith G. Indicators of socioeconomic position (part 2). J Epidemiol Community Health. 2006;60:95-101. doi: 10.1136/jech.2004.028092.
15. NHS Digital. National Child Measurement Programme, England 2017-2018 school year. UK: 2018.
16. Sherar LB, Griffin TP, Ekelund U, Cooper AR, Esliger DW, van Sluijs EM, et al. Association between maternal education and objectively measured physical activity and sedentary time in adolescents. J Epidemiol Community Health. 2016;70:541-8. doi: 10.1136/jech-2015-205763.
17. Drenowatz C, Eisenmann JC, Pfeiffer KA, Welk G, Heelan K, Gentile D, et al. Influence of socio-economic status on habitual physical activity and sedentary behavior in 8- to 11-year old children. BMC Public Health. 2010;10:214.
18. Fletcher EN, Whitaker RC, Marino AJ, Anderson SE. Screen Time at Home and School among Low-Income Children Attending Head Start. Child Indic Res. 2014;7:421-36. doi: 10.1007/s12187-013-9212-8.
19. Yang-Huang J, van Grieken A, Moll HA, Jaddoe VWV, Wijtzes AI, Raat H. Socioeconomic differences in children's television viewing trajectory: A population-based prospective cohort study. PloS one. 2017;12:e0188363. doi: 10.1371/journal.pone.0188363.
20. Tandon P, Chuan Zhou C, Sallis J, Cain K, Frank L, Saelens B. Home environment relationships with children's physical activity, sedentary time, and screen time by socioeconomic status. Int J Behav Nutr Phys Act. 2012;9:88.
21. Moraeus L, Lissner L, Olsson L, Sjoberg A. Age and time effects on children's lifestyle and overweight in Sweden. BMC Public Health. 2015;15:355. doi: 10.1186/s12889-015-1635-3.
22. Gebremariam MK, Altenburg TM, Lakerveld J, Andersen LF, Stronks K, Chinapaw MJ, et al. Associations between socioeconomic position and correlates of sedentary behaviour among youth: a systematic review. Obes Rev. 2015;16:988-1000. doi: 10.1111/obr.12314.

For peer review only - http://bmjopen.bmj.com/site/about/guidelines.xhtml

23. Jago R, Sebire SJ, Wood L, Pool L, Zahra J, Thompson JL, et al. Associations between objectively assessed child and parental physical activity: a cross-sectional study of families with 5-6 year old children. BMC public health. 2014;14:655. doi: 10.1186/1471-2458-14-655.
24. Jago R, Thompson JL, Sebire SJ, Wood L, Pool L, Zahra J, et al. Cross-sectional associations between the screen-time of parents and young children: differences by parent and child gender and day of the week. Int J Behav Nutr Phys Act. 2014;11:54. doi: 10.1186/1479-5868-11-54.
25. Jago R, Bailey R. Ethics and paediatric exercise science: Issues and making a submission to a local ethics and research committee. Journal of Sport Sciences. 2001;19:527-35.
26. Jago R, Sebire SJ, Gorely T, Cillero IH, Biddle SJ. "I'm on it 24/7 at the moment": a qualitative examination of multi-screen viewing behaviours among UK 10-11 year olds. Int J Behav Nutr Phys Act. 2011;8:85. doi: 10.1186/1479-5868-8-85.
27. Cole TJ, Freeman JV, Preece MA. Body mass index reference curves for the UK, 1990. Arch Dis Child. 1995;73:25-9.
28. Cole TJ, Bellizzi MC, Flegal KM, H. DW. Establishing a standard definition for child overweight and obesity worldwide: international survey. BMJ Open. 2000;320:1240-3.
29. Evenson KR, Catellier DJ, Gill K, Ondrak KS, McMurray RG. Calibration of two objective measures of physical activity for children. Journal of sports sciences. 2008;26:1557-65. doi: 10.1080/02640410802334196.
30. Jago R, Solomon-Moore E, Toumpakari Z, Lawlor DA, Thompson JL, Sebire SJ. Parents' perspectives of change in child physical activity & screen-viewing between Y1 (5-6) & Y4 (8-9) of primary school: implications for behaviour change. BMC public health. 2018;18:520. doi: 10.1186/s12889-018-5445-2.
31. Thompson JL, Sebire SJ, Kesten JM, Zahra J, Edwards M, Solomon-Moore E, et al. How parents perceive screen viewing in their 5-6 year old child within the context of their own screen viewing time: a mixed-methods study. BMC public health. 2017;17:471. doi: 10.1186/s12889-017-4394-5.
32. Gartner. Gartner Says Worldwide Tablet Sales Grew 68 Percent in 2013, With Android Capturing 62 Percent of the Market 2014 [cited 2018 18 Sep]. Available from: https://www.gartner.com/newsroom/id/2674215.
33. IDC. Worldwide Shipments of Slate Tablets Continue to Decline While Detachable Tablets Climb to New High, According to IDC 2016 [cited 2018 18 Sep]. Available from: https://web.archive.org/web/20160204014026/http://www.idc.com/getdoc.jsp?containerId=prUS40990116.

For peer review only

For peer review only - http://bmjopen.bmj.com/site/about/guidelines.xhtml

FIGURE LEGENDS AND KEYS

Figure 1: Total minutes of screen-viewing by highest household education at ages 6 and 9.

Figure 2: Modelled total minutes of screen-viewing (left) and multiscreen viewing (right) by socioeconomic position.

Models are adjusted for baseline screen-viewing at age 6, child characteristics, child physical activity.

Screen viewing additionally adjusted for screen-viewing types at age 9.

Higher Index of Multiple deprivation scores indicate more deprived households

For peer review only

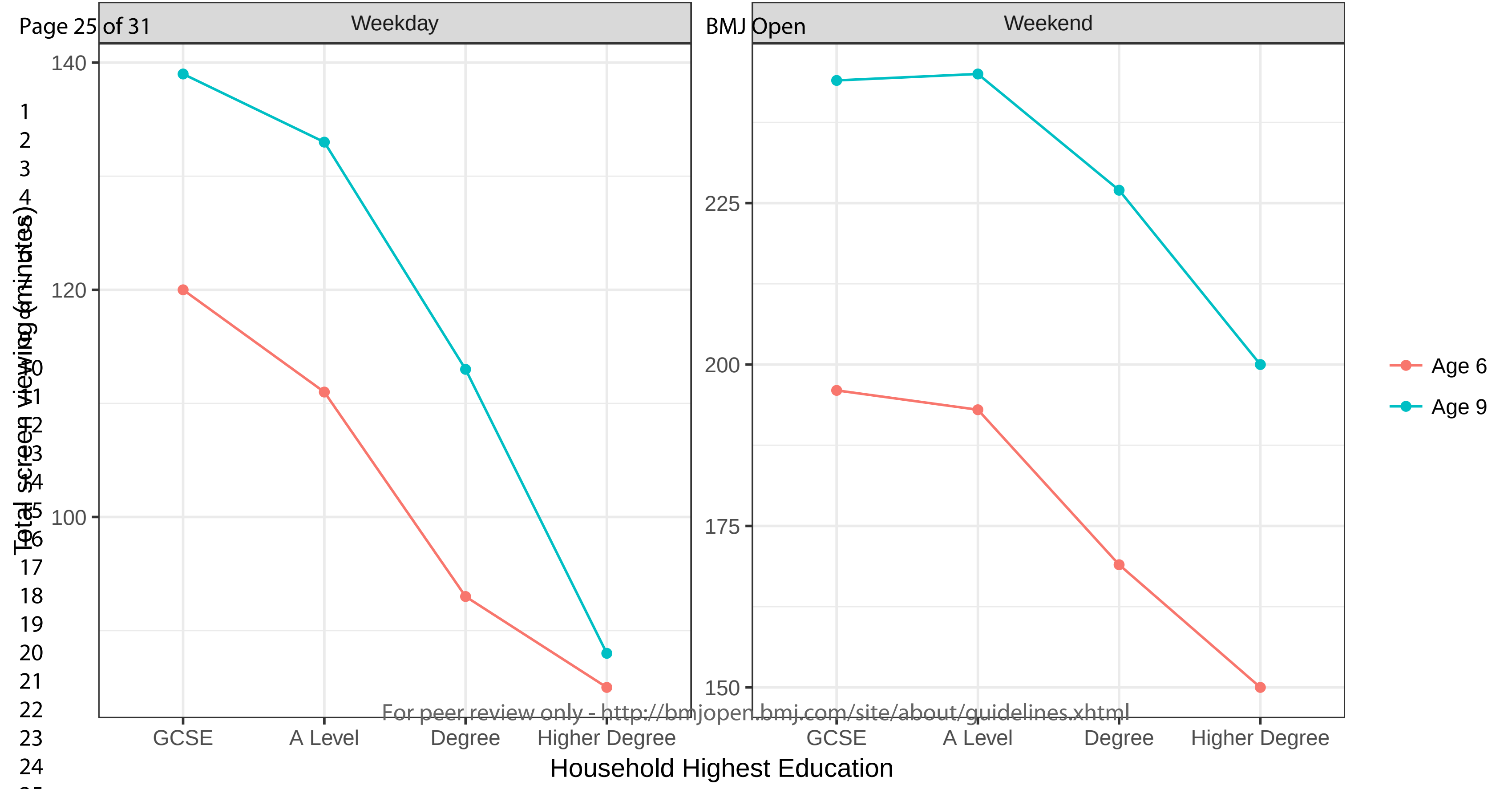
Weekday
Weekend
Total screen viewing (minutes)
140
120
100
225
200
175
150
GCSE
A Level
Degree
Higher Degree
Household Highest Education
Age 6
Age 9

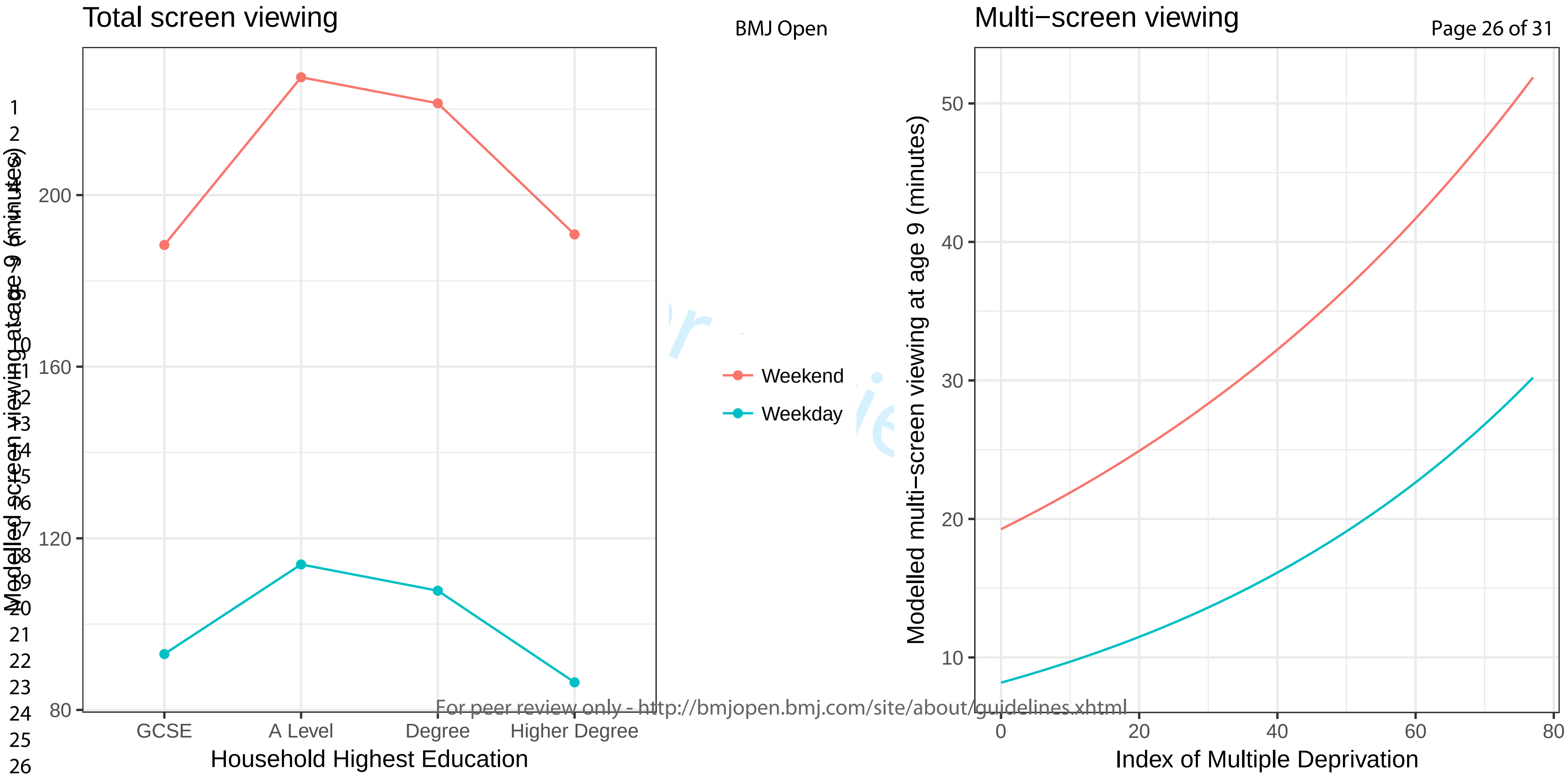

For peer review only - http://bmjopen.bmj.com/site/about/guidelines.xhtml

SUPPLEMENTARY MATERIAL

Socio-economic patterns of changes in children's screen-viewing between ages 6 and 9: a longitudinal study

Ruth Salway, Lydia Emm-Collison, Simon J. Sebire, Janice L. Thompson and Russell Jago

For peer review only

List of Supplementary Tables

Table S1 - Missing values
Table S2 - Negative binomial model for multiscreen viewing (mins)

Table S1 – Missing values

	Age 6		Age 9		Data at both time points Age 6		Age 9	
	N	%	N	%	N	%	N	%
Total	1299		1223		685		685	
Household education	168	13%	146	12%	30	4%	30	4%
**WEEKDAY**								
Total SV (mins)	230	18%	243	20%	100	15%	122	18%
TV (mins)	221	17%	242	20%	95	14%	121	18%
Computer (mins)	223	17%	241	20%	98	14%	120	18%
Games consoles (mins)	225	17%	242	20%	96	14%	121	18%
Multi-SV (mins)			241	20%			120	18%
MVPA	212	16%	146	12%	97	14%	78	11%
Sedentary time	212	16%	164	13%	97	14%	88	13%
Parent SV	207	16%	225	18%	97	14%	115	17%
**WEEKEND**								
Total SV (mins)	234	18%	244	20%	104	15%	123	18%
TV (mins)	227	17%	242	20%	99	14%	121	18%
Computer (mins)	224	17%	242	20%	98	14%	121	18%
Games consoles (mins)	225	17%	242	20%	98	14%	121	18%
Multi-SV (mins)			241	20%			120	18%
MVPA	319	25%	263	22%	145	21%	137	20%
Sedentary time	319	25%	281	23%	145	21%	147	21%
Parent SV	215	17%	227	19%	98	14%	117	17%
IMD	127	10%	19	2%	58	8%	12	2%
Sex	0	0%	0	0%	0	0%	0	0%
BMI z-score	275	21%	255	21%	115	17%	126	18%
Parental limits on SV	270	20%	252	21%	142	21%	126	18%
No. TVs	264	20%	240	20%	131	19%	121	18%
No. Computers	279	21%	240	20%	134	20%	121	18%
No. Tablets	275	21%	241	20%	134	20%	121	18%
No. games consoles	280	22%	241	20%	138	20%	121	18%

For peer review only

Table S2 – Negative binomial model for multiscreen viewing (mins)

	WEEKDAY	N=435		WEEKEND	N=396	
	Ratio[1]	95% CI	p	Ratio[1]	95% CI	p
Up to GCSE	1.00		0.613	1.00		0.381
A level	0.92	(0.52, 1.64)		0.68	(0.38, 1.19)	
Degree	0.79	(0.44, 1.43)		0.55	(0.31, 0.95)	
Higher degree	0.90	(0.46, 1.78)		0.72	(0.37, 1.39)	
IMD [2]	1.27	(1.13, 1.43)	<0.0005	1.20	(1.04, 1.38)	0.012
Baseline Y1 SV [3]	1.05	(0.97, 1.14)	0.230	1.06	(1.00, 1.14)	0.062
Sex	0.79	(0.58, 1.06)	0.118	0.94	(0.66, 1.35)	0.746
z-BMI score	1.15	(1.00, 1.32)	0.043	0.95	(0.85, 1.07)	0.438
TV (mins) [3]	1.20	(1.06, 1.37)	0.005	1.12	(1.04, 1.21)	0.002
Computer (mins) [3]	1.24	(1.12, 1.36)	<0.0005	1.10	(1.04, 1.18)	0.003
Games (mins) [3]	1.14	(0.99, 1.32)	0.067	1.07	(0.95, 1.19)	0.275
Average MVPA (mins) [3]	0.85	(0.66, 1.08)	0.183	0.86	(0.74, 0.99)	0.038
Average sed time (mins) [3]	1.07	(0.99, 1.16)	0.074	0.93	(0.88, 0.98)	0.006
Parent SV (mins) [3]	1.03	(0.99, 1.06)	0.135	1.04	(1.00, 1.08)	0.027
Parental limits on SV	0.74	(0.55, 0.98)	0.039	0.69	(0.51, 0.93)	0.016
>1 TV	1.82	(1.04, 3.19)	0.036	1.48	(0.94, 2.32)	0.092
1+ computers	0.52	(0.33, 0.80)	0.003	0.94	(0.43, 2.06)	0.870
1+ tablets	2.84	(1.50, 5.38)	0.001	2.49	(0.59, 10.52)	0.214
1+ games consoles	0.88	(0.46, 1.55)	0.594	1.25	(0.75, 2.09)	0.395

[1] An increase of one unit in a given predictor variable is associated with a multiplicative increase in screen-viewing: e.g., every 30 minutes spent in TV viewing at age 6 was associated with an increase in age 9 multi-screen viewing of 20% on weekdays and 16% on weekends

[2] increase per 1 standard deviation (14.0) in IMD

[3] increase per 30 minutes screen-viewing

For peer review only

STROBE 2007 (v4) Statement—Checklist of items that should be included in reports of *cohort studies*

Section/Topic	Item #	Recommendation	Reported on page
**Title and abstract**	1	(*a*) Indicate the study's design with a commonly used term in the title or the abstract	Title page
		(*b*) Provide in the abstract an informative and balanced summary of what was done and what was found	2-3
**Introduction**			
Background/rationale	2	Explain the scientific background and rationale for the investigation being reported	5-6
Objectives	3	State specific objectives, including any prespecified hypotheses	6
**Methods**			
Study design	4	Present key elements of study design early in the paper	6-7
Setting	5	Describe the setting, locations, and relevant dates, including periods of recruitment, exposure, follow-up, and data collection	6-7
Participants	6	(*a*) Give the eligibility criteria, and the sources and methods of selection of participants. Describe methods of follow-up	6-7
		(*b*) For matched studies, give matching criteria and number of exposed and unexposed	n/a
Variables	7	Clearly define all outcomes, exposures, predictors, potential confounders, and effect modifiers. Give diagnostic criteria, if applicable	7-8
Data sources/ measurement	8*	For each variable of interest, give sources of data and details of methods of assessment (measurement). Describe comparability of assessment methods if there is more than one group	7-8
Bias	9	Describe any efforts to address potential sources of bias	8-9
Study size	10	Explain how the study size was arrived at	6-7
Quantitative variables	11	Explain how quantitative variables were handled in the analyses. If applicable, describe which groupings were chosen and why	8-9
Statistical methods	12	(*a*) Describe all statistical methods, including those used to control for confounding	8-9
		(*b*) Describe any methods used to examine subgroups and interactions	8-9
		(*c*) Explain how missing data were addressed	9
		(*d*) If applicable, explain how loss to follow-up was addressed	n/a
		(*e*) Describe any sensitivity analyses	n/a
**Results**			

For peer review only

Participants	13*	(a) Report numbers of individuals at each stage of study—eg numbers potentially eligible, examined for eligibility, confirmed eligible, included in the study, completing follow-up, and analysed	
		(b) Give reasons for non-participation at each stage	n/a
		(c) Consider use of a flow diagram	
Descriptive data	14*	(a) Give characteristics of study participants (eg demographic, clinical, social) and information on exposures and potential confounders	9-12
		(b) Indicate number of participants with missing data for each variable of interest	9
		(c) Summarise follow-up time (eg, average and total amount)	n/a
Outcome data	15*	Report numbers of outcome events or summary measures over time	10-11
Main results	16	(*a*) Give unadjusted estimates and, if applicable, confounder-adjusted estimates and their precision (eg, 95% confidence interval). Make clear which confounders were adjusted for and why they were included	12-14
		(*b*) Report category boundaries when continuous variables were categorized	n/a
		(*c*) If relevant, consider translating estimates of relative risk into absolute risk for a meaningful time period	n/a
Other analyses	17	Report other analyses done—eg analyses of subgroups and interactions, and sensitivity analyses	
**Discussion**			
Key results	18	Summarise key results with reference to study objectives	15-16
**Limitations**			
Interpretation	20	Give a cautious overall interpretation of results considering objectives, limitations, multiplicity of analyses, results from similar studies, and other relevant evidence	15-17
Generalisability	21	Discuss the generalisability (external validity) of the study results	15-17
**Other information**			
Funding	22	Give the source of funding and the role of the funders for the present study and, if applicable, for the original study on which the present article is based	18

*Give information separately for cases and controls in case-control studies and, if applicable, for exposed and unexposed groups in cohort and cross-sectional studies.

Note: An Explanation and Elaboration article discusses each checklist item and gives methodological background and published examples of transparent reporting. The STROBE checklist is best used in conjunction with this article (freely available on the Web sites of PLoS Medicine at http://www.plosmedicine.org/, Annals of Internal Medicine at http://www.annals.org/, and Epidemiology at http://www.epidem.com/). Information on the STROBE Initiative is available at www.strobe-statement.org.

For peer review only

BMJ Open

Associations between socio-economic position and changes in children’s screen-viewing between ages 6 and 9: a longitudinal study

Journal:	*BMJ Open*
Manuscript ID	bmjopen-2018-027481.R1
Article Type:	Original research
Date Submitted by the Author:	02-Jul-2019
Complete List of Authors:	Salway, Ruth; University of Bristol, Centre for Exercise, Nutrition and Health Sciences<br>Emm-Collison, Lydia; University of Bristol, Centre for Exercise, Nutrition and Health Sciences<br>Sebire, Simon; University of Bristol, Centre for Exercise, Nutrition and Health Sciences<br>Thompson, Janice; The University of Birmingham, School of Sport & Exercise Sciences<br>Jago, Russ; University of Bristol, Centre for Exercise, Nutrition and Health Sciences
**Primary Subject Heading**:	Public health
Secondary Subject Heading:	Paediatrics
Keywords:	screen-viewing, PAEDIATRICS, TV, computer games, cohort

SCHOLARONE™
Manuscripts

Associations between socio-economic position and changes in children's screen-viewing between ages 6 and 9: a longitudinal study

Ruth Salway[1,*], Lydia Emm-Collison[1], Simon J. Sebire[1], Janice L. Thompson[2] and Russ Jago[1]

[1] Centre for Exercise, Nutrition & Health Sciences, School for Policy Studies, University of Bristol, 8 Priory Road, Bristol, BS8 1TZ, UK.

[2] School of Sport, Exercise and Rehabilitation Sciences, University of Birmingham, Birmingham, B15 2TT, UK

[*] **Corresponding author:** Dr Ruth Salway, Centre for Exercise, Nutrition & Health Sciences, School for Policy Studies, University of Bristol, 8 Priory Road, Bristol, BS8 1TZ, UK.

Email: ruth.salway@bristol.ac.uk; Telephone: +44 (0)117 3310463

Word count: **Manuscript** = 3475 words **Abstract** = 249 words (max 300)

For peer review only

For peer review only - http://bmjopen.bmj.com/site/about/guidelines.xhtml

ABSTRACT

Objectives: To explore socio-economic differences in screen-viewing at age 6 and 9, and how these are related to different media use.

Design: Longitudinal cohort study.

Setting: Children recruited from 57 state-funded primary schools in Southwest England, UK.

Participants: 1299 children at age 5-6, 1223 children at age 8-9, including 685 children at both timepoints.

Outcome measures: Children's total screen-viewing time (parent-reported) and time spent using multiple screen devices simultaneously (multiscreen viewing), for weekdays and weekends.

Methods: Negative binomial regression was used to model associations between socio-economic variables (highest household education and area deprivation) and total screen-viewing at age 6 and the change from age 6 to 9. We additionally adjusted for child characteristics, parental influences and media devices in the home. Multiscreen viewing was analysed separately.

Results: Household education was associated with children's screen-viewing at age 6 and with the change in screen-viewing between ages 6 and 9, with higher screen-viewing in lower socio-economic groups. The differences at age 6 were explained by the presence of games consoles, parental limits on screen-viewing, and average parent screen-viewing. Household education differences in the change in screen-viewing between ages 6 and 9 remained.

Conclusions: Children's screen-viewing patterns differ by parental education with higher levels of viewing among children living in households with lower educational qualifications. These differences are already present at age 6, and continue at age 9. Strategies to manage child sedentary time, and particularly screen-viewing, may need to take account of the socio-economic differences and target strategies to specific groups.

For peer review only - http://bmjopen.bmj.com/site/about/guidelines.xhtml

Keywords: screen viewing, TV, computer games, cohort, children

For peer review only

For peer review only - http://bmjopen.bmj.com/site/about/guidelines.xhtml

ARTICLE SUMMARY

Strengths and limitations of this study

Strengths

- Longitudinal study screen viewing on both weekdays and weekends at two time points.
- Time frame covers an important period of change in child viewing behaviours.
- Data collection period coincided with a period of rapid change in screen-viewing technology which has facilitated the presentation of contemporary data.

Limitations

- We did not explicitly ask parents about tablet or smartphone use and so have not been able to capture this directly.
- A change in the way education was recorded may result in misclassification in a small number of situations where household composition has changed or where people have obtained additional qualifications.

For peer review only

For peer review only - http://bmjopen.bmj.com/site/about/guidelines.xhtml

INTRODUCTION

Sedentary behaviour has been associated with increased risk of obesity and elevated levels of cardio-metabolic risk factors among children and adolescents[1, 2]. Screen-viewing is a very common form of sedentary behaviour among children and young people[3], and higher screen-viewing has been associated with higher body mass index, poorer psychological well-being and poorer academic achievement among children[4-6]. The American Academy of Pediatrics recommends restricting children's screen-viewing, although it does not specify a limit[7]. An international study estimated that 11 year old boys engaged in an average of 5.3 h/day of screen-viewing on weekdays in 2010 (4.4 h/day for girls) with higher estimates at weekends[8]. The paper therefore shows that by the end of primary school children are engaging in high levels of screen viewing. Sedentary behaviour tracks at moderate levels from childhood into adolescence[9], with TV viewing tracking from childhood to adulthood[10]. Moreover, screen-viewing has increased in children by around 1.3h/day on weekdays (2.0 h/day on weekends) between 2002 and 2010 [8]. Thus, screen-viewing is an increasingly common behaviour among children and with levels increasing as children age. We therefore need to understand patterns of behaviour develop during childhood, and particularly at the start of primary school when patterns of behaviour are established. As such, information on levels of screen-viewing and how they change during early school years is required.

Much of the current literature on screen-viewing focuses on TV viewing, but screen-viewing has evolved with technology to include TV viewing, computer games, tablets, mobile phones and multiscreen viewing (in which children use two or more devices at the same time)[11] , and become more diverse, for example encompassing on-demand TV and online gaming[3]. Thus, to try and change behaviour there is a need to understand what devices children are using and the types of viewing in which children engage on different days of the week.

For peer review only - http://bmjopen.bmj.com/site/about/guidelines.xhtml

Socio-economic position has been associated with poorer health outcomes among children and young people. It is important to note, however that different indicators of socio-economic position, such as education, income and deprivation, measure different, often related aspects, although they are strongly correlated[12, 13], and. For example, in England at the end of primary school (age 11) in 2018 the prevalence of child obesity in the most deprived areas is more than double that of those in the least deprived neighbourhoods (27% vs. 12%)[14], and TV viewing has been identified as a mediator for this association[15]. A number of studies have found an inverse relationship between SES and sedentary time[16], sedentary behaviours[17, 18] and screen-viewing specifically[19, 20] with children's daily screen time varying between 1.7 and 2.4 hours/day for high and low SES families respectively[19]. Sedentary behaviour increases as children age[18], but as much of the evidence is cross-sectional it is not clear whether different SES groups change at the same rate. Longitudinal evidence between ages 2 and 9 suggests that while TV viewing increases similarly for different educational subgroups, there may be different trajectories for different household income groups[21]. It is also not clear whether these SES differences are due to differences in other factors or not. Child obesity, ownership of different media devices, parental screen-viewing and parental limits on screen-viewing have all been found to affect children's screen-viewing and differ between SES groups [1, 19, 22-29]. There is thus a need to better understand SES differences, and especially how these change over time.

The aim of this paper was to explore socio-economic differences in screen-viewing at age 6, and the change in screen-viewing between ages 6 and 9, and whether any differences can be explained by other factors such as different media devices and parental influences. Developing knowledge in this area will support the creation of targeted behaviour change programmes to reduce screen-viewing.

METHODS

For peer review only - http://bmjopen.bmj.com/site/about/guidelines.xhtml

B-PROACT1V is a longitudinal study that aimed to examine the physical activity and sedentary behaviours of primary school children aged 5-11 years, and their parents (described in detail elsewhere [30-32]). The study received ethical approval from the School of Policy Studies Ethics Committee at the University of Bristol, UK, and written parental consent was received for all participants [33] (Reference B-Proact1V study, approval 11th November 2011.) In Phase 1, all children in Year 1 of primary school (aged 5-6 years) from 57 schools in and around Bristol were invited to participate, with data collected between January 2012 and July 2013. In Phase 2, when the children were in Year 4 (aged 8-9 years), all schools from Phase 1 were invited to participate, with 47 schools agreeing. All children were eligible regardless of whether they had participated in Phase 1, and data collection took place between March 2015 and July 2016. Data were collected for 1299 children in Year 1 and 1223 children in Year 4, with 685 children included in both phases. Self-identified 'first' parents completed a questionnaire about personal and family characteristics while 'second' parents completed a shorter questionnaire. This paper uses data from the first parent questionnaires: 1085 (84%) from Year 1, 997 (82%) from Year 4 and 509 (74%) at both time points.

Screen-viewing data

In both years, the first parent was asked about the number of hours their child typically spent engaging in specific screen-viewing behaviours on weekdays and at weekends: TV, computer, games consoles. Additionally, both parents were asked about their own screen-viewing. These responses were recorded as either 'None' or in hourly categories from '0-1 hours' up to '4 hours or more', and recoded based on midpoints to give the average number of minutes spent in each type of screen-viewing on weekdays and weekends. These were summed to form the average total number of minutes spent on any type of screen-viewing. Where two parents completed questionnaires, parental screen-viewing was taken as the average (approximately 27% of respondents in each year). At the second phase, parents were also asked about the time they and their child spent multiscreen-viewing (i.e. using two or more devices at once), with responses as above recoded to midpoints [11].

For peer review only

For peer review only - http://bmjopen.bmj.com/site/about/guidelines.xhtml

Socio-economic data

At age 6, the first parent was asked their highest educational qualification, while at age 9, they were asked the highest education qualification of anyone in the household. We combined these to form the highest household educational qualification recorded at either time with categories ‘Up to GCSE (General Certificate of Secondary Education) or equivalent’ (qualification at age 16), ‘A level/National Vocational Qualification or equivalent’ (qualification at age 18), ‘University Degree or equivalent’ and ‘Higher Degree (MSc/PhD) or equivalent’. In addition, Index of Multiple Deprivation (IMD) scores, based on the English Indices of Deprivation (http://data.gov.uk/dataset/index-of-multiple-deprivation), were assigned to each child based on their reported home postcode, with higher IMD scores indicating a greater level of deprivation. Household education captures individual long-term socio-economic position, such as knowledge, and also provides an indirect indication of income, while IMD captures the socio-economic conditions of the area in which they live.

Other measurements

Child gender was reported by the first parent. Child height and weight were recorded to the nearest 0.1 cm and 0.1 kg, respectively, by trained fieldworkers at each time point. Body mass index (BMI) was calculated and converted to an age- and sex-specific standard deviation score based on UK reference curves[34, 35]. The first parent was asked whether they limited the time their child spent engaging in three different types of screen-viewing (TV, computer and video games) with responses from 1 ‘Strongly disagree’ to 4 ‘Strongly agree’. The average of these was used to capture parental limits on screen-viewing. We also asked about the number of media devices in the home: TVs, computers (desktop or laptop), tablet computers and games consoles (including handheld consoles).

Patient and Public Involvement

For peer review only - http://bmjopen.bmj.com/site/about/guidelines.xhtml

The research questions for this study emerged from research that was conducted with 1078 Year 1 children. A sub-set of 53 parents of the children who took part in the Year 1 study participated in interviews to help guide future research. Participants provided verbal assent (with parental consent) to join the study, and summaries of project findings are sent to all participants via study schools.

Statistical Analysis

Descriptive summaries of children's screen-viewing on weekdays and weekends and other participant characteristics were produced by household education group for age 6 and age 9. To aid interpretation, the screen-viewing variables are reported in summaries as continuous variables. However, the underlying variables are discrete and so in the main analyses we used negative binomial regression models (similar to Poisson models but suitable for over-dispersed count data) to model weekday and weekend screen-viewing separately. We considered four main models. Model 1 explored cross-sectional associations between socioeconomic position (household education and IMD) and total minutes of screen-viewing at age 6. Model 2 additionally adjusted for possible mediators and confounders: child gender, child BMI, number of devices in the home (more than one TV, and presence of any computers, tablets or games consoles), parental screen-viewing and parental behaviour on limiting screen-viewing. Models 3 (unadjusted) and 4 (adjusted for covariates) explored the longitudinal change between ages 6 and 9; that is, associations between socioeconomic position and total minutes of screen-viewing at age 9, adjusting for baseline screen-viewing at age 6. Finally, we also explored the association between socioeconomic position and multiscreen viewing as a separate outcome. Robust standard errors were used to account for clustering of children within schools, and all analyses are based on complete cases. Model results are presented as screen time ratios (rate ratios), defined as the exponent of the model coefficients. An increase of one unit in a given predictor variable is associated with a multiplicative increase in screen-viewing, holding the

For peer review only

For peer review only - http://bmjopen.bmj.com/site/about/guidelines.xhtml

other predictor variables in the model constant. Model assumptions were checked via model diagnostics, and all analysis was done in Stata v15.

RESULTS

Children's screen-viewing differed between households with differing levels of education at both ages 6 and 9, with higher screen-viewing in lower qualified households, decreasing as education increased, with 35-50 minutes difference between the highest and lowest household education qualifications (Figure 1). Time spent screen-viewing was higher at age 9 than age 6, and higher at weekends than during the week among all education groups, with time spent watching TV and playing games consoles nearly doubling at weekends (Table S1 and S2). Time spent engaged in multiscreen viewing was also higher for households with lower levels of qualification, with children in households where GCSEs were the highest qualification spending over twice as much time in multiscreen viewing as those where the highest qualification was a postgraduate degree, on both weekdays and weekends. Missing data ranged between 0-3% (Table S3).

There were differences in the number and types of media devices in the home by household education (Figure 2). Households where GCSE and A Levels were the highest qualification had more TVs and games consoles in the house, while Higher Degree households had more computers. There were small increases in most devices between age 6 and age 9. The average number of tablet devices was the same across all levels of household education but increased fourfold to an average of 2.2 per household between the two assessment periods. Parental screen-viewing differed with household education by similar amounts to their children, again with higher screen-viewing among those with lower level qualifications. Parental screen-viewing was also higher at the second time point, especially among the Higher Degree educated households, and differences with education were smaller.

For peer review only - http://bmjopen.bmj.com/site/about/guidelines.xhtml

Table 1 shows the associations between socioeconomic position and total screen-viewing at age 6, in terms of ratios for screen time, which should be interpreted as multiplicative factors for a one-unit increase. For example, a factor of 0.79 for Degree-level household education (Table 1, weekday) means that screen-viewing time decreased by 21% for children in households where a University degree or equivalent was the highest qualification, compared to children in households where the highest qualifications were GCSEs. There were differences in screen-viewing by household education for both weekdays and weekends, with lower screen-viewing for children in households with higher education. The GCSE and A level education groups were similar, but children in households with a Degree or Higher degree engaged in 21-27% less screen-viewing. There was no association with IMD. Model 2 adjusts for possible mediators: number of devices, child BMI, parental screen-viewing and parental limits on screen-viewing all measured at age 6. These factors accounted for household education differences on both weekdays and weekends. Children's screen-viewing was higher in households with games consoles, and lower when parents were more likely to limit screen-viewing (Table S4). There were weak associations with parental screen-viewing and child BMI.

Models 3 and 4 examined at the change in screen-viewing between ages 6 and 9 (Table 2). When adjusting for baseline screen-viewing, there were differences in screen-viewing by household education, with larger increases in screen-viewing for children from A level and Degree households (13% and 6% respectively) and 16% less increase in Higher Degree households, compared to GCSE households in the week. Similar patterns were seen at weekends, with the A level and Degree groups increasing more than the GCSE and Higher Degree groups. There was no association with IMD. When adjusting for media devices in the home, child BMI and parental influences at age 9, household education differences still remain. An increase in screen-viewing was strongly associated with the presence of games consoles and multiple TVs (weekdays only), with weaker associations

For peer review only - http://bmjopen.bmj.com/site/about/guidelines.xhtml

with parental screen-viewing (Table S5). Presence of tablets was associated with a decrease in screen-viewing, as was parental limits on screen-viewing in the week, but not at weekends.

Multiscreen viewing was associated with both IMD but not with household education, with children's multiscreen viewing higher in families living in more deprived areas (Table S6 and Figure S1). When adjusting for other factors, the association with deprivation remained, with multiscreen viewing 26-27% higher for every standard deviation increase in IMD score. Higher amounts of multiscreen viewing were associated with higher levels of parental multiscreen viewing and the presence of tablet devices and multiple TVs in the week, and lower levels of multiscreen viewing were associated with increased parental limits on screen-viewing.

DISCUSSION

The data presented in this paper have shown an association between children's screen-viewing and highest household education level, with higher screen-viewing in lower socio-economic groups. These differences were already present at age 6 and continued to be evident at age 9. Socio-economic differences at age 6 were accounted for by other factors, especially parental factors such as their own screen-viewing and their behaviour in limiting their children's screen-viewing. However, while the time children spent engaged in screen-viewing increased in all households between ages 6 and 9, regardless of education, they increased by different amounts depending on household education. Adjusting for baseline screen-viewing at age 6, screen-viewing increased most over the time period for children in households where the highest qualifications were A Levels or a university degree compared to GCSE, with the smallest increase among Higher Degree households. In A-level educated households, this reflected a "catching-up" with the level of screen-viewing seen in GCSE-educated households. These findings imply socio-economic differences in both amount of screen-viewing, and the way in which screen-viewing patterns develop. These differences were related to

For peer review only - http://bmjopen.bmj.com/site/about/guidelines.xhtml

household education rather than area-level deprivation, suggesting that it is long-term socio-economic aspects such as parental knowledge, ability to engage and communicate with services, and possibly income that are important, rather than the socio-economic conditions of the area, such as availability of local resources. In contrast, multiscreen viewing is associated with area deprivation rather than education and so could represent a proxy for short-term current income or possibly a neighbourhood effect of what is considered to be ‘typical’ screen-viewing behaviour. As such, strategies to manage child sedentary time, and particularly different types of screen-viewing, may need to take account of the socio-economic differences and target strategies to specific groups.

Ownership of media devices such as TVs, phones and games consoles, have previously been found to be associated with higher levels of screen-viewing, and also inversely with socio-economic status [24, 28]. We found that the number of media devices in the home differed by household education, with more TVs and games consoles in households with GCSE and A level qualifications, and more computers in households with a university or higher degree. While the number of devices changed overall between the two assessment points, most notably with a large increase in tablet devices and a slight decrease in the number of computers, socio-economic differences were small and did not account for the observed differences in total screen-viewing between household education. We found that higher screen-viewing at age 6 was associated with games consoles at the weekends only, but not with multiple TVs or computers, and larger increases in screen-viewing between ages 6 and 9 were associated with games consoles and multiple TVs in the week. However, we also saw tablet ownership associated with smaller increases in screen-viewing between ages 6 and 9. This suggests that the relationship between different devices and screen-viewing is complex, and may be changing over time. Thus, we extend previous findings to show that while access to devices may contribute to baseline screen-viewing, it does not entirely explain socio-economic differences in the increase in screen-viewing between ages 6 and 9.

For peer review only

There were weak associations between parent and child screen-viewing, with every 30 minutes of parental screen-viewing associated with a 2% increase in child screen-viewing. Parental limits on screen-time were strongly associated with lower screen-viewing at age 6 and less time spent multi-screen-viewing at age 9, but associations were stronger for weekdays than weekends and limiting screen time was associated with changes in screen-viewing between ages 6 and 9 on weekdays only. Interestingly, analysis of interviews conducted with a sub-sample of the parents in the study showed that many parents are uneasy about managing non-TV screen-time and feel that they struggle to keep up with rapid technological change[36]. Collectively, these findings may suggest that there is a need to help parents to identify effective ways to manage constantly adapting forms of viewing.

Tablet ownership did not vary with household education but increased greatly overall between ages 6 and 9. A limitation of this project is that we did not assess tablet or smartphone use as a specific behaviour in the questionnaire. This may under-estimate total screen-viewing, especially for those who are heavy users of these devices, and may explain the observed negative association between screen-viewing and tablet ownership. We also note that tablet ownership is strongly associated with higher levels of multiscreen-viewing on weekdays. The widespread ownership of tablets is particularly important for understanding the complexity of screen-viewing, as watching TV, playing games and using the internet can all be done via a tablet and, as such, identifying the behaviour engaged in while using a tablet is challenging. Thus, it may be that in the future there is a specific need to assess tablet time, and to differentiate the various ways in which tablets are used. For example, applications that allow parents and children to agree targets to limit the time spent on games and other non-educational activities, could also be used as a feedback and monitoring device.

For peer review only

For peer review only - http://bmjopen.bmj.com/site/about/guidelines.xhtml

306 The major strengths of this study are the information on a variety of different types of screen-viewing at two time points which span an important period of change in child viewing behaviours. The data collection period (2012-16) coincided with a period of rapid change in screen-viewing technology which has facilitated the presentation of contemporary data. There are, however, several limitations that need to be considered. Firstly, we did not explicitly ask parents about tablet or smartphone use. As data collection covers a time when screen use is changing rapidly, (for example, global tablet sales increased from 116 million in 2012[37] to 207 million in 2015[38]) this is an important aspect we have not been able to capture directly. Secondly, the differences in the education variable between Year 1 (first parent's education) and Year 4 (household education) may result in inaccuracies in situations where household composition has changed or where people have obtained additional qualifications between the two time points, although we believe that the number of these cases is likely to be small. Children's screen-viewing was parent-reported, and amounts of multiscreen-viewing, in particular, may not be well-captured, although general patterns might be more robust. This is a longitudinal study, and so some children were lost to follow-up, although the majority were through non-participating schools. In addition, as we have included socioeconomic position variables directly, our models will not biased due to differential follow-up rates by socioeconomic status. Finally, as parental screen-viewing increased between 2012 and 2015 this may indicate a general change in viewing patterns, and the increases reported between the two age periods could reflect secular changes as opposed to age-related differences. As such, it would be important to identify if comparable patterns are evident in other datasets.

Conclusion

Children's screen-viewing patterns differ by parental education with higher levels of viewing among children living in households with lower levels of education. These differences are already present at age 6, and continue at age 9, even when accounting for differences in baseline screen-viewing and

For peer review only - http://bmjopen.bmj.com/site/about/guidelines.xhtml

device ownership. Socio-economic differences narrow with age as children in households with higher qualifications gain greater access to screen-viewing devices. Strategies to manage child sedentary time, and particularly screen-viewing, may need to take account of the socio-economic differences and target strategies to specific groups.

ACKNOWLEDGEMENTS

We would like to thank all of the families and schools that have taken part in the B-PROACT1V project. We would also like to thank all current and previous members of the research team who are not authors on this paper.

COMPETING INTERESTS

We have no competing interest to report.

FUNDING

This work was supported by the British Heart Foundation (ref PG/11/51/28986 and SP 14/4/31123). The funder had no involvement in data analysis, data interpretation or writing of the paper.

AUTHOR CONTRIBUTIONS

RJ, SJS and JLT were involved in the design of this study and in seeking funding for it. The paper was conceived by RJ and RS, and RS performed all analyses. RS and RJ wrote the first draft of the paper and RJ coordinated contributions from other authors. All authors (RJ, SJS, JLT, LEC) made critical comments on drafts of the paper.

DATA SHARING STATEMENT

The datasets generated during the current study are not publicly available due as the project is ongoing and data are not ready for archiving. We will consider reasonable requests for access to the data once the project is complete in 2019.

For peer review only

REFERENCES

1. Ekelund U, Brage S, Froberg K, Harro M, Anderssen S, Sardinha LB, et al. TV Viewing and Physical Activity Are Independently Associated with Metabolic Risk in Children: The European Youth Heart Study. PLoS Med. 2006;3:e488.

2. Ekelund U, Luan J, Sherar LB, Esliger DW, Griew P, Cooper A, et al. Moderate to vigorous physical activity and sedentary time and cardiometabolic risk factors in children and adolescents. JAMA. 2012;307:704-12. doi: 10.1001/jama.2012.156.

3. Saunders TJ, Vallance JK. Screen Time and Health Indicators Among Children and Youth: Current Evidence, Limitations and Future Directions. Appl Health Econ Health Policy. 2017;15:323-31. doi: 10.1007/s40258-016-0289-3.

4. Corder K, Atkin AJ, Bamber DJ, Brage S, Dunn VJ, Ekelund U, et al. Revising on the run or studying on the sofa: prospective associations between physical activity, sedentary behaviour, and exam results in British adolescents. Int J Behav Nutr Phys Act. 2015;12:106. doi: 10.1186/s12966-015-0269-2.

5. Page AS, Cooper AR, Griew P, Jago R. Children's Screen Viewing is Related to Psychological Difficulties Irrespective of Physical Activity. Pediatrics. 2010;126:e1011-7.

6. Jago R, Baranowski T, Baranowski JC, Thompson D, Greaves KA. BMI from 3-6 y of age is predicted by TV viewing and physical activity, not diet. Int J Obes (Lond). 2005;29:557-64. doi: 10.1038/sj.ijo.0802969.

7. American Academy of Pediatrics. Media Use in School-Aged Children and Adolescents. Pediatrics. 2016;138:e20162591.

8. Bucksch J, Sigmundova D, Hamrik Z, Troped PJ, Melkevik O, Ahluwalia N, et al. International Trends in Adolescent Screen-Time Behaviors From 2002 to 2010. J Adolesc Health. 2016;58:417-25. doi: 10.1016/j.jadohealth.2015.11.014.

For peer review only

For peer review only - http://bmjopen.bmj.com/site/about/guidelines.xhtml

9. Biddle SJ, Pearson N, Ross GM, Braithwaite R. Tracking of sedentary behaviours of young people: a systematic review. Prev Med. 2010;51:345-51. doi: 10.1016/j.ypmed.2010.07.018.

10. Smith L, Gardner B, M. H. Childhood correlates of adult TV viewing time: a 32-year follow-up of the 1970 British Cohort Study. Journal of epidemiology and community health. 2015;69:309-13. doi: 10.1136/jech-2014-204365.

11. Jago R, Sebire SJ, Gorely T, Cillero IH, Biddle SJ. "I'm on it 24/7 at the moment": a qualitative examination of multi-screen viewing behaviours among UK 10-11 year olds. Int J Behav Nutr Phys Act. 2011;8:85. doi: 10.1186/1479-5868-8-85.

12. Galobardes B, Shaw M, Lawlor DA, Lynch JW, Davey Smith G. Indicators of socioeconomic position (part 1). Journal of epidemiology and community health. 2006;60:7-12. doi: 10.1136/jech.2004.023531.

13. Galobardes B, Shaw M, Lawlor DA, Lynch JW, Davey Smith G. Indicators of socioeconomic position (part 2). Journal of epidemiology and community health. 2006;60:95-101. doi: 10.1136/jech.2004.028092.

14. NHS Digital. National Child Measurement Programme, England 2017-2018 school year. UK: 2018.

15. Morgenstern M, Sargent JD, Hanewinkel R. Relation between socioeconomic status and body mass index: evidence of an indirect path via television use. Archives of pediatrics & adolescent medicine. 2009;163:731-8. doi: 10.1001/archpediatrics.2009.78.

16. Sherar LB, Griffin TP, Ekelund U, Cooper AR, Esliger DW, van Sluijs EM, et al. Association between maternal education and objectively measured physical activity and sedentary time in adolescents. Journal of epidemiology and community health. 2016;70:541-8. doi: 10.1136/jech-2015-205763.

For peer review only

For peer review only - http://bmjopen.bmj.com/site/about/guidelines.xhtml

17. Coombs N, Shelton N, Rowlands A, Stamatakis E. Children's and adolescents' sedentary behaviour in relation to socioeconomic position. Journal of epidemiology and community health. 2013;67:868-74. doi: 10.1136/jech-2013-202609.

18. Moraeus L, Lissner L, Olsson L, Sjoberg A. Age and time effects on children's lifestyle and overweight in Sweden. BMC Public Health. 2015;15:355. doi: 10.1186/s12889-015-1635-3.

19. Tandon P, Chuan Zhou C, Sallis J, Cain K, Frank L, Saelens B. Home environment relationships with children's physical activity, sedentary time, and screen time by socioeconomic status. Int J Behav Nutr Phys Act. 2012;9:88.

20. Drenowatz C, Eisenmann JC, Pfeiffer KA, Welk G, Heelan K, Gentile D, et al. Influence of socio-economic status on habitual physical activity and sedentary behavior in 8- to 11-year old children. BMC Public Health. 2010;10:214.

21. Yang-Huang J, van Grieken A, Moll HA, Jaddoe VWV, Wijtzes AI, Raat H. Socioeconomic differences in children's television viewing trajectory: A population-based prospective cohort study. PloS one. 2017;12:e0188363. doi: 10.1371/journal.pone.0188363.

22. Jago R, Page A, Froberg K, Sardinha L, Klasson-Heggebo L, Andersen L. Screen-viewing and the home TV environment: The European Youth Heart Study. Prev Med. 2008;47:525 - 9.

23. Jago R, Davison K, Thompson JL, Page AS, Brockman R, Fox KR. Parental sedentary restriction, maternal parenting style and TV viewing among 10-11 year olds Pediatrics. 2011;128:e572-e8.

24. Jago R, Stamatakis E, Gama A, Carvalhal IM, Nogueira H, Rosado V, et al. Parent and child screen-viewing time and home media environment. Am J Prev Med. 2012;43:150-8. doi: 10.1016/j.amepre.2012.04.012.

25. Jago R, Sebire SJ, Edwards MJ, Thompson JL. Parental TV viewing, parental self-efficacy, media equipment and TV viewing among preschool children. Eur J Pediatr. 2013;172:1543-5. doi: 10.1007/s00431-013-2077-5.

For peer review only

For peer review only - http://bmjopen.bmj.com/site/about/guidelines.xhtml

26. Jago R, Thompson JL, Sebire SJ, Wood L, Pool L, Zahra J, et al. Cross-sectional associations between the screen-time of parents and young children: differences by parent and child gender and day of the week. International Journal of Behavioral Nutrition and Physical Activity. 2014;11. doi: 10.1186/1479-5868-11-54.

27. Lauricella AR, Wartella E, Rideout VJ. Young children's screen time: The complex role of parent and child factors. Journal of Applied Developmental Psychology. 2015;36:11-7. doi: 10.1016/j.appdev.2014.12.001.

28. Dumuid D, Olds TS, Lewis LK, Maher C. Does home equipment contribute to socioeconomic gradients in Australian children's physical activity, sedentary time and screen time? BMC Public Health. 2016;16:736. doi: 10.1186/s12889-016-3419-9.

29. Gebremariam MK, Altenburg TM, Lakerveld J, Andersen LF, Stronks K, Chinapaw MJ, et al. Associations between socioeconomic position and correlates of sedentary behaviour among youth: a systematic review. Obes Rev. 2015;16:988-1000. doi: 10.1111/obr.12314.

30. Jago R, Sebire SJ, Wood L, Pool L, Zahra J, Thompson JL, et al. Associations between objectively assessed child and parental physical activity: a cross-sectional study of families with 5-6 year old children. BMC public health. 2014;14:655. doi: 10.1186/1471-2458-14-655.

31. Jago R, Thompson JL, Sebire SJ, Wood L, Pool L, Zahra J, et al. Cross-sectional associations between the screen-time of parents and young children: differences by parent and child gender and day of the week. Int J Behav Nutr Phys Act. 2014;11:54. doi: 10.1186/1479-5868-11-54.

32. Jago R, Solomon-Moore E, Macdonald-Wallis C, Sebire SJ, Thompson JL, Lawlor DA. Change in children's physical activity and sedentary time between Year 1 and Year 4 of primary school in the B-PROACT1V cohort. Int J Behav Nutr Phys Act. 2017;14:33. doi: 10.1186/s12966-017-0492-0.

33. Jago R, Bailey R. Ethics and paediatric exercise science: Issues and making a submission to a local ethics and research committee. Journal of Sport Sciences. 2001;19:527-35.

For peer review only

For peer review only - http://bmjopen.bmj.com/site/about/guidelines.xhtml

34. Cole TJ, Freeman JV, Preece MA. Body mass index reference curves for the UK, 1990. Arch Dis Child. 1995;73:25-9.

35. Cole TJ, Bellizzi MC, Flegal KM, H. DW. Establishing a standard definition for child overweight and obesity worldwide: international survey. BMJ Open. 2000;320:1240-3.

36. Jago R, Solomon-Moore E, Toumpakari Z, Lawlor DA, Thompson JL, Sebire SJ. Parents' perspectives of change in child physical activity & screen-viewing between Y1 (5-6) & Y4 (8-9) of primary school: implications for behaviour change. BMC public health. 2018;18:520. doi: 10.1186/s12889-018-5445-2.

37. Gartner. Gartner Says Worldwide Tablet Sales Grew 68 Percent in 2013, With Android Capturing 62 Percent of the Market 2014 [cited 2018 18 Sep]. Available from: https://www.gartner.com/newsroom/id/2674215.

38. IDC. Worldwide Shipments of Slate Tablets Continue to Decline While Detachable Tablets Climb to New High, According to IDC 2016 [cited 2018 18 Sep]. Available from: https://web.archive.org/web/20160204014026/http://www.idc.com/getdoc.jsp?containerId=prUS40990116.

For peer review only

For peer review only - http://bmjopen.bmj.com/site/about/guidelines.xhtml

Table 1 – Associations between socioeconomic position and screen-viewing at age 6

	Model 1: unadjusted			Model 2: adjusted[1]		
	Ratio	95% CI	p-value	Ratio	95% CI	p-value
**WEEKDAY**						
Household education						
Up to GCSE	1	(Reference)		1	(Reference)	
A level	0.94	(0.85, 1.03)		0.98	(0.89, 1.08)	
Degree	0.79	(0.70, 0.90)		0.88	(0.78, 1.00)	
Higher degree	0.73	(0.60, 0.88)	<0.0005	0.88	(0.74, 1.05)	0.054
Deprivation (IMD)[2]	1.04	(1.00, 1.08)	0.067	1.02	(0.98, 1.07)	0.296
**WEEKEND**						
Household education						
Up to GCSE	1	(Reference)		1	(Reference)	
A level	1.00	(0.91, 1.10)		1.03	(0.95, 1.12)	
Degree	0.88	(0.79, 0.98)		1.02	(0.93, 1.13)	
Higher degree	0.77	(0.67, 0.90)	0.001	0.99	(0.87, 1.11)	0.725
Deprivation (IMD)[2]	1.02	(0.98, 1.06)	0.387	1.01	(0.96, 1.06)	0.720

[1] Adjusted for child gender, child BMI, presence of TVs, computers, tablets and games consoles in the household, parental screen-viewing and parental limiting of screen-viewing

[2] increase per 1 standard deviation (14.0) in IMD: higher values indicate more deprived areas

For peer review only

Table 2 – Associations between socioeconomic position and screen-viewing at age 9, adjusting for baseline screen-viewing at age 6

	Model 3: unadjusted			Model 4: adjusted[1]		
	Ratio	95% CI	p-value	Ratio	95% CI	p-value
**WEEKDAY**						
Household education						
Up to GCSE	1	(Reference)		1	(Reference)	
A level	1.13	(1.01, 1.26)		1.16	(1.03, 1.32)	
Degree	1.06	(0.92, 1.22)		1.10	(0.96, 1.26)	
Higher degree	0.84	(0.70, 0.99)	0.001	0.90	(0.77, 1.05)	0.008
Deprivation (IMD)[2]	1.06	(1.00, 1.13)	0.053	1.05	(1.00, 1.10)	0.038
**WEEKEND**						
Household education						
Up to GCSE	1	(Reference)		1	(Reference)	
A level	1.14	(1.00, 1.30)		1.14	(1.01, 1.28)	
Degree	1.09	(0.96, 1.23)		1.11	(0.99, 1.24)	
Higher degree	0.96	(0.83, 1.10)	0.004	0.98	(0.87, 1.11)	0.006
Deprivation (IMD)[2]	1.02	(0.98, 1.07)	0.346	1.00	(0.95, 1.05)	0.938

[1] Adjusted for child gender, presence of TVs, computers, tablets and games consoles in the household, parental screen-viewing and parental limiting of screen-viewing
[2] increase per 1 standard deviation (14.0) in IMD: higher values indicate more deprived areas

For peer review only

For peer review only - http://bmjopen.bmj.com/site/about/guidelines.xhtml

FIGURE LEGENDS AND KEYS

Figure 1: Boxplots of children's total minutes screen-viewing by household education at ages 6 and 9, for weekdays (left) and weekends (right).

Figure 2: Average number of devices in the home by household education at ages 6 (left) and 9 (right).

For peer review only

For peer review only - http://bmjopen.bmj.com/site/about/guidelines.xhtml

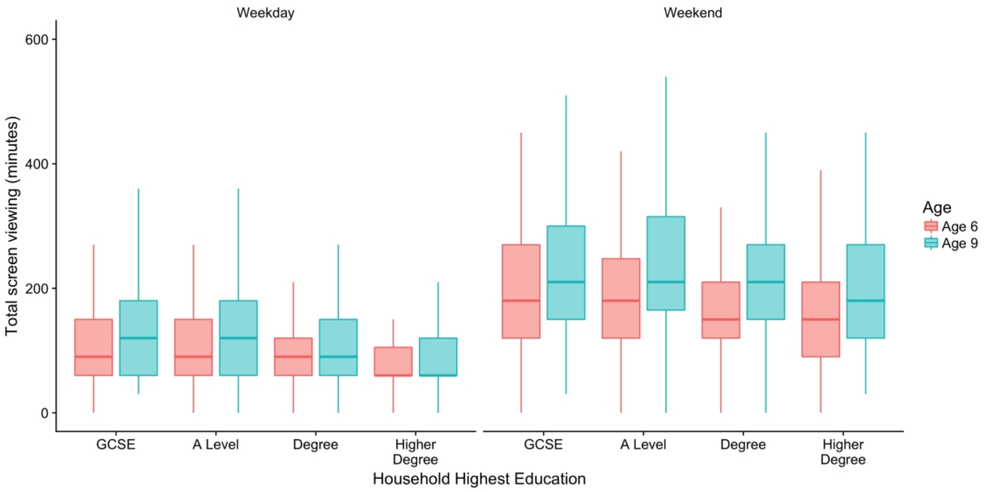

Boxplots of children's total minutes screen-viewing by household education at ages 6 and 9, for weekdays (left) and weekends (right).

1270x635mm (72 x 72 DPI)

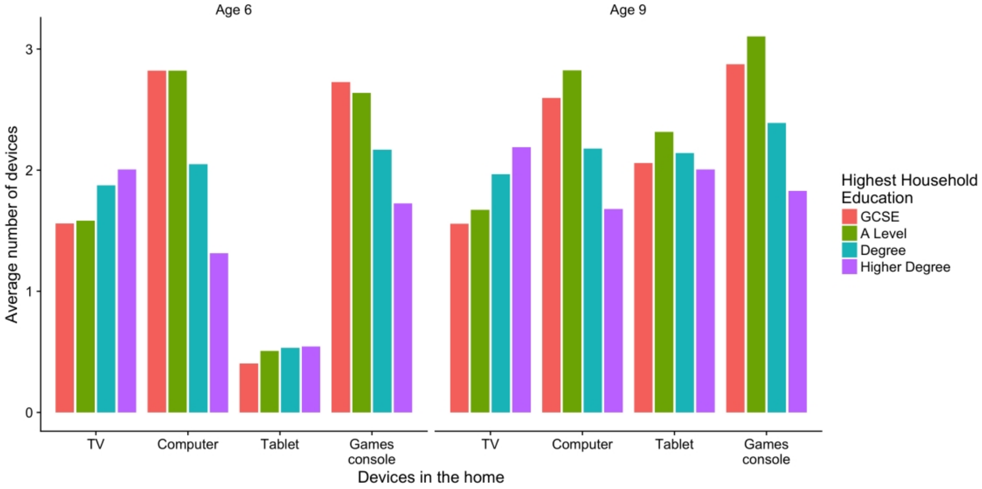

Average number of devices in the home by household education at ages 6 (left) and 9 (right).

1270x635mm (72 x 72 DPI)

SUPPLEMENTARY MATERIAL

ASSOCIATIONS BETWEEN SOCIO-ECONOMIC POSITION AND CHANGES IN CHILDREN'S SCREEN-VIEWING BETWEEN AGES 6 AND 9: A LONGITUDINAL STUDY

Ruth Salway, Lydia Emm-Collison, Simon J. Sebire, Janice L. Thompson and Russell Jago

For peer review only

List of Supplementary Tables

Table S1 – Characteristics at age 6

Table S2 - Characteristics at age 9

Table S3 – Missing values

Table S4 – full details of adjusted model for screen-viewing at age 6

Table S5 – full details of adjusted model for screen-viewing at age 9

Table S6– Associations between socioeconomic position and multi-screen-viewing at age 9

List of Supplementary Figures

Figure S1: Number of minutes spent multi-screen viewing by household education at age 9, for weekdays (left) and weekends (right).

Table S1 – Characteristics at age 6

	Up to GCSE or equiv	A Levels or equiv	Degree or equiv	Higher Degree or equiv	All
	Mean (sd) or %	Mean (sd) or %	Mean (sd) or %	Mean (sd) or %	Mean (sd) or %
WEEKDAY					
Total SV (mins)	120 (74)	111 (61)	93 (60)	85 (70)	102 (66)
TV (mins)	73 (53)	69 (45)	60 (41)	53 (44)	64 (46)
Computer (mins)	25 (28)	23 (27)	20 (22)	23 (33)	22 (26)
Games consoles (mins)	23 (30)	18 (20)	14 (25)	10 (20)	16 (24)
WEEKEND					
Total SV (mins)	196 (118)	193 (107)	169 (94)	150 (102)	178 (105)
TV (mins)	117 (78)	120 (72)	110 (69)	89 (68)	111 (72)
Computer (mins)	37 (44)	34 (38)	32 (33)	37 (39)	34 (37)
Games consoles (mins)	43 (55)	40 (45)	28 (39)	24 (39)	33 (45)
IMD	19.0 (15.5)	16.6 (13.7)	12.2 (10.4)	10.4 (7.4)	14.6 (12.5)
Female	45%	47%	50%	47%	48%
BMI z-score	0.49 (1.04)	0.18 (0.88)	0.16 (0.88)	0.24 (0.94)	0.23 (0.93)
No. devices in home:					
TVs	2.7 (1.3)	2.6 (1.3)	2.2 (1.1)	1.7 (1.1)	2.3 (1.2)
Computers	1.6 (1.1)	1.6 (0.9)	1.9 (1.0)	2.0 (1.1)	1.8 (1.0)
Tablets	0.4 (0.7)	0.5 (0.8)	0.5 (0.7)	0.5 (0.7)	0.5 (0.7)
Games consoles	2.8 (1.8)	2.8 (1.7)	2.0 (1.6)	1.3 (1.3)	2.3 (1.7)
Parent SV (mins)					
Weekday	231 (141)	230 (134)	220 (112)	202 (115)	222 (125)
Weekend	253 (146)	258 (124)	228 (103)	209 (113)	235 (120)
Parental limits on SV[1]	3.3 (0.6)	3.3 (0.6)	3.4 (0.6)	3.5 (0.6)	3.3 (0.6)

[1] higher values indicate more limits on screen viewing (between 1 and 4)

For peer review only

Table S2 - Characteristics at age 9

	Up to GCSE or equiv	A Levels or equiv	Degree or equiv	Higher Degree or equiv	All
	Mean (sd) or %	Mean (sd) or %	Mean (sd) or %	Mean (sd) or %	Mean (sd) or %
WEEKDAY					
Total SV (mins)	139 (94)	133 (78)	113 (75)	88 (59)	118 (78)
TV (mins)	75 (55)	72 (49)	63 (46)	48 (38)	65 (48)
Computer (mins)	37 (46)	34 (37)	31 (37)	29 (31)	32 (37)
Games consoles (mins)	28 (44)	26 (34)	19 (30)	12 (24)	21 (33)
Multi-screen viewing (mins)	29 (46)	24 (40)	16 (33)	11 (25)	19 (37)
WEEKEND					
Total SV (mins)	244 (134)	245 (120)	227 (118)	200 (118)	230 (122)
TV (mins)	127 (72)	126 (66)	125 (63)	104 (62)	122 (65)
Computer (mins)	59 (66)	58 (64)	53 (60)	57 (54)	56 (61)
Games consoles (mins)	58 (68)	62 (63)	49 (62)	39 (54)	52 (62)
Multi-screen viewing (mins)	53 (73)	48 (66)	33 (61)	23 (45)	39 (63)
IMD	20.1 (15.8)	17.6 (15.0)	12.8 (11.9)	12.0 (10.3)	15.1 (13.6)
Female	56%	52%	56%	53%	55%
BMI z-score	0.44 (1.15)	0.28 (1.04)	0.22 (1.06)	0.28 (1.02)	0.31 (1.06)
No. devices in the home					
TVs	2.9 (1.3)	3.1 (1.4)	2.4 (1.3)	1.8 (1.1)	2.6 (1.4)
Computers	1.6 (1.2)	1.7 (1.1)	2.0 (1.2)	2.2 (1.1)	1.9 (1.2)
Tablets	2.1 (1.2)	2.3 (1.3)	2.1 (1.2)	2.0 (1.2)	2.2 (1.3)
Games consoles	2.6 (2.1)	2.8 (1.8)	2.2 (1.6)	1.7 (1.6)	2.3 (1.8)
Parent SV (mins)					
Weekday	248 (134)	245 (128)	248 (134)	253 (144)	248 (134)
Weekend	280 (154)	287 (135)	260 (130)	252 (144)	270 (138)
Parental limits on SV[1]	3.1 (0.7)	3.2 (0.6)	3.4 (0.6)	3.4 (0.5)	3.3 (0.6)

[1] higher values indicate more limits on screen viewing (between 1 and 4)

For peer review only

Table S3 – Missing values

	Age 6		Age 9		Data at both time points Age 6		Data at both time points Age 9	
	N	% missing	N	% missing	N	% missing	N	% missing
Total	1085		997		509		509	
Household education	1071	1%	992	0.2%	509	0%	509	0%
IMD	1069	1%	983	1%	501	2%	499	2%
**WEEKDAY**								
Total SV (mins)	1069	1%	979	2%	504	1%	502	1%
Multi-SV (mins)			981	2%			503	1%
Parent SV (mins)	1075	1%	981	2%	505	1%	503	1%
Parent multi-SV (mins)			983	1%			503	1%
**WEEKEND**								
Total SV (mins)	1065	2%	978	2%	500	2%	501	2%
Multi-SV (mins)			981	2%			503	1%
Parent SV (mins)	1068	2%	979	2%	505	1%	501	2%
Parent multi-SV (mins)			983	1%			503	1%
Gender	1085	0%	997	0%	509	0%	509	0%
BMI z-score	1072	1%	993	0.4%	502	1%	508	0.2%
No. TVs	1073	1%	982	2%	507	0.4%	503	1%
No. Computers	1058	2%	982	2%	504	1%	503	1%
No. Tablets	1062	2%	981	2%	504	1%	503	1%
No. games consoles	1056	3%	981	2%	500	2%	503	1%
Parental limits on SV	1064	2%	971	3%	495	3%	497	2%

For peer review only

Table S4 – full details of adjusted model for screen-viewing at age 6

	WEEKDAY			WEEKEND		
	Ratio	95% CI	p-value	Ratio	95% CI	p-value
Household education						
Up to GCSE	1	(Reference)		1	(Reference)	
A level	0.98	(0.89, 1.08)		1.03	(0.95, 1.12)	
Degree	0.88	(0.78, 1.00)		1.02	(0.93, 1.13)	
Higher degree	0.88	(0.74, 1.05)	0.054	0.99	(0.87, 1.11)	0.725
Deprivation (IMD)[1]	1.02	(0.98, 1.07)	0.296	1.01	(0.96, 1.06)	0.720
>1 TV	0.99	(0.90, 1.10)	0.924	1.04	(0.95, 1.13)	0.394
Any computers	0.94	(0.74, 1.21)	0.645	0.86	(0.73, 1.02)	0.080
Any tablets	1.02	(0.94, 1.10)	0.652	1.01	(0.94, 1.08)	0.811
Any games consoles	1.15	(1.00, 1.32)	0.043	1.42	(1.26, 1.60)	<0.0005
Child gender	0.87	(0.81, 0.94)	<0.0005	0.82	(0.78, 0.87)	<0.0005
z-BMI score	1.07	(1.02, 1.12)	0.003	1.04	(1.01, 1.07)	0.021
parent SV (mins)[2]	1.04	(1.03, 1.05)	<0.0005	1.05	(1.04, 1.07)	<0.0005
Parental limits on SV	0.77	(0.72, 0.83)	<0.0005	0.85	(0.81, 0.90)	<0.0005

[1] increase per 1 standard deviation (14.0) in IMD: higher values indicate more deprived areas

[2] increase per 30 minutes screen-viewing

For peer review only

Table S5 – full details of adjusted model for screen-viewing at age 9

	WEEKDAY			WEEKEND		
	Ratio	95% CI	p-value	Ratio	95% CI	p-value
Household education						
Up to GCSE	1	(Reference)		1	(Reference)	
A level	1.16	(1.03, 1.32)		1.14	(1.01, 1.28)	
Degree	1.10	(0.96, 1.26)		1.11	(0.99, 1.24)	
Higher degree	0.90	(0.77, 1.05)	0.008	0.98	(0.87, 1.11)	0.006
Deprivation (IMD)[1]	1.05	(1.00, 1.10)	0.038	1.00	(0.95, 1.05)	0.938
Baseline SV [2]	1.08	(1.05, 1.10)	<0.0005	1.05	(1.03, 1.06)	<0.0005
>1 TV	1.16	(1.06, 1.27)	0.001	1.06	(0.96, 1.18)	0.249
Any computers	1.03	(0.86, 1.24)	0.721	1.07	(0.92, 1.26)	0.386
Any tablets	0.75	(0.64, 0.88)	<0.0005	0.80	(0.68, 0.95)	0.009
Any games consoles	1.26	(1.11, 1.44)	<0.0005	1.27	(1.08, 1.49)	0.003
Child gender	0.98	(0.91, 1.06)	0.517	0.89	(0.83, 0.96)	0.002
z-BMI score	1.00	(0.95, 1.05)	0.966	1.00	(0.96, 1.03)	0.904
parent SV (mins)[2]	1.03	(1.02, 1.04)	<0.0005	1.04	(1.03, 1.05)	<0.0005
Parental limits on SV	0.82	(0.76, 0.89)	<0.0005	0.93	(0.86, 1.01)	0.084

[1] increase per 1 standard deviation (14.0) in IMD: higher values indicate more deprived areas
[2] increase per 30 minutes screen-viewing at age 6
[3] increase per 30 minutes screen-viewing

For peer review only

Table S6– Associations between socioeconomic position and multi-screen-viewing at age 9

	WEEKDAY			WEEKEND		
	Ratio	95% CI	p-value	Ratio	95% CI	p-value
**Unadjusted Model**						
Household education						
Up to GCSE	1	(Reference)		1	(Reference)	
A level	0.82	(0.49, 1.37)		0.87	(0.53, 1.42)	
Degree	0.66	(0.38, 1.15)		0.63	(0.37, 1.06)	
Higher degree	0.52	(0.26, 1.05)	0.226	0.55	(0.31, 0.97)	0.083
Deprivation (IMD)[1]	1.42	(1.21, 1.67)	<0.0005	1.33	(1.16, 1.52)	<0.0005
**Adjusted Model[2]**						
Household education						
Up to GCSE	1	(Reference)		1	(Reference)	
A level	0.92	(0.51, 1.65)		0.90	(0.50, 1.59)	
Degree	0.80	(0.45, 1.42)		0.72	(0.38, 1.34)	
Higher degree	0.76	(0.34, 1.71)	0.808	0.64	(0.33, 1.25)	0.432
Deprivation (IMD)[1]	1.26	(1.12, 1.41)	<0.0005	1.27	(1.13, 1.43)	<0.0005
>1 TV	1.76	(1.12, 2.75)	0.014	1.38	(0.88, 2.16)	0.158
Any computers	0.70	(0.41, 1.20)	0.190	0.97	(0.54, 1.75)	0.920
Any tablets	1.90	(1.12, 3.22)	0.017	1.34	(0.46, 3.88)	0.590
Any games consoles	1.02	(0.59, 1.78)	0.943	1.65	(0.95, 2.85)	0.076
Child gender	1.01	(0.73, 1.40)	0.945	1.06	(0.76, 1.49)	0.730
z-BMI score	1.08	(0.92, 1.25)	0.348	0.93	(0.82, 1.05)	0.246
parent multi-SV (mins)[2]	1.26	(1.17, 1.35)	<0.0005	1.21	(1.13, 1.29)	<0.0005
Parental limits on SV	0.62	(0.50, 0.78)	<0.0005	0.72	(0.53, 0.97)	0.033

[1] increase per 1 standard deviation (14.0) in IMD: higher values indicate more deprived areas
[2] Adjusted for child gender, child BMI, presence of TVs, computers, tablets and games consoles in the household, parental screen-viewing and parental limiting of screen-viewing
[3] increase per 30 minutes multi-screen-viewing

For peer review only

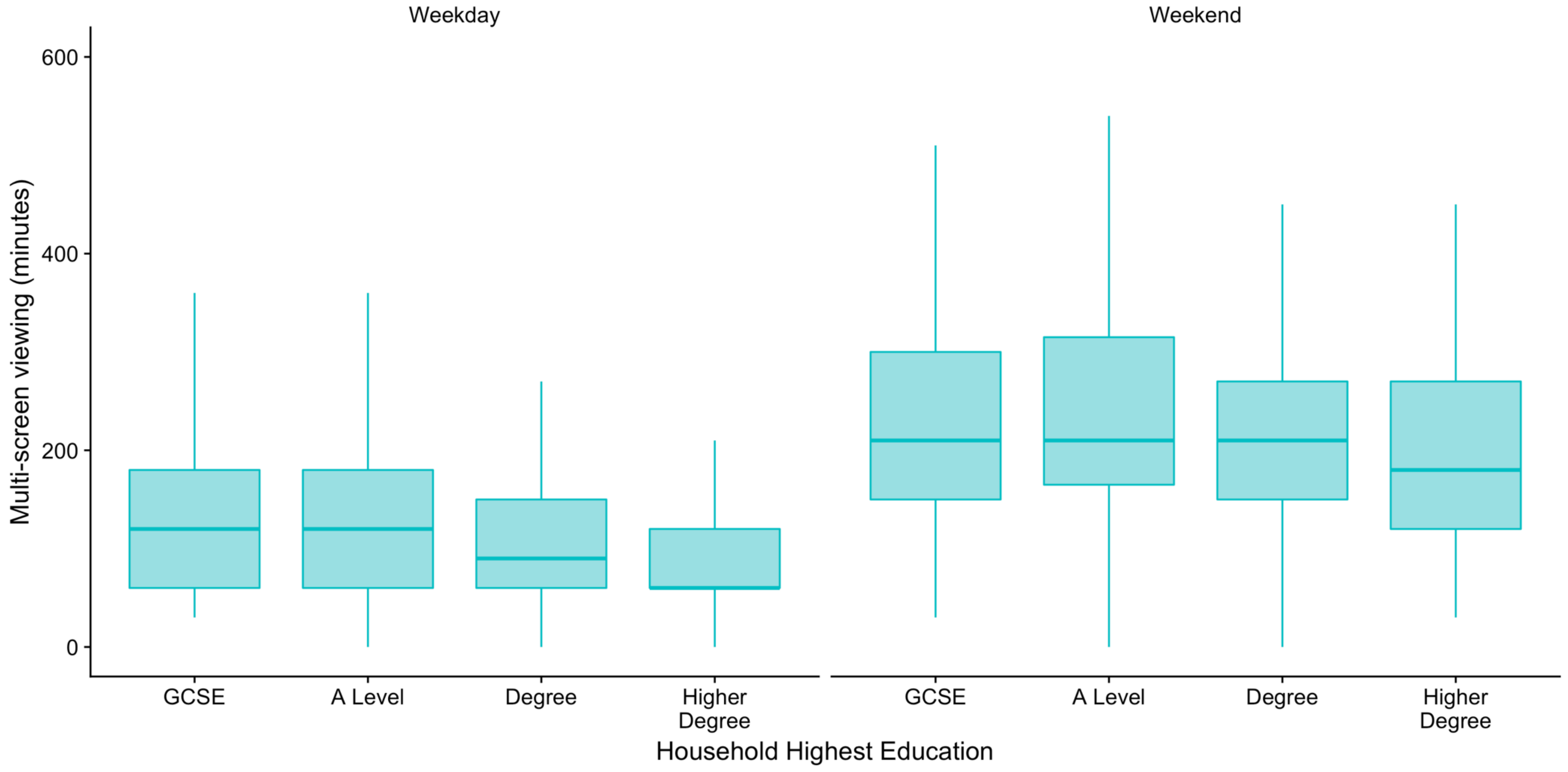

Figure S1: Number of minutes spent multi-screen viewing by household education at age 9, for weekdays (left) and weekends (right).

STROBE 2007 (v4) Statement—Checklist of items that should be included in reports of *cohort studies*

Section/Topic	Item #	Recommendation	Reported on page #
**Title and abstract**	1	(*a*) Indicate the study's design with a commonly used term in the title or the abstract	Title page
		(*b*) Provide in the abstract an informative and balanced summary of what was done and what was found	2-3
**Introduction**			
Background/rationale	2	Explain the scientific background and rationale for the investigation being reported	5-6
Objectives	3	State specific objectives, including any prespecified hypotheses	6
**Methods**			
Study design	4	Present key elements of study design early in the paper	6-7
Setting	5	Describe the setting, locations, and relevant dates, including periods of recruitment, exposure, follow-up, and data collection	6-7
Participants	6	(*a*) Give the eligibility criteria, and the sources and methods of selection of participants. Describe methods of follow-up	6-7
		(*b*) For matched studies, give matching criteria and number of exposed and unexposed	n/a
Variables	7	Clearly define all outcomes, exposures, predictors, potential confounders, and effect modifiers. Give diagnostic criteria, if applicable	7-8
Data sources/ measurement	8*	For each variable of interest, give sources of data and details of methods of assessment (measurement). Describe comparability of assessment methods if there is more than one group	7-8
Bias	9	Describe any efforts to address potential sources of bias	8-9
Study size	10	Explain how the study size was arrived at	6-7
Quantitative variables	11	Explain how quantitative variables were handled in the analyses. If applicable, describe which groupings were chosen and why	8-9
Statistical methods	12	(*a*) Describe all statistical methods, including those used to control for confounding	8-9
		(*b*) Describe any methods used to examine subgroups and interactions	8-9
		(*c*) Explain how missing data were addressed	9
		(*d*) If applicable, explain how loss to follow-up was addressed	n/a
		(*e*) Describe any sensitivity analyses	n/a
**Results**			

For peer review only

Participants	13*	(a) Report numbers of individuals at each stage of study—eg numbers potentially eligible, examined for eligibility, confirmed eligible, included in the study, completing follow-up, and analysed	7
		(b) Give reasons for non-participation at each stage	n/a
		(c) Consider use of a flow diagram	
Descriptive data	14*	(a) Give characteristics of study participants (eg demographic, clinical, social) and information on exposures and potential confounders	9-11
		(b) Indicate number of participants with missing data for each variable of interest	9
		(c) Summarise follow-up time (eg, average and total amount)	n/a
Outcome data	15*	Report numbers of outcome events or summary measures over time	9-10
Main results	16	(*a*) Give unadjusted estimates and, if applicable, confounder-adjusted estimates and their precision (eg, 95% confidence interval). Make clear which confounders were adjusted for and why they were included	11-12
		(*b*) Report category boundaries when continuous variables were categorized	n/a
		(*c*) If relevant, consider translating estimates of relative risk into absolute risk for a meaningful time period	n/a
Other analyses	17	Report other analyses done—eg analyses of subgroups and interactions, and sensitivity analyses	
**Discussion**			
Key results	18	Summarise key results with reference to study objectives	12-13
**Limitations**			
Interpretation	20	Give a cautious overall interpretation of results considering objectives, limitations, multiplicity of analyses, results from similar studies, and other relevant evidence	13-14
Generalisability	21	Discuss the generalisability (external validity) of the study results	14-15
**Other information**			
Funding	22	Give the source of funding and the role of the funders for the present study and, if applicable, for the original study on which the present article is based	16

*Give information separately for cases and controls in case-control studies and, if applicable, for exposed and unexposed groups in cohort and cross-sectional studies.

Note: An Explanation and Elaboration article discusses each checklist item and gives methodological background and published examples of transparent reporting. The STROBE checklist is best used in conjunction with this article (freely available on the Web sites of PLoS Medicine at http://www.plosmedicine.org/, Annals of Internal Medicine at http://www.annals.org/, and Epidemiology at http://www.epidem.com/). Information on the STROBE Initiative is available at www.strobe-statement.org.

For peer review only

BMJ Open

Associations between socio-economic position and changes in children's screen-viewing between ages 6 and 9: a longitudinal study

Journal:	*BMJ Open*
Manuscript ID	bmjopen-2018-027481.R2
Article Type:	Original research
Date Submitted by the Author:	14-Aug-2019
Complete List of Authors:	Salway, Ruth; University of Bristol, Centre for Exercise, Nutrition and Health Sciences<br>Emm-Collison, Lydia; University of Bristol, Centre for Exercise, Nutrition and Health Sciences<br>Sebire, Simon; University of Bristol, Centre for Exercise, Nutrition and Health Sciences<br>Thompson, Janice; The University of Birmingham, School of Sport & Exercise Sciences<br>Jago, Russ; University of Bristol, Centre for Exercise, Nutrition and Health Sciences
**Primary Subject Heading**:	Public health
Secondary Subject Heading:	Paediatrics
Keywords:	screen-viewing, PAEDIATRICS, TV, computer games, cohort

SCHOLARONE™
Manuscripts

Associations between socio-economic position and changes in children's screen-viewing between ages 6 and 9: a longitudinal study

Ruth Salway[1,*], Lydia Emm-Collison[1], Simon J. Sebire[1], Janice L. Thompson[2] and Russ Jago[1]

[1] Centre for Exercise, Nutrition & Health Sciences, School for Policy Studies, University of Bristol, 8 Priory Road, Bristol, BS8 1TZ, UK.

[2] School of Sport, Exercise and Rehabilitation Sciences, University of Birmingham, Birmingham, B15 2TT, UK

[*] **Corresponding author:** Dr Ruth Salway, Centre for Exercise, Nutrition & Health Sciences, School for Policy Studies, University of Bristol, 8 Priory Road, Bristol, BS8 1TZ, UK.

Email: ruth.salway@bristol.ac.uk; Telephone: +44 (0)117 3310463

Word count: **Manuscript** = 3475 words **Abstract** = 249 words (max 300)

For peer review only

For peer review only - http://bmjopen.bmj.com/site/about/guidelines.xhtml

ABSTRACT

Objectives: To explore socio-economic differences in screen-viewing at age 6 and 9, and how these are related to different media use.

Design: Longitudinal cohort study.

Setting: Children recruited from 57 state-funded primary schools in Southwest England, UK.

Participants: 1299 children at age 5-6, 1223 children at age 8-9, including 685 children at both timepoints.

Outcome measures: Children's total screen-viewing time (parent-reported) and time spent using multiple screen devices simultaneously (multiscreen viewing), for weekdays and weekends.

Methods: Negative binomial regression was used to model associations between socio-economic variables (highest household education and area deprivation) and total screen-viewing at age 6 and the change from age 6 to 9. We additionally adjusted for child characteristics, parental influences and media devices in the home. Multiscreen viewing was analysed separately.

Results: Household education was associated with children's screen-viewing at age 6 and with the change in screen-viewing between ages 6 and 9, with higher screen-viewing in lower socio-economic groups. The differences at age 6 were explained by the presence of games consoles, parental limits on screen-viewing, and average parent screen-viewing. Household education differences in the change in screen-viewing between ages 6 and 9 remained.

Conclusions: Children's screen-viewing patterns differ by parental education with higher levels of viewing among children living in households with lower educational qualifications. These differences are already present at age 6, and continue at age 9. Strategies to manage child sedentary time, and particularly screen-viewing, may need to take account of the socio-economic differences and target strategies to specific groups.

For peer review only - http://bmjopen.bmj.com/site/about/guidelines.xhtml

Keywords: screen viewing, TV, computer games, cohort, children

For peer review only

For peer review only - http://bmjopen.bmj.com/site/about/guidelines.xhtml

ARTICLE SUMMARY

Strengths and limitations of this study

Strengths

- Longitudinal study screen viewing on both weekdays and weekends at two time points.
- Time frame covers an important period of change in child viewing behaviours.
- Data collection period coincided with a period of rapid change in screen-viewing technology which has facilitated the presentation of contemporary data.

Limitations

- We did not explicitly ask parents about tablet or smartphone use and so have not been able to capture this directly.
- A change in the way education was recorded may result in misclassification in a small number of situations where household composition has changed or where people have obtained additional qualifications.

For peer review only

INTRODUCTION

Sedentary behaviour has been associated with increased risk of obesity and elevated levels of cardio-metabolic risk factors among children and adolescents[1, 2]. Screen-viewing is a very common form of sedentary behaviour among children and young people[3], and higher screen-viewing has been associated with higher body mass index, poorer psychological well-being and poorer academic achievement among children[4-6]. The American Academy of Pediatrics recommends restricting children's screen-viewing, although it does not specify a limit[7]. An international study estimated that 11 year old boys engaged in an average of 5.3 h/day of screen-viewing on weekdays in 2010 (4.4 h/day for girls) with higher estimates at weekends[8]. The paper therefore shows that by the end of primary school children are engaging in high levels of screen viewing. Sedentary behaviour tracks at moderate levels from childhood into adolescence[9], with TV viewing tracking from childhood to adulthood[10]. Moreover, screen-viewing has increased in children by around 1.3h/day on weekdays (2.0 h/day on weekends) between 2002 and 2010 [8]. Thus, screen-viewing is an increasingly common behaviour among children and with levels increasing as children age. We therefore need to understand patterns of behaviour develop during childhood, and particularly at the start of primary school when patterns of behaviour are established. As such, information on levels of screen-viewing and how they change during early school years is required.

Much of the current literature on screen-viewing focuses on TV viewing, but screen-viewing has evolved with technology to include TV viewing, computer games, tablets, mobile phones and multiscreen viewing (in which children use two or more devices at the same time)[11] , and become more diverse, for example encompassing on-demand TV and online gaming[3]. Thus, to try and change behaviour there is a need to understand what devices children are using and the types of viewing in which children engage on different days of the week.

For peer review only - http://bmjopen.bmj.com/site/about/guidelines.xhtml

Socio-economic position has been associated with poorer health outcomes among children and young people. It is important to note, however that different indicators of socio-economic position, such as education, income and deprivation, measure different, often related aspects, although they are strongly correlated[12, 13]. For example, in England at the end of primary school (age 11) in 2018 the prevalence of child obesity in the most deprived areas is more than double that of those in the least deprived neighbourhoods (27% vs. 12%)[14], and TV viewing has been identified as a mediator for this association[15]. A number of studies have found an inverse relationship between SES and sedentary time[16], sedentary behaviours[17, 18] and screen-viewing specifically[19, 20] with children's daily screen time varying between 1.7 and 2.4 hours/day for high and low SES families respectively[19]. Sedentary behaviour increases as children age[18], but as much of the evidence is cross-sectional it is not clear whether different SES groups change at the same rate. Longitudinal evidence between ages 2 and 9 suggests that while TV viewing increases similarly for different educational subgroups, there may be different trajectories for different household income groups[21]. It is also not clear whether these SES differences are due to differences in other factors or not. Child obesity, ownership of different media devices, parental screen-viewing and parental limits on screen-viewing have all been found to affect children's screen-viewing and differ between SES groups [1, 19, 22-29]. There is thus a need to better understand SES differences, and especially how these change over time.

The aim of this paper was to explore socio-economic differences in screen-viewing at age 6, and the change in screen-viewing between ages 6 and 9, and whether any differences can be explained by other factors such as different media devices and parental influences. Developing knowledge in this area will support the creation of targeted behaviour change programmes to reduce screen-viewing.

METHODS

For peer review only - http://bmjopen.bmj.com/site/about/guidelines.xhtml

B-PROACT1V is a longitudinal study that aimed to examine the physical activity and sedentary behaviours of primary school children aged 5-11 years, and their parents (described in detail elsewhere [30-32]). The study received ethical approval from the School of Policy Studies Ethics Committee at the University of Bristol, UK, and written parental consent was received for all participants [33] (Reference B-Proact1V study, approval 11th November 2011.) In Phase 1, all children in Year 1 of primary school (aged 5-6 years) from 57 schools in and around Bristol were invited to participate, with data collected between January 2012 and July 2013. In Phase 2, when the children were in Year 4 (aged 8-9 years), all schools from Phase 1 were invited to participate, with 47 schools agreeing. All children were eligible regardless of whether they had participated in Phase 1, and data collection took place between March 2015 and July 2016. Data were collected for 1299 children in Year 1 and 1223 children in Year 4, with 685 children included in both phases. Self-identified 'first' parents completed a questionnaire about personal and family characteristics while 'second' parents completed a shorter questionnaire. This paper uses data from the first parent questionnaires: 1085 (84%) from Year 1, 997 (82%) from Year 4 and 509 (74%) at both time points.

Screen-viewing data

In both years, the first parent was asked about the number of hours their child typically spent engaging in specific screen-viewing behaviours on weekdays and at weekends: TV, computer, games consoles. Additionally, both parents were asked about their own screen-viewing. These responses were recorded as either 'None' or in hourly categories from '0-1 hours' up to '4 hours or more', and recoded based on midpoints to give the average number of minutes spent in each type of screen-viewing on weekdays and weekends. These were summed to form the average total number of minutes spent on any type of screen-viewing. Where two parents completed questionnaires, parental screen-viewing was taken as the average (approximately 27% of respondents in each year). At the second phase, parents were also asked about the time they and their child spent multiscreen-viewing (i.e. using two or more devices at once), with responses as above recoded to midpoints [11].

For peer review only - http://bmjopen.bmj.com/site/about/guidelines.xhtml

Socio-economic data

At age 6, the first parent was asked their highest educational qualification, while at age 9, they were asked the highest education qualification of anyone in the household. We combined these to form the highest household educational qualification recorded at either time with categories 'Up to GCSE (General Certificate of Secondary Education) or equivalent' (qualification at age 16), 'A level/National Vocational Qualification or equivalent' (qualification at age 18), 'University Degree or equivalent' and 'Higher Degree (MSc/PhD) or equivalent'. In addition, Index of Multiple Deprivation (IMD) scores, based on the English Indices of Deprivation (http://data.gov.uk/dataset/index-of-multiple-deprivation), were assigned to each child based on their reported home postcode, with higher IMD scores indicating a greater level of deprivation. Household education captures individual long-term socio-economic position, such as knowledge, and also provides an indirect indication of income, while IMD captures the socio-economic conditions of the area in which they live.

Other measurements

Child gender was reported by the first parent. Child height and weight were recorded to the nearest 0.1 cm and 0.1 kg, respectively, by trained fieldworkers at each time point. Body mass index (BMI) was calculated and converted to an age- and sex-specific standard deviation score based on UK reference curves[34, 35]. The first parent was asked whether they limited the time their child spent engaging in three different types of screen-viewing (TV, computer and video games) with responses from 1 'Strongly disagree' to 4 'Strongly agree'. The average of these was used to capture parental limits on screen-viewing. We also asked about the number of media devices in the home: TVs, computers (desktop or laptop), tablet computers and games consoles (including handheld consoles).

Patient and Public Involvement

For peer review only

For peer review only - http://bmjopen.bmj.com/site/about/guidelines.xhtml

The research questions for this study emerged from research that was conducted with 1078 Year 1 children. A sub-set of 53 parents of the children who took part in the Year 1 study participated in interviews to help guide future research. Participants provided verbal assent (with parental consent) to join the study, and summaries of project findings are sent to all participants via study schools.

Statistical Analysis

Descriptive summaries of children's screen-viewing on weekdays and weekends and other participant characteristics were produced by household education group for age 6 and age 9. To aid interpretation, the screen-viewing variables are reported in summaries as continuous variables. However, the underlying variables are discrete and so in the main analyses we used negative binomial regression models (similar to Poisson models but suitable for over-dispersed count data) to model weekday and weekend screen-viewing separately. We considered four main models. Model 1 explored cross-sectional associations between socioeconomic position (household education and IMD) and total minutes of screen-viewing at age 6. Model 2 additionally adjusted for possible mediators and confounders: child gender, child BMI, number of devices in the home (more than one TV, and presence of any computers, tablets or games consoles), parental screen-viewing and parental behaviour on limiting screen-viewing. Models 3 (unadjusted) and 4 (adjusted for covariates) explored the longitudinal change between ages 6 and 9; that is, associations between socioeconomic position and total minutes of screen-viewing at age 9, adjusting for baseline screen-viewing at age 6. Finally, we also explored the association between socioeconomic position and multiscreen viewing as a separate outcome. Robust standard errors were used to account for clustering of children within schools, and all analyses are based on complete cases. Model results are presented as screen time ratios (rate ratios), defined as the exponent of the model coefficients. An increase of one unit in a given predictor variable is associated with a multiplicative increase in screen-viewing, holding the

For peer review only

For peer review only - http://bmjopen.bmj.com/site/about/guidelines.xhtml

other predictor variables in the model constant. Model assumptions were checked via model diagnostics, and all analysis was done in Stata v15.

RESULTS

Children's screen-viewing differed between households with differing levels of education at both ages 6 and 9, with higher screen-viewing in lower qualified households, decreasing as education increased, with 35-50 minutes difference between the highest and lowest household education qualifications (Figure 1). Time spent screen-viewing was higher at age 9 than age 6, and higher at weekends than during the week among all education groups, with time spent watching TV and playing games consoles nearly doubling at weekends (Table S1 and S2). Time spent engaged in multiscreen viewing was also higher for households with lower levels of qualification, with children in households where GCSEs were the highest qualification spending over twice as much time in multiscreen viewing as those where the highest qualification was a postgraduate degree, on both weekdays and weekends. Missing data ranged between 0-3% (Table S3).

There were differences in the number and types of media devices in the home by household education (Figure 2). Households where GCSE and A Levels were the highest qualification had more TVs and games consoles in the house, while Higher Degree households had more computers. There were small increases in most devices between age 6 and age 9. The average number of tablet devices was the same across all levels of household education but increased fourfold to an average of 2.2 per household between the two assessment periods. Parental screen-viewing differed with household education by similar amounts to their children, again with higher screen-viewing among those with lower level qualifications. Parental screen-viewing was also higher at the second time point, especially among the Higher Degree educated households, and differences with education were smaller.

For peer review only

Table 1 shows the associations between socioeconomic position and total screen-viewing at age 6, in terms of ratios for screen time. For example, a factor of 0.79 for Degree-level household education (Table 1, weekday) means that screen-viewing time decreased by 21% for children in households where a University degree or equivalent was the highest qualification, compared to children in households where the highest qualifications were GCSEs. In the unadjusted model (Model 1) there were differences in screen-viewing by household education for both weekdays and weekends, with lower screen-viewing for children in households with higher education. The GCSE and A level education groups were similar, but children in households with a Degree or Higher degree engaged in 21-27% less screen-viewing. There was no association with IMD. The adjusted model (Model 2) adjusts for possible mediators: number of devices, child BMI, parental screen-viewing and parental limits on screen-viewing all measured at age 6. These factors accounted for household education differences on both weekdays and weekends. Children's screen-viewing was higher in households with games consoles, and lower when parents were more likely to limit screen-viewing (Table S4). There were weak associations with parental screen-viewing and child BMI.

Models 3 (unadjusted) and 4 (adjusted) examined the change in screen-viewing between ages 6 and 9 (Table 2). In Model 3, adjusting for baseline screen-viewing, there were differences in screen-viewing by household education, with larger increases in screen-viewing for children from A level and Degree households (13% and 6% respectively) and 16% less increase in Higher Degree households, compared to GCSE households in the week. Similar patterns were seen at weekends, with the A level and Degree groups increasing more than the GCSE and Higher Degree groups. There was no association with IMD. In Model 4, when adjusting for media devices in the home, child BMI and parental influences at age 9, household education differences still remain, with larger increases for the A level and Degree groups. An increase in screen-viewing was strongly associated with the presence of games consoles and multiple TVs (weekdays only), with weaker associations

For peer review only

For peer review only - http://bmjopen.bmj.com/site/about/guidelines.xhtml

with parental screen-viewing (Table S5). Presence of tablets was associated with a decrease in screen-viewing, as was parental limits on screen-viewing in the week, but not at weekends.

Multiscreen viewing was associated with both IMD but not with household education, with children's multiscreen viewing higher in families living in more deprived areas (Table S6 and Figure S1). When adjusting for other factors, the association with deprivation remained, with multiscreen viewing 26-27% higher for every standard deviation increase in IMD score. Higher amounts of multiscreen viewing were associated with higher levels of parental multiscreen viewing and the presence of tablet devices and multiple TVs in the week, and lower levels of multiscreen viewing were associated with increased parental limits on screen-viewing.

DISCUSSION

The data presented in this paper have shown an association between children's screen-viewing and highest household education level, with higher screen-viewing in lower socio-economic groups. These differences were already present at age 6 and continued to be evident at age 9. Socio-economic differences at age 6 were accounted for by other factors, especially parental factors such as their own screen-viewing and their behaviour in limiting their children's screen-viewing. However, while the time children spent engaged in screen-viewing increased in all households between ages 6 and 9, regardless of education, they increased by different amounts depending on household education. Adjusting for baseline screen-viewing at age 6, screen-viewing increased most over the time period for children in households where the highest qualifications were A Levels or a university degree compared to GCSE, with the smallest increase among Higher Degree households. In A-level educated households, this reflected a "catching-up" with the level of screen-viewing seen in GCSE-educated households. These findings imply socio-economic differences in both amount of screen-viewing, and the way in which screen-viewing patterns develop. These differences were related to

For peer review only - http://bmjopen.bmj.com/site/about/guidelines.xhtml

household education rather than area-level deprivation, suggesting that it is long-term socio-economic aspects such as parental knowledge, ability to engage and communicate with services, and possibly income that are important, rather than the socio-economic conditions of the area, such as availability of local resources. In contrast, multiscreen viewing is associated with area deprivation rather than education and so could represent a proxy for short-term current income or possibly a neighbourhood effect of what is considered to be 'typical' screen-viewing behaviour. As such, strategies to manage child sedentary time, and particularly different types of screen-viewing, may need to take account of the socio-economic differences and target strategies to specific groups.

Ownership of media devices such as TVs, phones and games consoles, have previously been found to be associated with higher levels of screen-viewing, and also inversely with socio-economic status [24, 28]. We found that the number of media devices in the home differed by household education, with more TVs and games consoles in households with GCSE and A level qualifications, and more computers in households with a university or higher degree. While the number of devices changed overall between the two assessment points, most notably with a large increase in tablet devices and a slight decrease in the number of computers, socio-economic differences were small and did not account for the observed differences in total screen-viewing between household education. We found that higher screen-viewing at age 6 was associated with games consoles at the weekends only, but not with multiple TVs or computers, and larger increases in screen-viewing between ages 6 and 9 were associated with games consoles and multiple TVs in the week. However, we also saw tablet ownership associated with smaller increases in screen-viewing between ages 6 and 9. This suggests that the relationship between different devices and screen-viewing is complex, and may be changing over time. Thus, we extend previous findings to show that while access to devices may contribute to baseline screen-viewing, it does not entirely explain socio-economic differences in the increase in screen-viewing between ages 6 and 9.

For peer review only - http://bmjopen.bmj.com/site/about/guidelines.xhtml

There were weak associations between parent and child screen-viewing, with every 30 minutes of parental screen-viewing associated with a 2% increase in child screen-viewing. Parental limits on screen-time were strongly associated with lower screen-viewing at age 6 and less time spent multi-screen-viewing at age 9, but associations were stronger for weekdays than weekends and limiting screen time was associated with changes in screen-viewing between ages 6 and 9 on weekdays only. Interestingly, analysis of interviews conducted with a sub-sample of the parents in the study showed that many parents are uneasy about managing non-TV screen-time and feel that they struggle to keep up with rapid technological change[36]. Collectively, these findings may suggest that there is a need to help parents to identify effective ways to manage constantly adapting forms of viewing.

Tablet ownership did not vary with household education but increased greatly overall between ages 6 and 9. A limitation of this project is that we did not assess tablet or smartphone use as a specific behaviour in the questionnaire. This may under-estimate total screen-viewing, especially for those who are heavy users of these devices, and may explain the observed negative association between screen-viewing and tablet ownership. We also note that tablet ownership is strongly associated with higher levels of multiscreen-viewing on weekdays. The widespread ownership of tablets is particularly important for understanding the complexity of screen-viewing, as watching TV, playing games and using the internet can all be done via a tablet and, as such, identifying the behaviour engaged in while using a tablet is challenging. Thus, it may be that in the future there is a specific need to assess tablet time, and to differentiate the various ways in which tablets are used. For example, applications that allow parents and children to agree targets to limit the time spent on games and other non-educational activities, could also be used as a feedback and monitoring device.

For peer review only - http://bmjopen.bmj.com/site/about/guidelines.xhtml

The data presented in this paper add to the previous evidence base which has shown that socio-economic position is associated with sedentary time and screen-viewing. Previous work within this area has focussed on cross-sectional associations between TV time and various indicators of socio-economic position [15-20]. Collectively this body of evidence identifies an inverse association with children residing in more socioeconomically disadvantaged households engaging in more TV viewing. The findings from this paper greatly extend this work by showing that these patterns are existent cross-sectionally and prospectively. The prospective associations, are however, particularly informative as they show that these differences are evident at age 6 and continue as child ages. These findings suggest that we need a range of options to help families to manage their screen-time and that these made need to be tailored to specific socio-economic groups. For example, for families from lower socio-economic groups there is likely to be a need for programs that focus on stopping the development of screen-viewing behaviours in early childhood. This could be parental education programs that are combined with behaviour change techniques, while for older children from all socio-economic groups there may need to be more of a focus on family-focussed management information where children and parents work together to set family viewing goals [37]. It is unlikely that a single strategy will work on its own[38], and as such there is need for multiple strategies across childhood that adapt to the age and viewing habits of the child and the family.

For peer review only

The major strengths of this study are the information on a variety of different types of screen-viewing at two time points which span an important period of change in child viewing behaviours. The data collection period (2012-16) coincided with a period of rapid change in screen-viewing technology which has facilitated the presentation of contemporary data. There are, however, several limitations that need to be considered. Firstly, we did not explicitly ask parents about tablet or smartphone use. As data collection covers a time when screen use is changing rapidly, (for example, global tablet sales increased from 116 million in 2012[39] to 207 million in 2015[40]) this is an

For peer review only - http://bmjopen.bmj.com/site/about/guidelines.xhtml

important aspect we have not been able to capture directly. Secondly, the differences in the education variable between Year 1 (first parent's education) and Year 4 (household education) may result in inaccuracies in situations where household composition has changed or where people have obtained additional qualifications between the two time points, although we believe that the number of these cases is likely to be small. Children's screen-viewing was parent-reported, and amounts of multiscreen-viewing, in particular, may not be well-captured, although general patterns might be more robust. This is a longitudinal study, and so some children were lost to follow-up, although the majority were through non-participating schools. In addition, as we have included socioeconomic position variables directly, our models will not biased due to differential follow-up rates by socioeconomic status. Finally, as parental screen-viewing increased between 2012 and 2015 this may indicate a general change in viewing patterns, and the increases reported between the two age periods could reflect secular changes as opposed to age-related differences. As such, it would be important to identify if comparable patterns are evident in other datasets.

Conclusion

Children's screen-viewing patterns differ by parental education with higher levels of viewing among children living in households with lower levels of education. These differences are already present at age 6, and continue at age 9, even when accounting for differences in baseline screen-viewing and device ownership. Socio-economic differences narrow with age as children in households with higher qualifications gain greater access to screen-viewing devices. Strategies to manage child sedentary time, and particularly screen-viewing, may need to take account of the socio-economic differences and target strategies to specific groups. For example, specific early intervention strategies to reduce screen-viewing in children from lower socio-economic groups are likely to help stop high levels of screen-viewing from developing and reduce inequalities.

For peer review only - http://bmjopen.bmj.com/site/about/guidelines.xhtml

ACKNOWLEDGEMENTS

We would like to thank all of the families and schools that have taken part in the B-PROACT1V project. We would also like to thank all current and previous members of the research team who are not authors on this paper.

COMPETING INTERESTS

We have no competing interest to report.

FUNDING

This work was supported by the British Heart Foundation (ref PG/11/51/28986 and SP 14/4/31123). The funder had no involvement in data analysis, data interpretation or writing of the paper.

AUTHOR CONTRIBUTIONS

RJ, SJS and JLT were involved in the design of this study and in seeking funding for it. The paper was conceived by RJ and RS, and RS performed all analyses. RS and RJ wrote the first draft of the paper and RJ coordinated contributions from other authors. All authors (RJ, SJS, JLT, LEC) made critical comments on drafts of the paper.

DATA SHARING STATEMENT

The datasets generated during the current study are not publicly available due as the project is ongoing and data are not ready for archiving. We will consider reasonable requests for access to the data once the project is complete in 2019.

REFERENCES

1. Ekelund U, Brage S, Froberg K, Harro M, Anderssen S, Sardinha LB, Riddoch CJ, Andersen LB: **TV Viewing and Physical Activity Are Independently Associated with Metabolic Risk in Children: The European Youth Heart Study.** *PLoS Med* 2006, **3:**e488.

For peer review only - http://bmjopen.bmj.com/site/about/guidelines.xhtml

2. Ekelund U, Luan J, Sherar LB, Esliger DW, Griew P, Cooper A, International Children's Accelerometry Database C: **Moderate to vigorous physical activity and sedentary time and cardiometabolic risk factors in children and adolescents.** *JAMA* 2012, **307:**704-712.

3. Saunders TJ, Vallance JK: **Screen Time and Health Indicators Among Children and Youth: Current Evidence, Limitations and Future Directions.** *Appl Health Econ Health Policy* 2017, **15:**323-331.

4. Corder K, Atkin AJ, Bamber DJ, Brage S, Dunn VJ, Ekelund U, Owens M, van Sluijs EM, Goodyer IM: **Revising on the run or studying on the sofa: prospective associations between physical activity, sedentary behaviour, and exam results in British adolescents.** *Int J Behav Nutr Phys Act* 2015, **12:**106.

5. Page AS, Cooper AR, Griew P, Jago R: **Children's Screen Viewing is Related to Psychological Difficulties Irrespective of Physical Activity.** *Pediatrics* 2010, **126:**e1011-1017.

6. Jago R, Baranowski T, Baranowski JC, Thompson D, Greaves KA: **BMI from 3-6 y of age is predicted by TV viewing and physical activity, not diet.** *Int J Obes (Lond)* 2005, **29:**557-564.

7. American Academy of Pediatrics: **Media Use in School-Aged Children and Adolescents.** *Pediatrics* 2016, **138:**e20162591.

8. Bucksch J, Sigmundova D, Hamrik Z, Troped PJ, Melkevik O, Ahluwalia N, Borraccino A, Tynjala J, Kalman M, Inchley J: **International Trends in Adolescent Screen-Time Behaviors From 2002 to 2010.** *J Adolesc Health* 2016, **58:**417-425.

9. Biddle SJ, Pearson N, Ross GM, Braithwaite R: **Tracking of sedentary behaviours of young people: a systematic review.** *Prev Med* 2010, **51:**345-351.

10. Smith L, Gardner B, M. H: **Childhood correlates of adult TV viewing time: a 32-year follow-up of the 1970 British Cohort Study.** *J Epidemiol Community Health* 2015, **69:**309-313.

11. Jago R, Sebire SJ, Gorely T, Cillero IH, Biddle SJ: **"I'm on it 24/7 at the moment": a qualitative examination of multi-screen viewing behaviours among UK 10-11 year olds.** *Int J Behav Nutr Phys Act* 2011, **8:**85.

12. Galobardes B, Shaw M, Lawlor DA, Lynch JW, Davey Smith G: **Indicators of socioeconomic position (part 1).** *J Epidemiol Community Health* 2006, **60:**7-12.

13. Galobardes B, Shaw M, Lawlor DA, Lynch JW, Davey Smith G: **Indicators of socioeconomic position (part 2).** *J Epidemiol Community Health* 2006, **60:**95-101.

14. NHS Digital: **National Child Measurement Programme, England 2017-2018 school year.** UK; 2018.

15. Morgenstern M, Sargent JD, Hanewinkel R: **Relation between socioeconomic status and body mass index: evidence of an indirect path via television use.** *Arch Pediatr Adolesc Med* 2009, **163:**731-738.

16. Sherar LB, Griffin TP, Ekelund U, Cooper AR, Esliger DW, van Sluijs EM, Bo Andersen L, Cardon G, Davey R, Froberg K, et al: **Association between maternal education and objectively measured physical activity and sedentary time in adolescents.** *J Epidemiol Community Health* 2016, **70:**541-548.

17. Coombs N, Shelton N, Rowlands A, Stamatakis E: **Children's and adolescents' sedentary behaviour in relation to socioeconomic position.** *J Epidemiol Community Health* 2013, **67:**868-874.

18. Moraeus L, Lissner L, Olsson L, Sjoberg A: **Age and time effects on children's lifestyle and overweight in Sweden.** *BMC Public Health* 2015, **15:**355.

19. Tandon P, Chuan Zhou C, Sallis J, Cain K, Frank L, Saelens B: **Home environment relationships with children’s physical activity, sedentary time, and screen time by socioeconomic status.** *Int J Behav Nutr Phys Act* 2012, **9:**88.

For peer review only

20. Drenowatz C, Eisenmann JC, Pfeiffer KA, Welk G, Heelan K, Gentile D, Walsh D: **Influence of socio-economic status on habitual physical activity and sedentary behavior in 8- to 11-year old children.** *BMC Public Health* 2010, **10:**214.

21. Yang-Huang J, van Grieken A, Moll HA, Jaddoe VWV, Wijtzes AI, Raat H: **Socioeconomic differences in children's television viewing trajectory: A population-based prospective cohort study.** *PLoS One* 2017, **12:**e0188363.

22. Jago R, Page A, Froberg K, Sardinha L, Klasson-Heggebo L, Andersen L: **Screen-viewing and the home TV environment: The European Youth Heart Study.** *Prev Med* 2008, **47:**525 - 529.

23. Jago R, Davison K, Thompson JL, Page AS, Brockman R, Fox KR: **Parental sedentary restriction, maternal parenting style and TV viewing among 10-11 year olds** *Pediatrics* 2011, **128:**e572-e578.

24. Jago R, Stamatakis E, Gama A, Carvalhal IM, Nogueira H, Rosado V, Padez C: **Parent and child screen-viewing time and home media environment.** *Am J Prev Med* 2012, **43:**150-158.

25. Jago R, Sebire SJ, Edwards MJ, Thompson JL: **Parental TV viewing, parental self-efficacy, media equipment and TV viewing among preschool children.** *Eur J Pediatr* 2013, **172:**1543-1545.

26. Jago R, Thompson JL, Sebire SJ, Wood L, Pool L, Zahra J, Lawlor DA: **Cross-sectional associations between the screen-time of parents and young children: differences by parent and child gender and day of the week.** *International Journal of Behavioral Nutrition and Physical Activity* 2014, **11**.

27. Lauricella AR, Wartella E, Rideout VJ: **Young children's screen time: The complex role of parent and child factors.** *Journal of Applied Developmental Psychology* 2015, **36:**11-17.

28. Dumuid D, Olds TS, Lewis LK, Maher C: **Does home equipment contribute to socioeconomic gradients in Australian children's physical activity, sedentary time and screen time?** *BMC Public Health* 2016, **16:**736.

29. Gebremariam MK, Altenburg TM, Lakerveld J, Andersen LF, Stronks K, Chinapaw MJ, Lien N: **Associations between socioeconomic position and correlates of sedentary behaviour among youth: a systematic review.** *Obes Rev* 2015, **16:**988-1000.

30. Jago R, Sebire SJ, Wood L, Pool L, Zahra J, Thompson JL, Lawlor DA: **Associations between objectively assessed child and parental physical activity: a cross-sectional study of families with 5-6 year old children.** *BMC Public Health* 2014, **14:**655.

31. Jago R, Thompson JL, Sebire SJ, Wood L, Pool L, Zahra J, Lawlor DA: **Cross-sectional associations between the screen-time of parents and young children: differences by parent and child gender and day of the week.** *Int J Behav Nutr Phys Act* 2014, **11:**54.

32. Jago R, Solomon-Moore E, Macdonald-Wallis C, Sebire SJ, Thompson JL, Lawlor DA: **Change in children's physical activity and sedentary time between Year 1 and Year 4 of primary school in the B-PROACT1V cohort.** *Int J Behav Nutr Phys Act* 2017, **14:**33.

33. Jago R, Bailey R: **Ethics and paediatric exercise science: Issues and making a submission to a local ethics and research committee.** *Journal of Sport Sciences* 2001, **19:**527-535.

34. Cole TJ, Freeman JV, Preece MA: **Body mass index reference curves for the UK, 1990.** *Arch Dis Child* 1995, **73:**25-29.

35. Cole TJ, Bellizzi MC, Flegal KM, H. DW: **Establishing a standard definition for child overweight and obesity worldwide: international survey.** *BMJ Open* 2000, **320:**1240-1243.

36. Jago R, Solomon-Moore E, Toumpakari Z, Lawlor DA, Thompson JL, Sebire SJ: **Parents' perspectives of change in child physical activity & screen-viewing between Y1 (5-6) & Y4 (8-9) of primary school: implications for behaviour change.** *BMC Public Health* 2018, **18:**520.

For peer review only - http://bmjopen.bmj.com/site/about/guidelines.xhtml

37. Jago R, Sebire SJ, Turner KM, Bentley GF, Goodred JK, Fox KR, Stewart-Brown S, Lucas PJ: **Feasibility trial evaluation of a physical activity and screen-viewing course for parents of 6 to 8 year-old children: Teamplay.** *Int J Behav Nutr Phys Act* 2013, **10:**31.

38. Rutter H, Savona N, Glonti K, Bibby J, Cummins S, Finegood DT, Greaves F, Harper L, Hawe P, Moore L, et al: **The need for a complex systems model of evidence for public health.** *The Lancet* 2017, **390:**2602-2604.

39. **Gartner Says Worldwide Tablet Sales Grew 68 Percent in 2013, With Android Capturing 62 Percent of the Market** [https://www.gartner.com/newsroom/id/2674215]

40. **Worldwide Shipments of Slate Tablets Continue to Decline While Detachable Tablets Climb to New High, According to IDC** [https://web.archive.org/web/20160204014026/http://www.idc.com/getdoc.jsp?containerId=prUS40990116]

For peer review only

Table 1 – Cross-sectional associations between socioeconomic position and screen-viewing at age 6

	Model 1: unadjusted			Model 2: adjusted[1]		
	Ratio	95% CI	p-value	Ratio	95% CI	p-value
**WEEKDAY**		N=1043			N=953	
Household education						
Up to GCSE	1	(Reference)		1	(Reference)	
A level	0.94	(0.85, 1.03)		0.98	(0.89, 1.08)	
Degree	0.79	(0.70, 0.90)		0.88	(0.78, 1.00)	
Higher degree	0.73	(0.60, 0.88)	<0.0005[2]	0.88	(0.74, 1.05)	0.054[2]
Deprivation (IMD)[3]	1.04	(1.00, 1.08)	0.067	1.02	(0.98, 1.07)	0.296
**WEEKEND**		N=1038			N=946	
Household education						
Up to GCSE	1	(Reference)		1	(Reference)	
A level	1.00	(0.91, 1.10)		1.03	(0.95, 1.12)	
Degree	0.88	(0.79, 0.98)		1.02	(0.93, 1.13)	
Higher degree	0.77	(0.67, 0.90)	0.001[2]	0.99	(0.87, 1.11)	0.725[2]
Deprivation (IMD)[3]	1.02	(0.98, 1.06)	0.387	1.01	(0.96, 1.06)	0.720

[1] Adjusted for child gender, child BMI, presence of TVs, computers, tablets and games consoles in the household, parental screen-viewing and parental limiting of screen-viewing

[2] P-value: test for differences between education categories

[3] increase per 1 standard deviation (14.0) in IMD: higher values indicate more deprived areas

For peer review only

Table 2 – Longitudinal associations between socioeconomic position and screen-viewing at age 9, adjusting for baseline screen-viewing at age 6

	Model 3: unadjusted			Model 4: adjusted[1]		
	Ratio	95% CI	p-value	Ratio	95% CI	p-value
**WEEKDAY**		N=487			N=476	
Household education						
Up to GCSE	1	(Reference)		1	(Reference)	
A level	1.13	(1.01, 1.26)		1.16	(1.03, 1.32)	
Degree	1.06	(0.92, 1.22)		1.10	(0.96, 1.26)	
Higher degree	0.84	(0.70, 0.99)	0.001[2]	0.90	(0.77, 1.05)	0.008[2]
Deprivation (IMD)[3]	1.06	(1.00, 1.13)	0.053	1.05	(1.00, 1.10)	0.038
**WEEKEND**		N=483			N=469	
Household education						
Up to GCSE	1	(Reference)		1	(Reference)	
A level	1.14	(1.00, 1.30)		1.14	(1.01, 1.28)	
Degree	1.09	(0.96, 1.23)		1.11	(0.99, 1.24)	
Higher degree	0.96	(0.83, 1.10)	0.004[2]	0.98	(0.87, 1.11)	0.006[2]
Deprivation (IMD)[3]	1.02	(0.98, 1.07)	0.346	1.00	(0.95, 1.05)	0.938

[1] Adjusted for child gender, presence of TVs, computers, tablets and games consoles in the household, parental screen-viewing and parental limiting of screen-viewing

[2] P-value: test for differences between education categories

[3] increase per 1 standard deviation (14.0) in IMD: higher values indicate more deprived areas

For peer review only

For peer review only - http://bmjopen.bmj.com/site/about/guidelines.xhtml

FIGURE LEGENDS AND KEYS

Figure 1: Boxplots of children's total minutes screen-viewing by household education at ages 6 and 9, for weekdays (left) and weekends (right).

Figure 2: Average number of devices in the home by household education at ages 6 (left) and 9 (right).

For peer review only

For peer review only - http://bmjopen.bmj.com/site/about/guidelines.xhtml

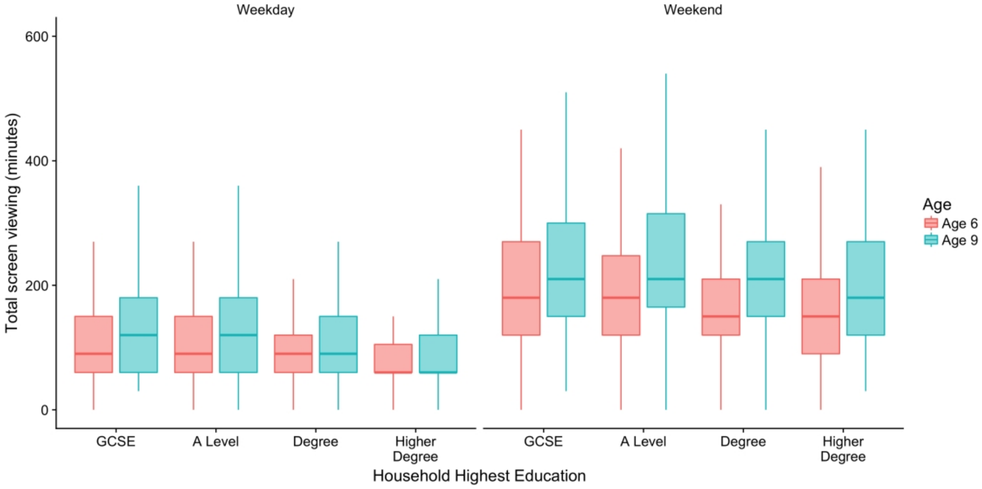

Boxplots of children's total minutes screen-viewing by household education at ages 6 and 9, for weekdays (left) and weekends (right).

1270x635mm (72 x 72 DPI)

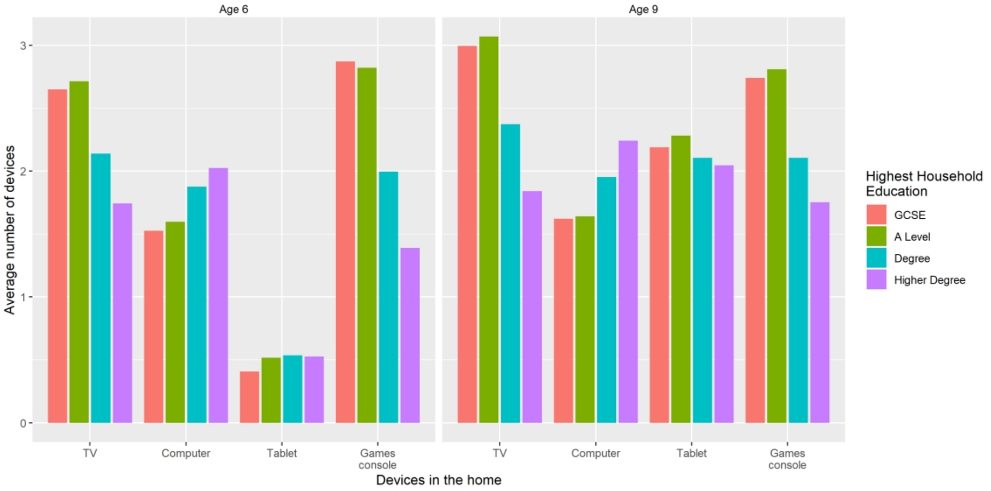

Average number of devices in the home by household education at ages 6 (left) and 9 (right).

SUPPLEMENTARY MATERIAL

ASSOCIATIONS BETWEEN SOCIO-ECONOMIC POSITION AND CHANGES IN CHILDREN'S SCREEN-VIEWING BETWEEN AGES 6 AND 9: A LONGITUDINAL STUDY

Ruth Salway, Lydia Emm-Collison, Simon J. Sebire, Janice L. Thompson and Russell Jago

For peer review only

List of Supplementary Tables

Table S1 – Characteristics at age 6

Table S2 - Characteristics at age 9

Table S3 – Missing values

Table S4 – full details of adjusted model for screen-viewing at age 6

Table S5 – full details of adjusted model for screen-viewing at age 9

Table S6– Associations between socioeconomic position and multi-screen-viewing at age 9

List of Supplementary Figures

Figure S1: Number of minutes spent multi-screen viewing by household education at age 9, for weekdays (left) and weekends (right).

Table S1 – Characteristics at age 6

	Up to GCSE or equiv	A Levels or equiv	Degree or equiv	Higher Degree or equiv	All
	Mean (sd) or %	Mean (sd) or %	Mean (sd) or %	Mean (sd) or %	Mean (sd) or %
WEEKDAY					
Total SV (mins)	120 (74)	111 (61)	93 (60)	85 (70)	102 (66)
TV (mins)	73 (53)	69 (45)	60 (41)	53 (44)	64 (46)
Computer (mins)	25 (28)	23 (27)	20 (22)	23 (33)	22 (26)
Games consoles (mins)	23 (30)	18 (20)	14 (25)	10 (20)	16 (24)
WEEKEND					
Total SV (mins)	196 (118)	193 (107)	169 (94)	150 (102)	178 (105)
TV (mins)	117 (78)	120 (72)	110 (69)	89 (68)	111 (72)
Computer (mins)	37 (44)	34 (38)	32 (33)	37 (39)	34 (37)
Games consoles (mins)	43 (55)	40 (45)	28 (39)	24 (39)	33 (45)
IMD	19.0 (15.5)	16.6 (13.7)	12.2 (10.4)	10.4 (7.4)	14.6 (12.5)
Female	45%	47%	50%	47%	48%
BMI z-score	0.49 (1.04)	0.18 (0.88)	0.16 (0.88)	0.24 (0.94)	0.23 (0.93)
No. devices in home:					
TVs	2.7 (1.3)	2.6 (1.3)	2.2 (1.1)	1.7 (1.1)	2.3 (1.2)
Computers	1.6 (1.1)	1.6 (0.9)	1.9 (1.0)	2.0 (1.1)	1.8 (1.0)
Tablets	0.4 (0.7)	0.5 (0.8)	0.5 (0.7)	0.5 (0.7)	0.5 (0.7)
Games consoles	2.8 (1.8)	2.8 (1.7)	2.0 (1.6)	1.3 (1.3)	2.3 (1.7)
Parent SV (mins)					
Weekday	231 (141)	230 (134)	220 (112)	202 (115)	222 (125)
Weekend	253 (146)	258 (124)	228 (103)	209 (113)	235 (120)
Parental limits on SV[1]	3.3 (0.6)	3.3 (0.6)	3.4 (0.6)	3.5 (0.6)	3.3 (0.6)

[1] higher values indicate more limits on screen viewing (between 1 and 4)

For peer review only

Table S2 - Characteristics at age 9

	Up to GCSE or equiv	A Levels or equiv	Degree or equiv	Higher Degree or equiv	All
	Mean (sd) or %	Mean (sd) or %	Mean (sd) or %	Mean (sd) or %	Mean (sd) or %
WEEKDAY					
Total SV (mins)	139 (94)	133 (78)	113 (75)	88 (59)	118 (78)
TV (mins)	75 (55)	72 (49)	63 (46)	48 (38)	65 (48)
Computer (mins)	37 (46)	34 (37)	31 (37)	29 (31)	32 (37)
Games consoles (mins)	28 (44)	26 (34)	19 (30)	12 (24)	21 (33)
Multi-screen viewing (mins)	29 (46)	24 (40)	16 (33)	11 (25)	19 (37)
WEEKEND					
Total SV (mins)	244 (134)	245 (120)	227 (118)	200 (118)	230 (122)
TV (mins)	127 (72)	126 (66)	125 (63)	104 (62)	122 (65)
Computer (mins)	59 (66)	58 (64)	53 (60)	57 (54)	56 (61)
Games consoles (mins)	58 (68)	62 (63)	49 (62)	39 (54)	52 (62)
Multi-screen viewing (mins)	53 (73)	48 (66)	33 (61)	23 (45)	39 (63)
IMD	20.1 (15.8)	17.6 (15.0)	12.8 (11.9)	12.0 (10.3)	15.1 (13.6)
Female	56%	52%	56%	53%	55%
BMI z-score	0.44 (1.15)	0.28 (1.04)	0.22 (1.06)	0.28 (1.02)	0.31 (1.06)
No. devices in the home					
TVs	2.9 (1.3)	3.1 (1.4)	2.4 (1.3)	1.8 (1.1)	2.6 (1.4)
Computers	1.6 (1.2)	1.7 (1.1)	2.0 (1.2)	2.2 (1.1)	1.9 (1.2)
Tablets	2.1 (1.2)	2.3 (1.3)	2.1 (1.2)	2.0 (1.2)	2.2 (1.3)
Games consoles	2.6 (2.1)	2.8 (1.8)	2.2 (1.6)	1.7 (1.6)	2.3 (1.8)
Parent SV (mins)					
Weekday	248 (134)	245 (128)	248 (134)	253 (144)	248 (134)
Weekend	280 (154)	287 (135)	260 (130)	252 (144)	270 (138)
Parental limits on SV[1]	3.1 (0.7)	3.2 (0.6)	3.4 (0.6)	3.4 (0.5)	3.3 (0.6)

[1] higher values indicate more limits on screen viewing (between 1 and 4)

For peer review only

Table S3 – Missing values

	Age 6		Age 9		Data at both time points: Age 6		Data at both time points: Age 9	
	N	% missing	N	% missing	N	% missing	N	% missing
Total	1085		997		509		509	
Household education	1071	1%	992	0.2%	509	0%	509	0%
IMD	1069	1%	983	1%	501	2%	499	2%
**WEEKDAY**								
Total SV (mins)	1069	1%	979	2%	504	1%	502	1%
Multi-SV (mins)			981	2%			503	1%
Parent SV (mins)	1075	1%	981	2%	505	1%	503	1%
Parent multi-SV (mins)			983	1%			503	1%
**WEEKEND**								
Total SV (mins)	1065	2%	978	2%	500	2%	501	2%
Multi-SV (mins)			981	2%			503	1%
Parent SV (mins)	1068	2%	979	2%	505	1%	501	2%
Parent multi-SV (mins)			983	1%			503	1%
Gender	1085	0%	997	0%	509	0%	509	0%
BMI z-score	1072	1%	993	0.4%	502	1%	508	0.2%
No. TVs	1073	1%	982	2%	507	0.4%	503	1%
No. Computers	1058	2%	982	2%	504	1%	503	1%
No. Tablets	1062	2%	981	2%	504	1%	503	1%
No. games consoles	1056	3%	981	2%	500	2%	503	1%
Parental limits on SV	1064	2%	971	3%	495	3%	497	2%

For peer review only

Table S4 – full details of adjusted model for screen-viewing at age 6

	WEEKDAY			WEEKEND		
	Ratio	95% CI	p-value	Ratio	95% CI	p-value
Household education						
Up to GCSE	1	(Reference)		1	(Reference)	
A level	0.98	(0.89, 1.08)		1.03	(0.95, 1.12)	
Degree	0.88	(0.78, 1.00)		1.02	(0.93, 1.13)	
Higher degree	0.88	(0.74, 1.05)	0.054	0.99	(0.87, 1.11)	0.725
Deprivation (IMD)[1]	1.02	(0.98, 1.07)	0.296	1.01	(0.96, 1.06)	0.720
>1 TV	0.99	(0.90, 1.10)	0.924	1.04	(0.95, 1.13)	0.394
Any computers	0.94	(0.74, 1.21)	0.645	0.86	(0.73, 1.02)	0.080
Any tablets	1.02	(0.94, 1.10)	0.652	1.01	(0.94, 1.08)	0.811
Any games consoles	1.15	(1.00, 1.32)	0.043	1.42	(1.26, 1.60)	<0.0005
Child gender	0.87	(0.81, 0.94)	<0.0005	0.82	(0.78, 0.87)	<0.0005
z-BMI score	1.07	(1.02, 1.12)	0.003	1.04	(1.01, 1.07)	0.021
parent SV (mins)[2]	1.04	(1.03, 1.05)	<0.0005	1.05	(1.04, 1.07)	<0.0005
Parental limits on SV	0.77	(0.72, 0.83)	<0.0005	0.85	(0.81, 0.90)	<0.0005

[1] increase per 1 standard deviation (14.0) in IMD: higher values indicate more deprived areas

[2] increase per 30 minutes screen-viewing

For peer review only

Table S5 – full details of adjusted model for screen-viewing at age 9

	WEEKDAY			WEEKEND		
	Ratio	95% CI	p-value	Ratio	95% CI	p-value
Household education						
Up to GCSE	1	(Reference)		1	(Reference)	
A level	1.16	(1.03, 1.32)		1.14	(1.01, 1.28)	
Degree	1.10	(0.96, 1.26)		1.11	(0.99, 1.24)	
Higher degree	0.90	(0.77, 1.05)	0.008	0.98	(0.87, 1.11)	0.006
Deprivation (IMD)[1]	1.05	(1.00, 1.10)	0.038	1.00	(0.95, 1.05)	0.938
Baseline SV [2]	1.08	(1.05, 1.10)	<0.0005	1.05	(1.03, 1.06)	<0.0005
>1 TV	1.16	(1.06, 1.27)	0.001	1.06	(0.96, 1.18)	0.249
Any computers	1.03	(0.86, 1.24)	0.721	1.07	(0.92, 1.26)	0.386
Any tablets	0.75	(0.64, 0.88)	<0.0005	0.80	(0.68, 0.95)	0.009
Any games consoles	1.26	(1.11, 1.44)	<0.0005	1.27	(1.08, 1.49)	0.003
Child gender	0.98	(0.91, 1.06)	0.517	0.89	(0.83, 0.96)	0.002
z-BMI score	1.00	(0.95, 1.05)	0.966	1.00	(0.96, 1.03)	0.904
parent SV (mins)[2]	1.03	(1.02, 1.04)	<0.0005	1.04	(1.03, 1.05)	<0.0005
Parental limits on SV	0.82	(0.76, 0.89)	<0.0005	0.93	(0.86, 1.01)	0.084

[1] increase per 1 standard deviation (14.0) in IMD: higher values indicate more deprived areas

[2] increase per 30 minutes screen-viewing at age 6

[3] increase per 30 minutes screen-viewing

For peer review only

Table S6– Associations between socioeconomic position and multi-screen-viewing at age 9

	WEEKDAY			WEEKEND		
	Ratio	95% CI	p-value	Ratio	95% CI	p-value
**Unadjusted Model**						
Household education						
Up to GCSE	1	(Reference)		1	(Reference)	
A level	0.82	(0.49, 1.37)		0.87	(0.53, 1.42)	
Degree	0.66	(0.38, 1.15)		0.63	(0.37, 1.06)	
Higher degree	0.52	(0.26, 1.05)	0.226	0.55	(0.31, 0.97)	0.083
Deprivation (IMD)[1]	1.42	(1.21, 1.67)	<0.0005	1.33	(1.16, 1.52)	<0.0005
**Adjusted Model[2]**						
Household education						
Up to GCSE	1	(Reference)		1	(Reference)	
A level	0.92	(0.51, 1.65)		0.90	(0.50, 1.59)	
Degree	0.80	(0.45, 1.42)		0.72	(0.38, 1.34)	
Higher degree	0.76	(0.34, 1.71)	0.808	0.64	(0.33, 1.25)	0.432
Deprivation (IMD)[1]	1.26	(1.12, 1.41)	<0.0005	1.27	(1.13, 1.43)	<0.0005
>1 TV	1.76	(1.12, 2.75)	0.014	1.38	(0.88, 2.16)	0.158
Any computers	0.70	(0.41, 1.20)	0.190	0.97	(0.54, 1.75)	0.920
Any tablets	1.90	(1.12, 3.22)	0.017	1.34	(0.46, 3.88)	0.590
Any games consoles	1.02	(0.59, 1.78)	0.943	1.65	(0.95, 2.85)	0.076
Child gender	1.01	(0.73, 1.40)	0.945	1.06	(0.76, 1.49)	0.730
z-BMI score	1.08	(0.92, 1.25)	0.348	0.93	(0.82, 1.05)	0.246
parent multi-SV (mins)[2]	1.26	(1.17, 1.35)	<0.0005	1.21	(1.13, 1.29)	<0.0005
Parental limits on SV	0.62	(0.50, 0.78)	<0.0005	0.72	(0.53, 0.97)	0.033

[1] increase per 1 standard deviation (14.0) in IMD: higher values indicate more deprived areas

[2] Adjusted for child gender, child BMI, presence of TVs, computers, tablets and games consoles in the household, parental screen-viewing and parental limiting of screen-viewing

[3] increase per 30 minutes multi-screen-viewing

For peer review only

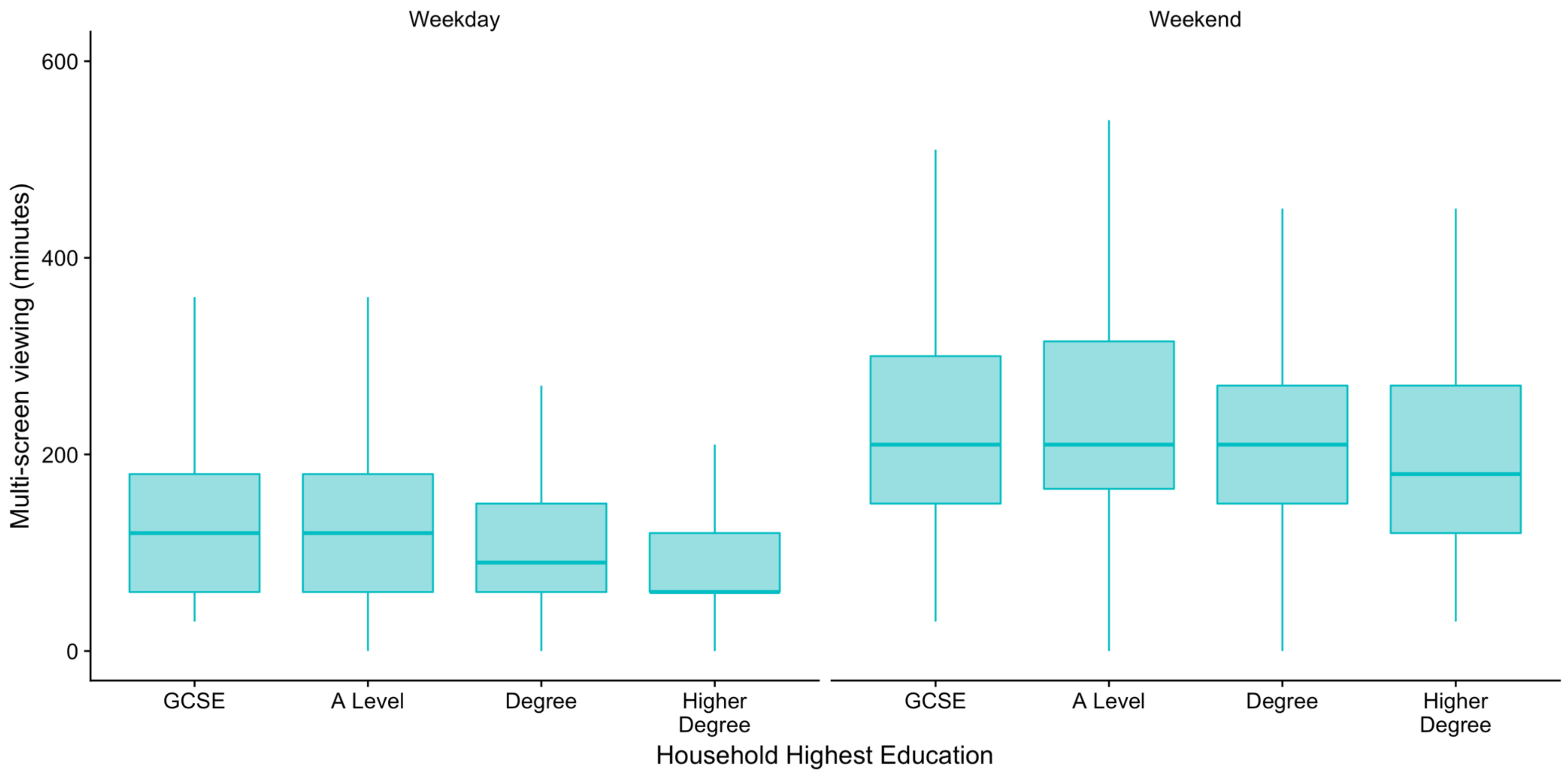

Figure S1: Number of minutes spent multi-screen viewing by household education at age 9, for weekdays (left) and weekends (right).

STROBE 2007 (v4) Statement—Checklist of items that should be included in reports of *cohort studies*

Section/Topic	Item #	Recommendation	Reported on page #
**Title and abstract**	1	(*a*) Indicate the study's design with a commonly used term in the title or the abstract	Title page
		(*b*) Provide in the abstract an informative and balanced summary of what was done and what was found	2-3
**Introduction**			
Background/rationale	2	Explain the scientific background and rationale for the investigation being reported	5-6
Objectives	3	State specific objectives, including any prespecified hypotheses	6
**Methods**			
Study design	4	Present key elements of study design early in the paper	6-7
Setting	5	Describe the setting, locations, and relevant dates, including periods of recruitment, exposure, follow-up, and data collection	6-7
Participants	6	(*a*) Give the eligibility criteria, and the sources and methods of selection of participants. Describe methods of follow-up	6-7
		(*b*) For matched studies, give matching criteria and number of exposed and unexposed	n/a
Variables	7	Clearly define all outcomes, exposures, predictors, potential confounders, and effect modifiers. Give diagnostic criteria, if applicable	7-8
Data sources/ measurement	8*	For each variable of interest, give sources of data and details of methods of assessment (measurement). Describe comparability of assessment methods if there is more than one group	7-8
Bias	9	Describe any efforts to address potential sources of bias	8-9
Study size	10	Explain how the study size was arrived at	6-7
Quantitative variables	11	Explain how quantitative variables were handled in the analyses. If applicable, describe which groupings were chosen and why	8-9
Statistical methods	12	(*a*) Describe all statistical methods, including those used to control for confounding	8-9
		(*b*) Describe any methods used to examine subgroups and interactions	8-9
		(*c*) Explain how missing data were addressed	9
		(*d*) If applicable, explain how loss to follow-up was addressed	n/a
		(*e*) Describe any sensitivity analyses	n/a
**Results**			

For peer review only

Participants	13*	(a) Report numbers of individuals at each stage of study—eg numbers potentially eligible, examined for eligibility, confirmed eligible, included in the study, completing follow-up, and analysed	7
		(b) Give reasons for non-participation at each stage	n/a
		(c) Consider use of a flow diagram	
Descriptive data	14*	(a) Give characteristics of study participants (eg demographic, clinical, social) and information on exposures and potential confounders	9-11
		(b) Indicate number of participants with missing data for each variable of interest	9
		(c) Summarise follow-up time (eg, average and total amount)	n/a
Outcome data	15*	Report numbers of outcome events or summary measures over time	9-10
Main results	16	(*a*) Give unadjusted estimates and, if applicable, confounder-adjusted estimates and their precision (eg, 95% confidence interval). Make clear which confounders were adjusted for and why they were included	11-12
		(*b*) Report category boundaries when continuous variables were categorized	n/a
		(*c*) If relevant, consider translating estimates of relative risk into absolute risk for a meaningful time period	n/a
Other analyses	17	Report other analyses done—eg analyses of subgroups and interactions, and sensitivity analyses	
**Discussion**			
Key results	18	Summarise key results with reference to study objectives	12-13
**Limitations**			
Interpretation	20	Give a cautious overall interpretation of results considering objectives, limitations, multiplicity of analyses, results from similar studies, and other relevant evidence	13-14
Generalisability	21	Discuss the generalisability (external validity) of the study results	14-15
**Other information**			
Funding	22	Give the source of funding and the role of the funders for the present study and, if applicable, for the original study on which the present article is based	16

*Give information separately for cases and controls in case-control studies and, if applicable, for exposed and unexposed groups in cohort and cross-sectional studies.

Note: An Explanation and Elaboration article discusses each checklist item and gives methodological background and published examples of transparent reporting. The STROBE checklist is best used in conjunction with this article (freely available on the Web sites of PLoS Medicine at http://www.plosmedicine.org/, Annals of Internal Medicine at http://www.annals.org/, and Epidemiology at http://www.epidem.com/). Information on the STROBE Initiative is available at www.strobe-statement.org.

For peer review only

BMJ Open

Associations between socio-economic position and changes in children's screen-viewing between ages 6 and 9: a longitudinal study

Journal:	*BMJ Open*
Manuscript ID	bmjopen-2018-027481.R3
Article Type:	Original research
Date Submitted by the Author:	05-Nov-2019
Complete List of Authors:	Salway, Ruth; University of Bristol, Centre for Exercise, Nutrition and Health Sciences<br>Emm-Collison, Lydia; University of Bristol, Centre for Exercise, Nutrition and Health Sciences<br>Sebire, Simon; University of Bristol, Centre for Exercise, Nutrition and Health Sciences<br>Thompson, Janice; The University of Birmingham, School of Sport & Exercise Sciences<br>Jago, Russ; University of Bristol, Centre for Exercise, Nutrition and Health Sciences
**Primary Subject Heading**:	Public health
Secondary Subject Heading:	Paediatrics
Keywords:	screen-viewing, PAEDIATRICS, TV, computer games, cohort

SCHOLARONE™
Manuscripts

Associations between socio-economic position and changes in children's screen-viewing between ages 6 and 9: a longitudinal study

Ruth Salway[1,*], Lydia Emm-Collison[1], Simon J. Sebire[1], Janice L. Thompson[2] and Russ Jago[1]

[1] Centre for Exercise, Nutrition & Health Sciences, School for Policy Studies, University of Bristol, 8 Priory Road, Bristol, BS8 1TZ, UK.

[2] School of Sport, Exercise and Rehabilitation Sciences, University of Birmingham, Birmingham, B15 2TT, UK

[*] **Corresponding author:** Dr Ruth Salway, Centre for Exercise, Nutrition & Health Sciences, School for Policy Studies, University of Bristol, 8 Priory Road, Bristol, BS8 1TZ, UK.

Email: ruth.salway@bristol.ac.uk; Telephone: +44 (0)117 3310463

Word count: **Manuscript** = 3475 words **Abstract** = 285 words (max 300)

For peer review only - http://bmjopen.bmj.com/site/about/guidelines.xhtml

ABSTRACT

Objectives: To explore socio-economic differences in screen-viewing at age 6 and 9, and how these are related to different media use.

Design: Longitudinal cohort study.

Setting: Children recruited from 57 state-funded primary schools in Southwest England, UK.

Participants: 1299 children at age 5-6, 1223 children at age 8-9, including 685 children at both timepoints.

Outcome measures: Children's total screen-viewing time (parent-reported) and time spent using multiple screen devices simultaneously (multiscreen viewing), for weekdays and weekends.

Methods: Negative binomial regression was used to model associations between socio-economic variables (highest household education and area deprivation) and total screen-viewing at age 6 and the change from age 6 to 9. We additionally adjusted for child characteristics, parental influences and media devices in the home. Multiscreen viewing was analysed separately.

Results: Household education was associated with children's screen-viewing at age 6 with lower screen-viewing in higher socio-economic groups (21-27% less in households with a Degree or Higher Degree, compared to GCSE). These differences were explained by the presence of games consoles, parental limits on screen-viewing, and average parent screen-viewing. Between ages 6 and 9, there were larger increases in screen-viewing for children from A level and Degree households (13% and 6% respectively in the week) and a decrease in Higher Degree households (16%), compared to GCSE households. Differences by household education remained when adjusting for media devices and parental factors.

Conclusions: Children's screen-viewing patterns differ by parental education with higher levels of viewing among children living in households with lower educational qualifications. These

For peer review only - http://bmjopen.bmj.com/site/about/guidelines.xhtml

differences are already present at age 6, and continue at age 9. Strategies to manage child sedentary time, and particularly screen-viewing, may need to take account of the socio-economic differences and target strategies to specific groups.

Keywords: screen viewing, TV, computer games, cohort, children

For peer review only

ARTICLE SUMMARY

Strengths and limitations of this study

Strengths

- Longitudinal study screen viewing on both weekdays and weekends at two time points.
- Time frame covers an important period of change in child viewing behaviours.
- Data collection period coincided with a period of rapid change in screen-viewing technology which has facilitated the presentation of contemporary data.

Limitations

- We did not explicitly ask parents about tablet or smartphone use and so have not been able to capture this directly.
- A change in the way education was recorded may result in misclassification in a small number of situations where household composition has changed or where people have obtained additional qualifications.

For peer review only

INTRODUCTION

Sedentary behaviour has been associated with increased risk of obesity and elevated levels of cardio-metabolic risk factors among children and adolescents[1, 2]. Screen-viewing is a very common form of sedentary behaviour among children and young people[3], and higher screen-viewing has been associated with higher body mass index, poorer psychological well-being and poorer academic achievement among children[4-6]. The American Academy of Pediatrics recommends restricting children's screen-viewing, although it does not specify a limit[7]. An international study estimated that 11 year old boys engaged in an average of 5.3 h/day of screen-viewing on weekdays in 2010 (4.4 h/day for girls) with higher estimates at weekends[8]. The paper therefore shows that by the end of primary school children are engaging in high levels of screen viewing. Sedentary behaviour tracks at moderate levels from childhood into adolescence[9], with TV viewing tracking from childhood to adulthood[10]. Moreover, screen-viewing has increased in children by around 1.3h/day on weekdays (2.0 h/day on weekends) between 2002 and 2010 [8]. Thus, screen-viewing is an increasingly common behaviour among children and with levels increasing as children age. We therefore need to understand patterns of behaviour develop during childhood, and particularly at the start of primary school when patterns of behaviour are established. As such, information on levels of screen-viewing and how they change during early school years is required.

Much of the current literature on screen-viewing focuses on TV viewing, but screen-viewing has evolved with technology to include TV viewing, computer games, tablets, mobile phones and multiscreen viewing (in which children use two or more devices at the same time)[11] , and become more diverse, for example encompassing on-demand TV and online gaming[3]. Thus, to try and change behaviour there is a need to understand what devices children are using and the types of viewing in which children engage on different days of the week.

For peer review only

For peer review only - http://bmjopen.bmj.com/site/about/guidelines.xhtml

Socio-economic position has been associated with poorer health outcomes among children and young people. It is important to note, however that different indicators of socio-economic position, such as education, income and deprivation, measure different, often related aspects, although they are strongly correlated[12, 13]. For example, in England at the end of primary school (age 11) in 2018 the prevalence of child obesity in the most deprived areas is more than double that of those in the least deprived neighbourhoods (27% vs. 12%)[14], and TV viewing has been identified as a mediator for this association[15]. A number of studies have found an inverse relationship between SES and sedentary time[16], sedentary behaviours[17, 18] and screen-viewing specifically[19, 20] with children's daily screen time varying between 1.7 and 2.4 hours/day for high and low SES families respectively[19]. Sedentary behaviour increases as children age[18], but as much of the evidence is cross-sectional it is not clear whether different SES groups change at the same rate. Longitudinal evidence between ages 2 and 9 suggests that while TV viewing increases similarly for different educational subgroups, there may be different trajectories for different household income groups[21]. It is also not clear whether these SES differences are due to differences in other factors or not. Child obesity, ownership of different media devices, parental screen-viewing and parental limits on screen-viewing have all been found to affect children's screen-viewing and differ between SES groups [1, 19, 22-29]. There is thus a need to better understand SES differences, and especially how these change over time.

The aim of this paper was to explore socio-economic differences in screen-viewing at age 6, and the change in screen-viewing between ages 6 and 9, and whether any differences can be explained by other factors such as different media devices and parental influences. Developing knowledge in this area will support the creation of targeted behaviour change programmes to reduce screen-viewing.

METHODS

For peer review only - http://bmjopen.bmj.com/site/about/guidelines.xhtml

B-PROACT1V is a longitudinal study that aimed to examine the physical activity and sedentary behaviours of primary school children aged 5-11 years, and their parents (described in detail elsewhere [30-32]). The study received ethical approval from the School of Policy Studies Ethics Committee at the University of Bristol, UK, and written parental consent was received for all participants [33] (Reference B-Proact1V study, approval 11th November 2011.) In Phase 1, all children in Year 1 of primary school (aged 5-6 years) from 57 schools in and around Bristol were invited to participate, with data collected between January 2012 and July 2013. In Phase 2, when the children were in Year 4 (aged 8-9 years), all schools from Phase 1 were invited to participate, with 47 schools agreeing. All children were eligible regardless of whether they had participated in Phase 1, and data collection took place between March 2015 and July 2016. Data were collected for 1299 children in Year 1 and 1223 children in Year 4, with 685 children included in both phases. Self-identified 'first' parents completed a questionnaire about personal and family characteristics while 'second' parents completed a shorter questionnaire. This paper uses data from the first parent questionnaires: 1085 (84%) from Year 1, 997 (82%) from Year 4 and 509 (74%) at both time points.

Screen-viewing data

In both years, the first parent was asked about the number of hours their child typically spent engaging in specific screen-viewing behaviours on weekdays and at weekends: TV, computer, games consoles. Additionally, both parents were asked about their own screen-viewing. These responses were recorded as either 'None' or in hourly categories from '0-1 hours' up to '4 hours or more', and recoded based on midpoints to give the average number of minutes spent in each type of screen-viewing on weekdays and weekends. These were summed to form the average total number of minutes spent on any type of screen-viewing. Where two parents completed questionnaires, parental screen-viewing was taken as the average (approximately 27% of respondents in each year). At the second phase, parents were also asked about the time they and their child spent multiscreen-viewing (i.e. using two or more devices at once), with responses as above recoded to midpoints [11].

For peer review only - http://bmjopen.bmj.com/site/about/guidelines.xhtml

Socio-economic data

At age 6, the first parent was asked their highest educational qualification, while at age 9, they were asked the highest education qualification of anyone in the household. We combined these to form the highest household educational qualification recorded at either time with categories ‘Up to GCSE (General Certificate of Secondary Education) or equivalent’ (qualification at age 16), ‘A level/National Vocational Qualification or equivalent’ (qualification at age 18), ‘University Degree or equivalent’ and ‘Higher Degree (MSc/PhD) or equivalent’. In addition, Index of Multiple Deprivation (IMD) scores, based on the English Indices of Deprivation (http://data.gov.uk/dataset/index-of-multiple-deprivation), were assigned to each child based on their reported home postcode, with higher IMD scores indicating a greater level of deprivation. Household education captures individual long-term socio-economic position, such as knowledge, and also provides an indirect indication of income, while IMD captures the socio-economic conditions of the area in which they live.

For peer review only

Other measurements

Child gender was reported by the first parent. Child height and weight were recorded to the nearest 0.1 cm and 0.1 kg, respectively, by trained fieldworkers at each time point. Body mass index (BMI) was calculated and converted to an age- and sex-specific standard deviation score based on UK reference curves[34, 35]. The first parent was asked whether they limited the time their child spent engaging in three different types of screen-viewing (TV, computer and video games) with responses from 1 ‘Strongly disagree’ to 4 ‘Strongly agree’. The average of these was used to capture parental limits on screen-viewing. We also asked about the number of media devices in the home: TVs, computers (desktop or laptop), tablet computers and games consoles (including handheld consoles).

Patient and Public Involvement

The research questions for this study emerged from research that was conducted with 1078 Year 1 children. A sub-set of 53 parents of the children who took part in the Year 1 study participated in interviews to help guide future research. Participants provided verbal assent (with parental consent) to join the study, and summaries of project findings are sent to all participants via study schools.

Statistical Analysis

Descriptive summaries of children's screen-viewing on weekdays and weekends and other participant characteristics were produced by household education group for age 6 and age 9. To aid interpretation, the screen-viewing variables are reported in summaries as continuous variables. However, the underlying variables are discrete and so in the main analyses we used negative binomial regression models (similar to Poisson models but suitable for over-dispersed count data) to model weekday and weekend screen-viewing separately. We considered four main models. Model 1 explored cross-sectional associations between socioeconomic position (household education and IMD) and total minutes of screen-viewing at age 6. Model 2 additionally adjusted for possible mediators and confounders: child gender, child BMI, number of devices in the home (more than one TV, and presence of any computers, tablets or games consoles), parental screen-viewing and parental behaviour on limiting screen-viewing. Models 3 (unadjusted) and 4 (adjusted for covariates) explored the longitudinal change between ages 6 and 9; that is, associations between socioeconomic position and total minutes of screen-viewing at age 9, adjusting for baseline screen-viewing at age 6. Finally, we also explored the association between socioeconomic position and multiscreen viewing as a separate outcome. Robust standard errors were used to account for clustering of children within schools, and all analyses are based on complete cases. Model results are presented as screen time ratios (rate ratios), defined as the exponent of the model coefficients. An increase of one unit in a given predictor variable is associated with a multiplicative increase in screen-viewing, holding the

For peer review only

For peer review only - http://bmjopen.bmj.com/site/about/guidelines.xhtml

other predictor variables in the model constant. Model assumptions were checked via model diagnostics, and all analysis was done in Stata v15.

RESULTS

Children's screen-viewing differed between households with differing levels of education at both ages 6 and 9, with higher screen-viewing in lower qualified households, decreasing as education increased, with 35-50 minutes difference between the highest and lowest household education qualifications (Figure 1). Time spent screen-viewing was higher at age 9 than age 6, and higher at weekends than during the week among all education groups, with time spent watching TV and playing games consoles nearly doubling at weekends (Table S1 and S2). Time spent engaged in multiscreen viewing was also higher for households with lower levels of qualification, with children in households where GCSEs were the highest qualification spending over twice as much time in multiscreen viewing as those where the highest qualification was a postgraduate degree, on both weekdays and weekends. Missing data ranged between 0-3% (Table S3).

There were differences in the number and types of media devices in the home by household education (Figure 2). Households where GCSE and A Levels were the highest qualification had more TVs and games consoles in the house, while Higher Degree households had more computers. There were small increases in most devices between age 6 and age 9. The average number of tablet devices was the same across all levels of household education but increased fourfold to an average of 2.2 per household between the two assessment periods. Parental screen-viewing differed with household education by similar amounts to their children, again with higher screen-viewing among those with lower level qualifications. Parental screen-viewing was also higher at the second time point, especially among the Higher Degree educated households, and differences with education were smaller.

For peer review only

For peer review only - http://bmjopen.bmj.com/site/about/guidelines.xhtml

Table 1 shows the associations between socioeconomic position and total screen-viewing at age 6, in terms of ratios for screen time. For example, a factor of 0.79 for Degree-level household education (Table 1, weekday) means that screen-viewing time decreased by 21% for children in households where a University degree or equivalent was the highest qualification, compared to children in households where the highest qualifications were GCSEs. In the unadjusted model (Model 1) there were differences in screen-viewing by household education for both weekdays and weekends, with lower screen-viewing for children in households with higher education. The GCSE and A level education groups were similar, but children in households with a Degree or Higher degree engaged in 21-27% less screen-viewing. There was no association with IMD. The adjusted model (Model 2) adjusts for possible mediators: number of devices, child BMI, parental screen-viewing and parental limits on screen-viewing all measured at age 6. These factors accounted for household education differences on both weekdays and weekends. Children's screen-viewing was higher in households with games consoles, and lower when parents were more likely to limit screen-viewing (Table S4). There were weak associations with parental screen-viewing and child BMI.

Models 3 (unadjusted) and 4 (adjusted) examined the change in screen-viewing between ages 6 and 9 (Table 2). In Model 3, adjusting for baseline screen-viewing, there were differences in screen-viewing by household education, with larger increases in screen-viewing for children from A level and Degree households (13% and 6% respectively) and 16% less increase in Higher Degree households, compared to GCSE households in the week. Similar patterns were seen at weekends, with the A level and Degree groups increasing more than the GCSE and Higher Degree groups. There was no association with IMD. In Model 4, when adjusting for media devices in the home, child BMI and parental influences at age 9, household education differences still remain, with larger increases for the A level and Degree groups. An increase in screen-viewing was strongly associated with the presence of games consoles and multiple TVs (weekdays only), with weaker associations

For peer review only

For peer review only - http://bmjopen.bmj.com/site/about/guidelines.xhtml

with parental screen-viewing (Table S5). Presence of tablets was associated with a decrease in screen-viewing, as was parental limits on screen-viewing in the week, but not at weekends.

Multiscreen viewing was associated with both IMD but not with household education, with children's multiscreen viewing higher in families living in more deprived areas (Table S6 and Figure S1). When adjusting for other factors, the association with deprivation remained, with multiscreen viewing 26-27% higher for every standard deviation increase in IMD score. Higher amounts of multiscreen viewing were associated with higher levels of parental multiscreen viewing and the presence of tablet devices and multiple TVs in the week, and lower levels of multiscreen viewing were associated with increased parental limits on screen-viewing.

DISCUSSION

The data presented in this paper have shown an association between children's screen-viewing and highest household education level, with higher screen-viewing in lower socio-economic groups. These differences were already present at age 6 and continued to be evident at age 9. Socio-economic differences at age 6 were accounted for by other factors, especially parental factors such as their own screen-viewing and their behaviour in limiting their children's screen-viewing. However, while the time children spent engaged in screen-viewing increased in all households between ages 6 and 9, regardless of education, they increased by different amounts depending on household education. Adjusting for baseline screen-viewing at age 6, screen-viewing increased most over the time period for children in households where the highest qualifications were A Levels or a university degree compared to GCSE, with the smallest increase among Higher Degree households. In A-level educated households, this reflected a "catching-up" with the level of screen-viewing seen in GCSE-educated households. These findings imply socio-economic differences in both amount of screen-viewing, and the way in which screen-viewing patterns develop. These differences were related to

For peer review only - http://bmjopen.bmj.com/site/about/guidelines.xhtml

household education rather than area-level deprivation, suggesting that it is long-term socio-economic aspects such as parental knowledge, ability to engage and communicate with services, and possibly income that are important, rather than the socio-economic conditions of the area, such as availability of local resources. In contrast, multiscreen viewing is associated with area deprivation rather than education and so could represent a proxy for short-term current income or possibly a neighbourhood effect of what is considered to be 'typical' screen-viewing behaviour. As such, strategies to manage child sedentary time, and particularly different types of screen-viewing, may need to take account of the socio-economic differences and target strategies to specific groups.

Ownership of media devices such as TVs, phones and games consoles, have previously been found to be associated with higher levels of screen-viewing, and also inversely with socio-economic status [24, 28]. We found that the number of media devices in the home differed by household education, with more TVs and games consoles in households with GCSE and A level qualifications, and more computers in households with a university or higher degree. While the number of devices changed overall between the two assessment points, most notably with a large increase in tablet devices and a slight decrease in the number of computers, socio-economic differences were small and did not account for the observed differences in total screen-viewing between household education. We found that higher screen-viewing at age 6 was associated with games consoles at the weekends only, but not with multiple TVs or computers, and larger increases in screen-viewing between ages 6 and 9 were associated with games consoles and multiple TVs in the week. However, we also saw tablet ownership associated with smaller increases in screen-viewing between ages 6 and 9. This suggests that the relationship between different devices and screen-viewing is complex, and may be changing over time. Thus, we extend previous findings to show that while access to devices may contribute to baseline screen-viewing, it does not entirely explain socio-economic differences in the increase in screen-viewing between ages 6 and 9.

For peer review only

For peer review only - http://bmjopen.bmj.com/site/about/guidelines.xhtml

There were weak associations between parent and child screen-viewing, with every 30 minutes of parental screen-viewing associated with a 2% increase in child screen-viewing. Parental limits on screen-time were strongly associated with lower screen-viewing at age 6 and less time spent multi-screen-viewing at age 9, but associations were stronger for weekdays than weekends and limiting screen time was associated with changes in screen-viewing between ages 6 and 9 on weekdays only. Interestingly, analysis of interviews conducted with a sub-sample of the parents in the study showed that many parents are uneasy about managing non-TV screen-time and feel that they struggle to keep up with rapid technological change[36]. Collectively, these findings may suggest that there is a need to help parents to identify effective ways to manage constantly adapting forms of viewing.

Tablet ownership did not vary with household education but increased greatly overall between ages 6 and 9. A limitation of this project is that we did not assess tablet or smartphone use as a specific behaviour in the questionnaire. This may under-estimate total screen-viewing, especially for those who are heavy users of these devices, and may explain the observed negative association between screen-viewing and tablet ownership. We also note that tablet ownership is strongly associated with higher levels of multiscreen-viewing on weekdays. The widespread ownership of tablets is particularly important for understanding the complexity of screen-viewing, as watching TV, playing games and using the internet can all be done via a tablet and, as such, identifying the behaviour engaged in while using a tablet is challenging. Thus, it may be that in the future there is a specific need to assess tablet time, and to differentiate the various ways in which tablets are used. For example, applications that allow parents and children to agree targets to limit the time spent on games and other non-educational activities, could also be used as a feedback and monitoring device.

For peer review only - http://bmjopen.bmj.com/site/about/guidelines.xhtml

The data presented in this paper add to the previous evidence base which has shown that socio-economic position is associated with sedentary time and screen-viewing. Previous work within this area has focussed on cross-sectional associations between TV time and various indicators of socio-economic position [15-20]. Collectively this body of evidence identifies an inverse association with children residing in more socioeconomically disadvantaged households engaging in more TV viewing. The findings from this paper greatly extend this work by showing that these patterns are existent cross-sectionally and prospectively. The prospective associations, are however, particularly informative as they show that these differences are evident at age 6 and continue as child ages. These findings suggest that we need a range of options to help families to manage their screen-time and that these made need to be tailored to specific socio-economic groups. For example, for families from lower socio-economic groups there is likely to be a need for programs that focus on stopping the development of screen-viewing behaviours in early childhood. This could be parental education programs that are combined with behaviour change techniques, while for older children from all socio-economic groups there may need to be more of a focus on family-focussed management information where children and parents work together to set family viewing goals [37]. It is unlikely that a single strategy will work on its own[38], and as such there is need for multiple strategies across childhood that adapt to the age and viewing habits of the child and the family.

The major strengths of this study are the information on a variety of different types of screen-viewing at two time points which span an important period of change in child viewing behaviours. The data collection period (2012-16) coincided with a period of rapid change in screen-viewing technology which has facilitated the presentation of contemporary data. There are, however, several limitations that need to be considered. Firstly, we did not explicitly ask parents about tablet or smartphone use. As data collection covers a time when screen use is changing rapidly, (for example, global tablet sales increased from 116 million in 2012[39] to 207 million in 2015[40]) this is an

For peer review only - http://bmjopen.bmj.com/site/about/guidelines.xhtml

important aspect we have not been able to capture directly. Secondly, the differences in the education variable between Year 1 (first parent's education) and Year 4 (household education) may result in inaccuracies in situations where household composition has changed or where people have obtained additional qualifications between the two time points, although we believe that the number of these cases is likely to be small. Children's screen-viewing was parent-reported, and amounts of multiscreen-viewing, in particular, may not be well-captured, although general patterns might be more robust. This is a longitudinal study, and so some children were lost to follow-up, although the majority were through non-participating schools. In addition, as we have included socioeconomic position variables directly, our models will not biased due to differential follow-up rates by socioeconomic status. Finally, as parental screen-viewing increased between 2012 and 2015 this may indicate a general change in viewing patterns, and the increases reported between the two age periods could reflect secular changes as opposed to age-related differences. As such, it would be important to identify if comparable patterns are evident in other datasets.

Conclusion

Children's screen-viewing patterns differ by parental education with higher levels of viewing among children living in households with lower levels of education. These differences are already present at age 6, and continue at age 9, even when accounting for differences in baseline screen-viewing and device ownership. Socio-economic differences narrow with age as children in households with higher qualifications gain greater access to screen-viewing devices. Strategies to manage child sedentary time, and particularly screen-viewing, may need to take account of the socio-economic differences and target strategies to specific groups. For example, specific early intervention strategies to reduce screen-viewing in children from lower socio-economic groups are likely to help stop high levels of screen-viewing from developing and reduce inequalities.

For peer review only

For peer review only - http://bmjopen.bmj.com/site/about/guidelines.xhtml

ACKNOWLEDGEMENTS

We would like to thank all of the families and schools that have taken part in the B-PROACT1V project. We would also like to thank all current and previous members of the research team who are not authors on this paper.

COMPETING INTERESTS

We have no competing interest to report.

FUNDING

This work was supported by the British Heart Foundation (ref PG/11/51/28986 and SP 14/4/31123). The funder had no involvement in data analysis, data interpretation or writing of the paper.

AUTHOR CONTRIBUTIONS

RJ, SJS and JLT were involved in the design of this study and in seeking funding for it. The paper was conceived by RJ and RS, and RS performed all analyses. RS and RJ wrote the first draft of the paper and RJ coordinated contributions from other authors. All authors (RJ, SJS, JLT, LEC) made critical comments on drafts of the paper.

DATA SHARING STATEMENT

The datasets generated during the current study are not publicly available due as the project is ongoing and data are not ready for archiving. We will consider reasonable requests for access to the data once the project is complete in 2019.

REFERENCES

1. Ekelund U, Brage S, Froberg K, Harro M, Anderssen S, Sardinha LB, Riddoch CJ, Andersen LB: **TV Viewing and Physical Activity Are Independently Associated with Metabolic Risk in Children: The European Youth Heart Study.** *PLoS Med* 2006, **3:**e488.

For peer review only - http://bmjopen.bmj.com/site/about/guidelines.xhtml

2. Ekelund U, Luan J, Sherar LB, Esliger DW, Griew P, Cooper A, International Children's Accelerometry Database C: **Moderate to vigorous physical activity and sedentary time and cardiometabolic risk factors in children and adolescents.** *JAMA* 2012, **307:**704-712.
3. Saunders TJ, Vallance JK: **Screen Time and Health Indicators Among Children and Youth: Current Evidence, Limitations and Future Directions.** *Appl Health Econ Health Policy* 2017, **15:**323-331.
4. Corder K, Atkin AJ, Bamber DJ, Brage S, Dunn VJ, Ekelund U, Owens M, van Sluijs EM, Goodyer IM: **Revising on the run or studying on the sofa: prospective associations between physical activity, sedentary behaviour, and exam results in British adolescents.** *Int J Behav Nutr Phys Act* 2015, **12:**106.
5. Page AS, Cooper AR, Griew P, Jago R: **Children's Screen Viewing is Related to Psychological Difficulties Irrespective of Physical Activity.** *Pediatrics* 2010, **126:**e1011-1017.
6. Jago R, Baranowski T, Baranowski JC, Thompson D, Greaves KA: **BMI from 3-6 y of age is predicted by TV viewing and physical activity, not diet.** *Int J Obes (Lond)* 2005, **29:**557-564.
7. American Academy of Pediatrics: **Media Use in School-Aged Children and Adolescents.** *Pediatrics* 2016, **138:**e20162591.
8. Bucksch J, Sigmundova D, Hamrik Z, Troped PJ, Melkevik O, Ahluwalia N, Borraccino A, Tynjala J, Kalman M, Inchley J: **International Trends in Adolescent Screen-Time Behaviors From 2002 to 2010.** *J Adolesc Health* 2016, **58:**417-425.
9. Biddle SJ, Pearson N, Ross GM, Braithwaite R: **Tracking of sedentary behaviours of young people: a systematic review.** *Prev Med* 2010, **51:**345-351.
10. Smith L, Gardner B, M. H: **Childhood correlates of adult TV viewing time: a 32-year follow-up of the 1970 British Cohort Study.** *J Epidemiol Community Health* 2015, **69:**309-313.
11. Jago R, Sebire SJ, Gorely T, Cillero IH, Biddle SJ: **"I'm on it 24/7 at the moment": a qualitative examination of multi-screen viewing behaviours among UK 10-11 year olds.** *Int J Behav Nutr Phys Act* 2011, **8:**85.
12. Galobardes B, Shaw M, Lawlor DA, Lynch JW, Davey Smith G: **Indicators of socioeconomic position (part 1).** *J Epidemiol Community Health* 2006, **60:**7-12.
13. Galobardes B, Shaw M, Lawlor DA, Lynch JW, Davey Smith G: **Indicators of socioeconomic position (part 2).** *J Epidemiol Community Health* 2006, **60:**95-101.
14. NHS Digital: **National Child Measurement Programme, England 2017-2018 school year.** UK; 2018.
15. Morgenstern M, Sargent JD, Hanewinkel R: **Relation between socioeconomic status and body mass index: evidence of an indirect path via television use.** *Arch Pediatr Adolesc Med* 2009, **163:**731-738.
16. Sherar LB, Griffin TP, Ekelund U, Cooper AR, Esliger DW, van Sluijs EM, Bo Andersen L, Cardon G, Davey R, Froberg K, et al: **Association between maternal education and objectively measured physical activity and sedentary time in adolescents.** *J Epidemiol Community Health* 2016, **70:**541-548.
17. Coombs N, Shelton N, Rowlands A, Stamatakis E: **Children's and adolescents' sedentary behaviour in relation to socioeconomic position.** *J Epidemiol Community Health* 2013, **67:**868-874.
18. Moraeus L, Lissner L, Olsson L, Sjoberg A: **Age and time effects on children's lifestyle and overweight in Sweden.** *BMC Public Health* 2015, **15:**355.
19. Tandon P, Chuan Zhou C, Sallis J, Cain K, Frank L, Saelens B: **Home environment relationships with children's physical activity, sedentary time, and screen time by socioeconomic status.** *Int J Behav Nutr Phys Act* 2012, **9:**88.

For peer review only

For peer review only - http://bmjopen.bmj.com/site/about/guidelines.xhtml

20. Drenowatz C, Eisenmann JC, Pfeiffer KA, Welk G, Heelan K, Gentile D, Walsh D: **Influence of socio-economic status on habitual physical activity and sedentary behavior in 8- to 11-year old children.** *BMC Public Health* 2010, **10:**214.
21. Yang-Huang J, van Grieken A, Moll HA, Jaddoe VWV, Wijtzes AI, Raat H: **Socioeconomic differences in children's television viewing trajectory: A population-based prospective cohort study.** *PLoS One* 2017, **12:**e0188363.
22. Jago R, Page A, Froberg K, Sardinha L, Klasson-Heggebo L, Andersen L: **Screen-viewing and the home TV environment: The European Youth Heart Study.** *Prev Med* 2008, **47:**525 - 529.
23. Jago R, Davison K, Thompson JL, Page AS, Brockman R, Fox KR: **Parental sedentary restriction, maternal parenting style and TV viewing among 10-11 year olds** *Pediatrics* 2011, **128:**e572-e578.
24. Jago R, Stamatakis E, Gama A, Carvalhal IM, Nogueira H, Rosado V, Padez C: **Parent and child screen-viewing time and home media environment.** *Am J Prev Med* 2012, **43:**150-158.
25. Jago R, Sebire SJ, Edwards MJ, Thompson JL: **Parental TV viewing, parental self-efficacy, media equipment and TV viewing among preschool children.** *Eur J Pediatr* 2013, **172:**1543-1545.
26. Jago R, Thompson JL, Sebire SJ, Wood L, Pool L, Zahra J, Lawlor DA: **Cross-sectional associations between the screen-time of parents and young children: differences by parent and child gender and day of the week.** *International Journal of Behavioral Nutrition and Physical Activity* 2014, **11**.
27. Lauricella AR, Wartella E, Rideout VJ: **Young children's screen time: The complex role of parent and child factors.** *Journal of Applied Developmental Psychology* 2015, **36:**11-17.
28. Dumuid D, Olds TS, Lewis LK, Maher C: **Does home equipment contribute to socioeconomic gradients in Australian children's physical activity, sedentary time and screen time?** *BMC Public Health* 2016, **16:**736.
29. Gebremariam MK, Altenburg TM, Lakerveld J, Andersen LF, Stronks K, Chinapaw MJ, Lien N: **Associations between socioeconomic position and correlates of sedentary behaviour among youth: a systematic review.** *Obes Rev* 2015, **16:**988-1000.
30. Jago R, Sebire SJ, Wood L, Pool L, Zahra J, Thompson JL, Lawlor DA: **Associations between objectively assessed child and parental physical activity: a cross-sectional study of families with 5-6 year old children.** *BMC Public Health* 2014, **14:**655.
31. Jago R, Thompson JL, Sebire SJ, Wood L, Pool L, Zahra J, Lawlor DA: **Cross-sectional associations between the screen-time of parents and young children: differences by parent and child gender and day of the week.** *Int J Behav Nutr Phys Act* 2014, **11:**54.
32. Jago R, Solomon-Moore E, Macdonald-Wallis C, Sebire SJ, Thompson JL, Lawlor DA: **Change in children's physical activity and sedentary time between Year 1 and Year 4 of primary school in the B-PROACT1V cohort.** *Int J Behav Nutr Phys Act* 2017, **14:**33.
33. Jago R, Bailey R: **Ethics and paediatric exercise science: Issues and making a submission to a local ethics and research committee.** *Journal of Sport Sciences* 2001, **19:**527-535.
34. Cole TJ, Freeman JV, Preece MA: **Body mass index reference curves for the UK, 1990.** *Arch Dis Child* 1995, **73:**25-29.
35. Cole TJ, Bellizzi MC, Flegal KM, H. DW: **Establishing a standard definition for child overweight and obesity worldwide: international survey.** *BMJ Open* 2000, **320:**1240-1243.
36. Jago R, Solomon-Moore E, Toumpakari Z, Lawlor DA, Thompson JL, Sebire SJ: **Parents' perspectives of change in child physical activity & screen-viewing between Y1 (5-6) & Y4 (8-9) of primary school: implications for behaviour change.** *BMC Public Health* 2018, **18:**520.

For peer review only

For peer review only - http://bmjopen.bmj.com/site/about/guidelines.xhtml

37. Jago R, Sebire SJ, Turner KM, Bentley GF, Goodred JK, Fox KR, Stewart-Brown S, Lucas PJ: **Feasibility trial evaluation of a physical activity and screen-viewing course for parents of 6 to 8 year-old children: Teamplay.** *Int J Behav Nutr Phys Act* 2013, **10:**31.

38. Rutter H, Savona N, Glonti K, Bibby J, Cummins S, Finegood DT, Greaves F, Harper L, Hawe P, Moore L, et al: **The need for a complex systems model of evidence for public health.** *The Lancet* 2017, **390:**2602-2604.

39. **Gartner Says Worldwide Tablet Sales Grew 68 Percent in 2013, With Android Capturing 62 Percent of the Market** [https://www.gartner.com/newsroom/id/2674215]

40. **Worldwide Shipments of Slate Tablets Continue to Decline While Detachable Tablets Climb to New High, According to IDC** [https://web.archive.org/web/20160204014026/http://www.idc.com/getdoc.jsp?containerId=prUS40990116]

For peer review only

Table 1 – Cross-sectional associations between socioeconomic position and screen-viewing at age 6

	Model 1: unadjusted			Model 2: adjusted[1]		
	Ratio	95% CI	p-value	Ratio	95% CI	p-value
**WEEKDAY**		N=1043			N=953	
Household education						
Up to GCSE	1	(Reference)		1	(Reference)	
A level	0.94	(0.85, 1.03)		0.98	(0.89, 1.08)	
Degree	0.79	(0.70, 0.90)		0.88	(0.78, 1.00)	
Higher degree	0.73	(0.60, 0.88)	<0.0005[2]	0.88	(0.74, 1.05)	0.054[2]
Deprivation (IMD)[3]	1.04	(1.00, 1.08)	0.067	1.02	(0.98, 1.07)	0.296
**WEEKEND**		N=1038			N=946	
Household education						
Up to GCSE	1	(Reference)		1	(Reference)	
A level	1.00	(0.91, 1.10)		1.03	(0.95, 1.12)	
Degree	0.88	(0.79, 0.98)		1.02	(0.93, 1.13)	
Higher degree	0.77	(0.67, 0.90)	0.001[2]	0.99	(0.87, 1.11)	0.725[2]
Deprivation (IMD)[3]	1.02	(0.98, 1.06)	0.387	1.01	(0.96, 1.06)	0.720

[1] Adjusted for child gender, child BMI, presence of TVs, computers, tablets and games consoles in the household, parental screen-viewing and parental limiting of screen-viewing

[2] P-value: test for differences between education categories

[3] increase per 1 standard deviation (14.0) in IMD: higher values indicate more deprived areas

For peer review only

For peer review only - http://bmjopen.bmj.com/site/about/guidelines.xhtml

Table 2 – Longitudinal associations between socioeconomic position and screen-viewing at age 9, adjusting for baseline screen-viewing at age 6

	Model 3: unadjusted			Model 4: adjusted[1]		
	Ratio	95% CI	p-value	Ratio	95% CI	p-value
**WEEKDAY**		N=487			N=476	
Household education						
Up to GCSE	1	(Reference)		1	(Reference)	
A level	1.13	(1.01, 1.26)		1.16	(1.03, 1.32)	
Degree	1.06	(0.92, 1.22)		1.10	(0.96, 1.26)	
Higher degree	0.84	(0.70, 0.99)	0.001[2]	0.90	(0.77, 1.05)	0.008[2]
Deprivation (IMD)[3]	1.06	(1.00, 1.13)	0.053	1.05	(1.00, 1.10)	0.038
**WEEKEND**		N=483			N=469	
Household education						
Up to GCSE	1	(Reference)		1	(Reference)	
A level	1.14	(1.00, 1.30)		1.14	(1.01, 1.28)	
Degree	1.09	(0.96, 1.23)		1.11	(0.99, 1.24)	
Higher degree	0.96	(0.83, 1.10)	0.004[2]	0.98	(0.87, 1.11)	0.006[2]
Deprivation (IMD)[3]	1.02	(0.98, 1.07)	0.346	1.00	(0.95, 1.05)	0.938

[1] Adjusted for child gender, presence of TVs, computers, tablets and games consoles in the household, parental screen-viewing and parental limiting of screen-viewing

[2] P-value: test for differences between education categories

[3] increase per 1 standard deviation (14.0) in IMD: higher values indicate more deprived areas

For peer review only

FIGURE LEGENDS AND KEYS

Figure 1: Boxplots of children's total minutes screen-viewing by household education at ages 6 and 9, for weekdays (left) and weekends (right).

Figure 2: Average number of devices in the home by household education at ages 6 (left) and 9 (right).

For peer review only

For peer review only - http://bmjopen.bmj.com/site/about/guidelines.xhtml

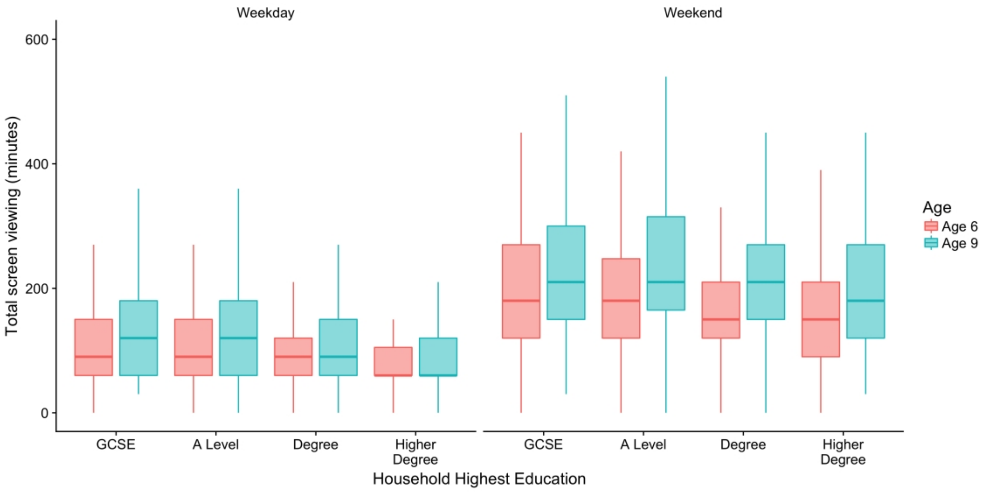

Boxplots of children's total minutes screen-viewing by household education at ages 6 and 9, for weekdays (left) and weekends (right).

1270x635mm (72 x 72 DPI)

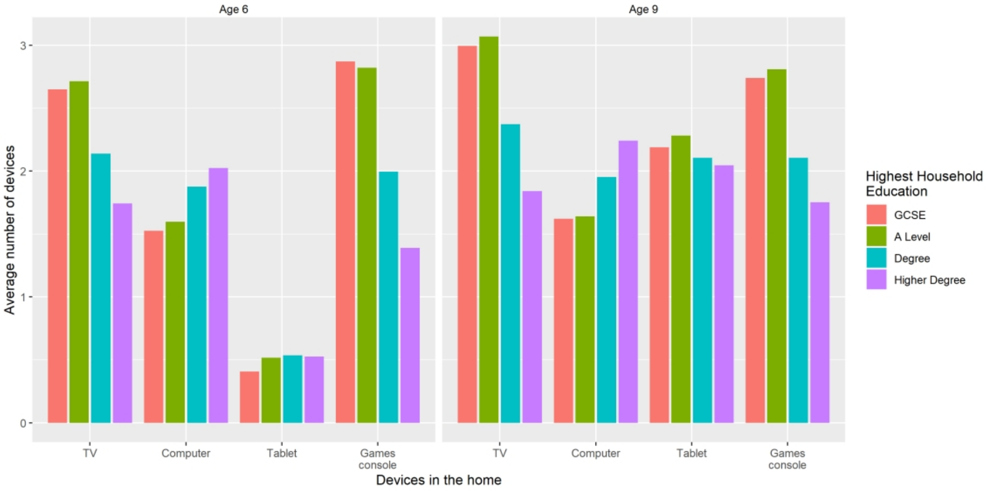

Average number of devices in the home by household education at ages 6 (left) and 9 (right).

SUPPLEMENTARY MATERIAL

ASSOCIATIONS BETWEEN SOCIO-ECONOMIC POSITION AND CHANGES IN CHILDREN'S SCREEN-VIEWING BETWEEN AGES 6 AND 9: A LONGITUDINAL STUDY

Ruth Salway, Lydia Emm-Collison, Simon J. Sebire, Janice L. Thompson and Russell Jago

For peer review only

List of Supplementary Tables

Table S1 – Characteristics at age 6

Table S2 - Characteristics at age 9

Table S3 – Missing values

Table S4 – full details of adjusted model for screen-viewing at age 6

Table S5 – full details of adjusted model for screen-viewing at age 9

Table S6– Associations between socioeconomic position and multi-screen-viewing at age 9

List of Supplementary Figures

Figure S1: Number of minutes spent multi-screen viewing by household education at age 9, for weekdays (left) and weekends (right).

Table S1 – Characteristics at age 6

	Up to GCSE or equiv	A Levels or equiv	Degree or equiv	Higher Degree or equiv	All
	Mean (sd) or %	Mean (sd) or %	Mean (sd) or %	Mean (sd) or %	Mean (sd) or %
WEEKDAY					
Total SV (mins)	120 (74)	111 (61)	93 (60)	85 (70)	102 (66)
TV (mins)	73 (53)	69 (45)	60 (41)	53 (44)	64 (46)
Computer (mins)	25 (28)	23 (27)	20 (22)	23 (33)	22 (26)
Games consoles (mins)	23 (30)	18 (20)	14 (25)	10 (20)	16 (24)
WEEKEND					
Total SV (mins)	196 (118)	193 (107)	169 (94)	150 (102)	178 (105)
TV (mins)	117 (78)	120 (72)	110 (69)	89 (68)	111 (72)
Computer (mins)	37 (44)	34 (38)	32 (33)	37 (39)	34 (37)
Games consoles (mins)	43 (55)	40 (45)	28 (39)	24 (39)	33 (45)
IMD	19.0 (15.5)	16.6 (13.7)	12.2 (10.4)	10.4 (7.4)	14.6 (12.5)
Female	45%	47%	50%	47%	48%
BMI z-score	0.49 (1.04)	0.18 (0.88)	0.16 (0.88)	0.24 (0.94)	0.23 (0.93)
No. devices in home:					
TVs	2.7 (1.3)	2.6 (1.3)	2.2 (1.1)	1.7 (1.1)	2.3 (1.2)
Computers	1.6 (1.1)	1.6 (0.9)	1.9 (1.0)	2.0 (1.1)	1.8 (1.0)
Tablets	0.4 (0.7)	0.5 (0.8)	0.5 (0.7)	0.5 (0.7)	0.5 (0.7)
Games consoles	2.8 (1.8)	2.8 (1.7)	2.0 (1.6)	1.3 (1.3)	2.3 (1.7)
Parent SV (mins)					
Weekday	231 (141)	230 (134)	220 (112)	202 (115)	222 (125)
Weekend	253 (146)	258 (124)	228 (103)	209 (113)	235 (120)
Parental limits on SV[1]	3.3 (0.6)	3.3 (0.6)	3.4 (0.6)	3.5 (0.6)	3.3 (0.6)

[1] higher values indicate more limits on screen viewing (between 1 and 4)

For peer review only

Table S2 - Characteristics at age 9

	Up to GCSE or equiv	A Levels or equiv	Degree or equiv	Higher Degree or equiv	All
	Mean (sd) or %	Mean (sd) or %	Mean (sd) or %	Mean (sd) or %	Mean (sd) or %
WEEKDAY					
Total SV (mins)	139 (94)	133 (78)	113 (75)	88 (59)	118 (78)
TV (mins)	75 (55)	72 (49)	63 (46)	48 (38)	65 (48)
Computer (mins)	37 (46)	34 (37)	31 (37)	29 (31)	32 (37)
Games consoles (mins)	28 (44)	26 (34)	19 (30)	12 (24)	21 (33)
Multi-screen viewing (mins)	29 (46)	24 (40)	16 (33)	11 (25)	19 (37)
WEEKEND					
Total SV (mins)	244 (134)	245 (120)	227 (118)	200 (118)	230 (122)
TV (mins)	127 (72)	126 (66)	125 (63)	104 (62)	122 (65)
Computer (mins)	59 (66)	58 (64)	53 (60)	57 (54)	56 (61)
Games consoles (mins)	58 (68)	62 (63)	49 (62)	39 (54)	52 (62)
Multi-screen viewing (mins)	53 (73)	48 (66)	33 (61)	23 (45)	39 (63)
IMD	20.1 (15.8)	17.6 (15.0)	12.8 (11.9)	12.0 (10.3)	15.1 (13.6)
Female	56%	52%	56%	53%	55%
BMI z-score	0.44 (1.15)	0.28 (1.04)	0.22 (1.06)	0.28 (1.02)	0.31 (1.06)
No. devices in the home					
TVs	2.9 (1.3)	3.1 (1.4)	2.4 (1.3)	1.8 (1.1)	2.6 (1.4)
Computers	1.6 (1.2)	1.7 (1.1)	2.0 (1.2)	2.2 (1.1)	1.9 (1.2)
Tablets	2.1 (1.2)	2.3 (1.3)	2.1 (1.2)	2.0 (1.2)	2.2 (1.3)
Games consoles	2.6 (2.1)	2.8 (1.8)	2.2 (1.6)	1.7 (1.6)	2.3 (1.8)
Parent SV (mins)					
Weekday	248 (134)	245 (128)	248 (134)	253 (144)	248 (134)
Weekend	280 (154)	287 (135)	260 (130)	252 (144)	270 (138)
Parental limits on SV[1]	3.1 (0.7)	3.2 (0.6)	3.4 (0.6)	3.4 (0.5)	3.3 (0.6)

[1] higher values indicate more limits on screen viewing (between 1 and 4)

For peer review only

Table S3 – Missing values

	Age 6		Age 9		Data at both time points: Age 6		Data at both time points: Age 9	
	N	% missing	N	% missing	N	% missing	N	% missing
Total	1085		997		509		509	
Household education	1071	1%	992	0.2%	509	0%	509	0%
IMD	1069	1%	983	1%	501	2%	499	2%
**WEEKDAY**								
Total SV (mins)	1069	1%	979	2%	504	1%	502	1%
Multi-SV (mins)			981	2%			503	1%
Parent SV (mins)	1075	1%	981	2%	505	1%	503	1%
Parent multi-SV (mins)			983	1%			503	1%
**WEEKEND**								
Total SV (mins)	1065	2%	978	2%	500	2%	501	2%
Multi-SV (mins)			981	2%			503	1%
Parent SV (mins)	1068	2%	979	2%	505	1%	501	2%
Parent multi-SV (mins)			983	1%			503	1%
Gender	1085	0%	997	0%	509	0%	509	0%
BMI z-score	1072	1%	993	0.4%	502	1%	508	0.2%
No. TVs	1073	1%	982	2%	507	0.4%	503	1%
No. Computers	1058	2%	982	2%	504	1%	503	1%
No. Tablets	1062	2%	981	2%	504	1%	503	1%
No. games consoles	1056	3%	981	2%	500	2%	503	1%
Parental limits on SV	1064	2%	971	3%	495	3%	497	2%

For peer review only

Table S4 – full details of adjusted model for screen-viewing at age 6

	WEEKDAY			WEEKEND		
	Ratio	95% CI	p-value	Ratio	95% CI	p-value
Household education						
Up to GCSE	1	(Reference)		1	(Reference)	
A level	0.98	(0.89, 1.08)		1.03	(0.95, 1.12)	
Degree	0.88	(0.78, 1.00)		1.02	(0.93, 1.13)	
Higher degree	0.88	(0.74, 1.05)	0.054	0.99	(0.87, 1.11)	0.725
Deprivation (IMD)[1]	1.02	(0.98, 1.07)	0.296	1.01	(0.96, 1.06)	0.720
>1 TV	0.99	(0.90, 1.10)	0.924	1.04	(0.95, 1.13)	0.394
Any computers	0.94	(0.74, 1.21)	0.645	0.86	(0.73, 1.02)	0.080
Any tablets	1.02	(0.94, 1.10)	0.652	1.01	(0.94, 1.08)	0.811
Any games consoles	1.15	(1.00, 1.32)	0.043	1.42	(1.26, 1.60)	<0.0005
Child gender	0.87	(0.81, 0.94)	<0.0005	0.82	(0.78, 0.87)	<0.0005
z-BMI score	1.07	(1.02, 1.12)	0.003	1.04	(1.01, 1.07)	0.021
parent SV (mins)[2]	1.04	(1.03, 1.05)	<0.0005	1.05	(1.04, 1.07)	<0.0005
Parental limits on SV	0.77	(0.72, 0.83)	<0.0005	0.85	(0.81, 0.90)	<0.0005

[1] increase per 1 standard deviation (14.0) in IMD: higher values indicate more deprived areas
[2] increase per 30 minutes screen-viewing

For peer review only

Table S5 – full details of adjusted model for screen-viewing at age 9

	WEEKDAY			WEEKEND		
	Ratio	95% CI	p-value	Ratio	95% CI	p-value
Household education						
Up to GCSE	1	(Reference)		1	(Reference)	
A level	1.16	(1.03, 1.32)		1.14	(1.01, 1.28)	
Degree	1.10	(0.96, 1.26)		1.11	(0.99, 1.24)	
Higher degree	0.90	(0.77, 1.05)	0.008	0.98	(0.87, 1.11)	0.006
Deprivation (IMD)[1]	1.05	(1.00, 1.10)	0.038	1.00	(0.95, 1.05)	0.938
Baseline SV [2]	1.08	(1.05, 1.10)	<0.0005	1.05	(1.03, 1.06)	<0.0005
>1 TV	1.16	(1.06, 1.27)	0.001	1.06	(0.96, 1.18)	0.249
Any computers	1.03	(0.86, 1.24)	0.721	1.07	(0.92, 1.26)	0.386
Any tablets	0.75	(0.64, 0.88)	<0.0005	0.80	(0.68, 0.95)	0.009
Any games consoles	1.26	(1.11, 1.44)	<0.0005	1.27	(1.08, 1.49)	0.003
Child gender	0.98	(0.91, 1.06)	0.517	0.89	(0.83, 0.96)	0.002
z-BMI score	1.00	(0.95, 1.05)	0.966	1.00	(0.96, 1.03)	0.904
parent SV (mins)[2]	1.03	(1.02, 1.04)	<0.0005	1.04	(1.03, 1.05)	<0.0005
Parental limits on SV	0.82	(0.76, 0.89)	<0.0005	0.93	(0.86, 1.01)	0.084

[1] increase per 1 standard deviation (14.0) in IMD: higher values indicate more deprived areas

[2] increase per 30 minutes screen-viewing at age 6

[3] increase per 30 minutes screen-viewing

For peer review only

Table S6– Associations between socioeconomic position and multi-screen-viewing at age 9

	WEEKDAY			WEEKEND		
	Ratio	95% CI	p-value	Ratio	95% CI	p-value
**Unadjusted Model**						
Household education						
Up to GCSE	1	(Reference)		1	(Reference)	
A level	0.82	(0.49, 1.37)		0.87	(0.53, 1.42)	
Degree	0.66	(0.38, 1.15)		0.63	(0.37, 1.06)	
Higher degree	0.52	(0.26, 1.05)	0.226	0.55	(0.31, 0.97)	0.083
Deprivation (IMD)[1]	1.42	(1.21, 1.67)	<0.0005	1.33	(1.16, 1.52)	<0.0005
**Adjusted Model[2]**						
Household education						
Up to GCSE	1	(Reference)		1	(Reference)	
A level	0.92	(0.51, 1.65)		0.90	(0.50, 1.59)	
Degree	0.80	(0.45, 1.42)		0.72	(0.38, 1.34)	
Higher degree	0.76	(0.34, 1.71)	0.808	0.64	(0.33, 1.25)	0.432
Deprivation (IMD)[1]	1.26	(1.12, 1.41)	<0.0005	1.27	(1.13, 1.43)	<0.0005
>1 TV	1.76	(1.12, 2.75)	0.014	1.38	(0.88, 2.16)	0.158
Any computers	0.70	(0.41, 1.20)	0.190	0.97	(0.54, 1.75)	0.920
Any tablets	1.90	(1.12, 3.22)	0.017	1.34	(0.46, 3.88)	0.590
Any games consoles	1.02	(0.59, 1.78)	0.943	1.65	(0.95, 2.85)	0.076
Child gender	1.01	(0.73, 1.40)	0.945	1.06	(0.76, 1.49)	0.730
z-BMI score	1.08	(0.92, 1.25)	0.348	0.93	(0.82, 1.05)	0.246
parent multi-SV (mins)[2]	1.26	(1.17, 1.35)	<0.0005	1.21	(1.13, 1.29)	<0.0005
Parental limits on SV	0.62	(0.50, 0.78)	<0.0005	0.72	(0.53, 0.97)	0.033

[1] increase per 1 standard deviation (14.0) in IMD: higher values indicate more deprived areas
[2] Adjusted for child gender, child BMI, presence of TVs, computers, tablets and games consoles in the household, parental screen-viewing and parental limiting of screen-viewing
[3] increase per 30 minutes multi-screen-viewing

For peer review only

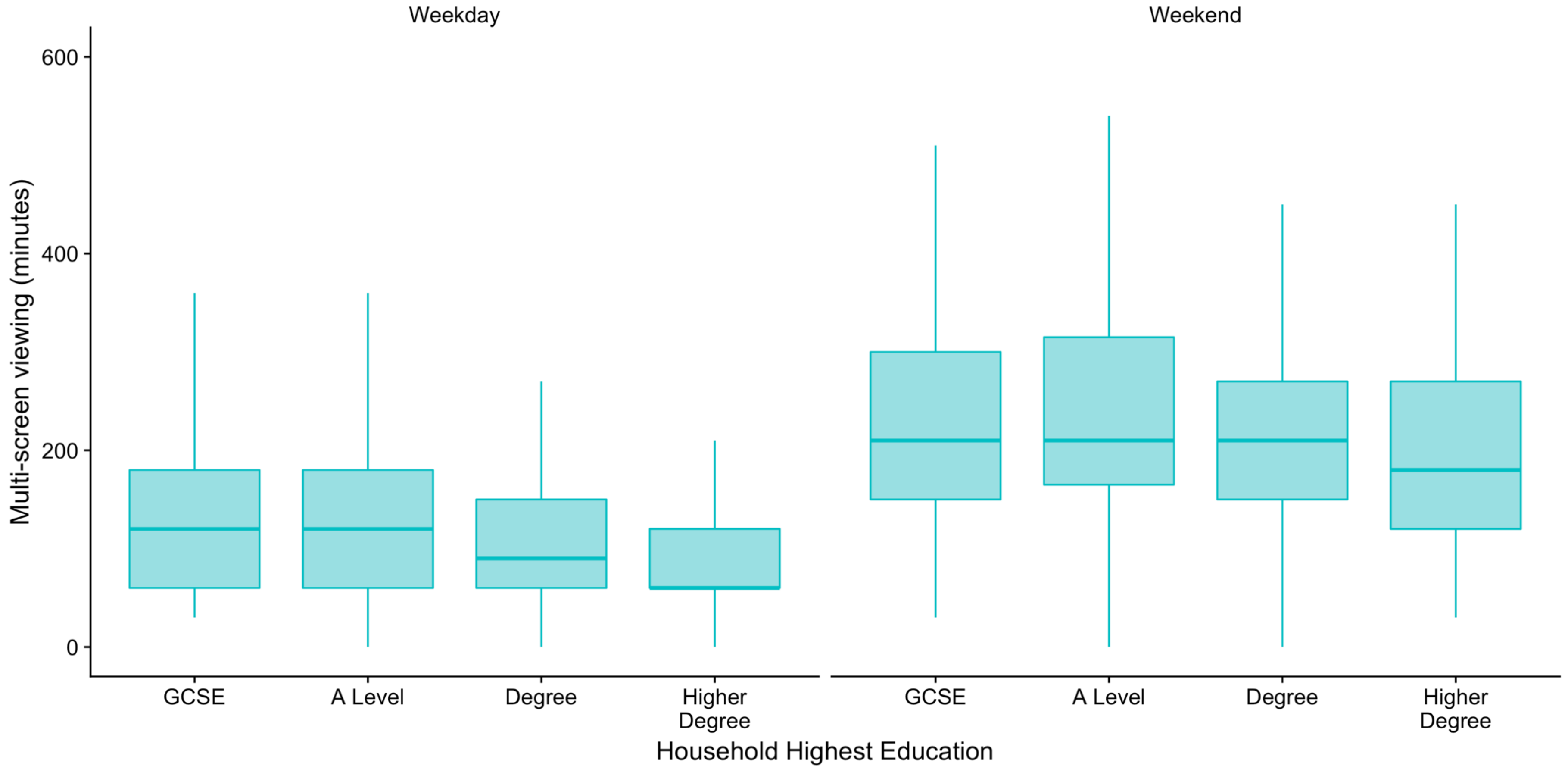

Figure S1: Number of minutes spent multi-screen viewing by household education at age 9, for weekdays (left) and weekends (right).

STROBE 2007 (v4) Statement—Checklist of items that should be included in reports of *cohort studies*

Section/Topic	Item #	Recommendation	Reported on page #
**Title and abstract**	1	(*a*) Indicate the study's design with a commonly used term in the title or the abstract	Title page
		(*b*) Provide in the abstract an informative and balanced summary of what was done and what was found	2-3
**Introduction**			
Background/rationale	2	Explain the scientific background and rationale for the investigation being reported	5-6
Objectives	3	State specific objectives, including any prespecified hypotheses	6
**Methods**			
Study design	4	Present key elements of study design early in the paper	6-7
Setting	5	Describe the setting, locations, and relevant dates, including periods of recruitment, exposure, follow-up, and data collection	6-7
Participants	6	(*a*) Give the eligibility criteria, and the sources and methods of selection of participants. Describe methods of follow-up	6-7
		(*b*) For matched studies, give matching criteria and number of exposed and unexposed	n/a
Variables	7	Clearly define all outcomes, exposures, predictors, potential confounders, and effect modifiers. Give diagnostic criteria, if applicable	7-8
Data sources/ measurement	8*	For each variable of interest, give sources of data and details of methods of assessment (measurement). Describe comparability of assessment methods if there is more than one group	7-8
Bias	9	Describe any efforts to address potential sources of bias	8-9
Study size	10	Explain how the study size was arrived at	6-7
Quantitative variables	11	Explain how quantitative variables were handled in the analyses. If applicable, describe which groupings were chosen and why	8-9
Statistical methods	12	(*a*) Describe all statistical methods, including those used to control for confounding	8-9
		(*b*) Describe any methods used to examine subgroups and interactions	8-9
		(*c*) Explain how missing data were addressed	9
		(*d*) If applicable, explain how loss to follow-up was addressed	n/a
		(*e*) Describe any sensitivity analyses	n/a
**Results**			

For peer review only

Participants	13*	(a) Report numbers of individuals at each stage of study—eg numbers potentially eligible, examined for eligibility, confirmed eligible, included in the study, completing follow-up, and analysed	7
		(b) Give reasons for non-participation at each stage	n/a
		(c) Consider use of a flow diagram	
Descriptive data	14*	(a) Give characteristics of study participants (eg demographic, clinical, social) and information on exposures and potential confounders	9-11
		(b) Indicate number of participants with missing data for each variable of interest	9
		(c) Summarise follow-up time (eg, average and total amount)	n/a
Outcome data	15*	Report numbers of outcome events or summary measures over time	9-10
Main results	16	(*a*) Give unadjusted estimates and, if applicable, confounder-adjusted estimates and their precision (eg, 95% confidence interval). Make clear which confounders were adjusted for and why they were included	11-12
		(*b*) Report category boundaries when continuous variables were categorized	n/a
		(*c*) If relevant, consider translating estimates of relative risk into absolute risk for a meaningful time period	n/a
Other analyses	17	Report other analyses done—eg analyses of subgroups and interactions, and sensitivity analyses	
**Discussion**			
Key results	18	Summarise key results with reference to study objectives	12-13
**Limitations**			
Interpretation	20	Give a cautious overall interpretation of results considering objectives, limitations, multiplicity of analyses, results from similar studies, and other relevant evidence	13-14
Generalisability	21	Discuss the generalisability (external validity) of the study results	14-15
**Other information**			
Funding	22	Give the source of funding and the role of the funders for the present study and, if applicable, for the original study on which the present article is based	16

*Give information separately for cases and controls in case-control studies and, if applicable, for exposed and unexposed groups in cohort and cross-sectional studies.

Note: An Explanation and Elaboration article discusses each checklist item and gives methodological background and published examples of transparent reporting. The STROBE checklist is best used in conjunction with this article (freely available on the Web sites of PLoS Medicine at http://www.plosmedicine.org/, Annals of Internal Medicine at http://www.annals.org/, and Epidemiology at http://www.epidem.com/). Information on the STROBE Initiative is available at www.strobe-statement.org.

For peer review only
